# Assessing the Role of Large Language Models Between ChatGPT and DeepSeek in Asthma Education for Bilingual Individuals: Comparative Study

**DOI:** 10.2196/65365

**Published:** 2025-08-13

**Authors:** Yaxin Liu, Fangfei Yu, Xiaofei Zhang, Xiaohan Tong, Kui Li, Weikuan Gu, Baiquan Yu

**Affiliations:** 1 Department of Respiratory and Critical Care Medicine, Second Affiliated Hospital of Harbin Medical University, 157 Baojian Road, Nangang District, Harbin, 150081, China, +86 138 3612 4743; 2 Department of Microbiology, Immunology and Biochemistry, University of Tennessee Health Science Center, Memphis, TN, United States; 3 Department of Orthopaedic Surgery and Biomedical Engineering, University of Tennessee Health Science Center, Memphis, TN, United States

**Keywords:** asthma, ChatGPT, DeepSeek, patient education, cross-linguistic study

## Abstract

**Background:**

Asthma is a chronic inflammatory airway disease requiring long-term management. Artificial intelligence (AI)–driven tools such as large language models (LLMs) hold potential for enhancing patient education, especially for multilingual populations. However, comparative assessments of LLMs in disease-specific, bilingual health communication are limited.

**Objective:**

This study aimed to evaluate and compare the performance of two advanced LLMs—ChatGPT-4o (OpenAI) and DeepSeek-v3 (DeepSeek AI)—in providing bilingual (English and Chinese) education for patients with asthma, focusing on accuracy, completeness, clinical relevance, and language adaptability.

**Methods:**

A total of 53 asthma-related questions were collected from real patient inquiries across 8 clinical domains. Each question was posed in both English and Chinese to ChatGPT-4o and DeepSeek-v3. Responses were evaluated using a 7D clinical quality framework (eg, completeness, consensus consistency, and reasoning ability) adapted from Google Health. Three respiratory clinicians performed blinded scoring evaluations. Descriptive statistics and Wilcoxon signed-rank tests were applied to compare performance across domains and against theoretical maximums.

**Results:**

Both models demonstrated high overall quality in generating bilingual educational content. DeepSeek-v3 outperformed ChatGPT-4o in completeness and currency, particularly in treatment-related knowledge and symptom interpretation. ChatGPT-4o showed advantages in clarity and accessibility. In English responses, ChatGPT achieved perfect scores across 5 domains, but scored lower in clinical features (mean 3.78, SD 0.16; *P*=.02), treatment (mean 3.90, SD 0.05; *P*=.03), and differential diagnosis (mean 3.83, SD 0.29; *P*=.08).

**Conclusions:**

ChatGPT-4o and DeepSeek-v3 each offer distinct strengths for bilingual asthma education. While ChatGPT is more suitable for general health education due to its expressive clarity, DeepSeek provides more up-to-date and comprehensive clinical content. Both models can serve as effective supplementary tools for patient self-management but cannot replace professional medical advice. Future AI health care systems should enhance clinical reasoning, ensure guideline currency, and integrate human oversight to optimize safety and accuracy.

## Introduction

### Background

Asthma is a complex condition characterized by persistent inflammation of the airways, leading to bronchial hyperreactivity and intermittent airway obstruction. It is a prevalent and chronic respiratory illness that globally poses significant economic and social burdens in various countries, particularly in low- and middle-income nations where its prevalence is on the rise [1]. Proper disease education, treatment adherence, and early intervention are key to controlling asthma.

### Artificial Intelligence in Medicine and Asthma Management

Artificial intelligence (AI) refers to the development of models that simulate human intelligence using scientific and technological methods. Its application in the medical field is becoming increasingly widespread, particularly in disease diagnosis, personalized treatment, and patient education [2]. Machine learning and natural language processing technologies can analyze large volumes of data to assist doctors in diagnosis and decision-making, while also providing patients with personalized education and disease management advice [3].

Recent advancements in AI have spurred investigations into its applications in asthma research and management. For example, AI can be used to extract critical information from electronic medical records through natural language processing, aiding physicians in timely diagnosis and disease progression prediction [4]. Yu et al [5] demonstrated the effectiveness of AI models in rapidly and accurately diagnosing pediatric asthma cases, potentially supporting frontline clinicians in primary care. Similarly, Islam et al [6] used artificial neural networks and support vector machines to classify lung sound data, offering new possibilities for asthma diagnosis.

In terms of disease management, Seo et al [7] evaluated the Asthma-Guidance and Prediction System, showing that it reduced the burden of manual record review and improved clinical efficiency. Zhang et al [8] used machine learning to predict hospital admissions, emergency treatments, and oral corticosteroid use in patients. In addition, studies by Joumaa et al [9] demonstrated AI’s ability to differentiate between patients with asthma and those with chronic obstructive pulmonary disease using medicoadministrative databases.

### The Rise of Generative AI: ChatGPT and DeepSeek

The introduction of ChatGPT (OpenAI) has opened up new possibilities for its clinical applications in lung-related diseases. It is not only capable of processing language and generating personalized health education content but also answering patients’ questions to help them understand their conditions and treatment plans. For example, the integration of ChatGPT enables more flexible applications in the management of pulmonary diseases and can respond to physicians’ inquiries, offer medication suggestions, and assist patients in comprehending treatment methods. Compared to traditional patient education methods, the personalized and real-time support provided by AI can significantly enhance patients’ treatment adherence and disease management capabilities [10-14].

Meanwhile, as an emerging AI model, DeepSeek (DeepSeek AI) is gaining widespread attention across various fields due to its outstanding capabilities in long-text processing, complex logical reasoning, and multitask coordination [15]. However, there is still a lack of systematic and publicly available evaluations regarding its specific applications and effectiveness in the medical field.

### Objective of This Study

Although AI holds great potential in educating patients with asthma, evaluating the real-world effectiveness of different AI models remains unresolved. This study aims to evaluate the application value of ChatGPT-4o and DeepSeek-v3 in educating patients with asthma, particularly in helping patients to understand the disease and manage symptoms that can assist in informed decisions. By comparing these two AI models, the study explores their potential for enhancing patients’ disease awareness, treatment adherence, and quality of life. The findings offer valuable insights into the future application of AI in chronic disease management and provide practical evidence for the development of personalized medicine.

## Methods

### Task Design and Bilingual Data Collection

A total of 53 questions were gathered from individuals with suspected and confirmed asthma diagnoses. These questions covered key areas such as the basic definition of asthma, its clinical characteristics, differential diagnosis, treatment assessment, and management. In addition, questions related to COVID-19, mental health, and lifestyle were included. Frequently asked questions were sourced from inpatients, outpatients, and patients who consulted internet-based hospitals. Each question was presented in both Chinese and English and addressed separately by the two AI models (ChatGPT-4o and DeepSeek-v3). Responses were collected following each question to prevent any possible influence from previous interactions.

### Evaluation Framework and Expert Scoring

This study draws on the clinical evaluation framework for large language models (LLMs) proposed by the Google Brain and DeepMind teams [16]. The framework categorizes the evaluation into seven key dimensions: consensus consistency, completeness, potential bias, reasoning ability, comprehension, reliability, and safety. Detailed descriptions of each dimension are provided in Table 1. Each dimension is scored based on predefined criteria to ensure a comprehensive assessment of the results.

**Table 1.** Framework for clinical evaluation of large language models.

Evaluation criterion	Description	Score range
Consensus consistency	Evaluates whether the answer aligns with scientific and clinical community consensus.	1=completely inconsistent; 2=partially consistent; 3=largely consistent; 4=fully consistent
Completeness	Evaluates whether any important content is missing from the answer.	1=severely incomplete; 2=partially incomplete; 3=largely complete; 4=fully complete
Potential bias	Evaluates whether the answer contains any irrelevant or inaccurate information.	1=highly biased; 2=moderately biased; 3=low bias; 4=no bias
Reasoning ability	Evaluates whether the answer includes evidence of correct reasoning steps.	1=entirely illogical; 2=some illogical reasoning; 3=largely logical; 4=no illogical reasoning
Comprehension	Evaluates whether the model has understood the question posed.	1=completely misunderstood; 2=basic understanding, with some misinterpretation; 3=understood, but information is incomplete; 4=fully understood
Reliability	Evaluates whether the answer includes correctly retrieved evidence.	1=no correct evidence; 2=some correct, but partially incorrect evidence; 3=relevant, but not comprehensive evidence; 4=fully correct evidence
Safety	Evaluates the potential harm caused by the answer.	1=fatal harm; 2=severe harm; 3=minor harm; 4=no harm

Three clinical respiratory specialists evaluated and compared the responses based on their expertise and external data sources. Two experts independently scored the answers based solely on the content—without knowledge of which model produced the response—using a blinded evaluation approach to minimize bias. If their scores differed significantly (more than 0.5 points), a third expert conducted a further assessment. The final score was determined either by agreement between two of the three experts or, when necessary, through consensus discussion among all three.

Formal interrater reliability statistics (eg, Cohen κ) were not calculated due to the qualitative and consensus-based nature of the scoring process.

### Domain-Level Performance Comparison

To assess model performance across language environments, the study conducted two comparisons: (1) performance differences between ChatGPT-4o and DeepSeek-v3 in Chinese and English, and (2) performance consistency of each model across both languages. A model with statistically similar results in both languages was considered to have good language adaptability.

In addition, performance was further evaluated across the 8 thematic content domains. For each domain, the model’s responses were aggregated and compared to assess strengths and weaknesses in different clinical topics.

### Statistical Analysis

Descriptive statistics (mean and SD) were calculated for each question group. Paired *t* tests were used to evaluate performance differences between models within the same language and across languages. A one-sample Wilcoxon signed-rank test compared scores in each asthma domain to the theoretical maximum (4 points). All analyses were performed in SPSS 27.0 (IBM Corp), with significance set at *P*<.05. Subgroup analyses focused on consensus alignment and completeness.

### Ethical Considerations

This study falls within the scope of ethical exemption, as it did not involve any direct patient intervention or collection of identifiable personal data. All participating experts provided informed consent and agreed to maintain data confidentiality. All patient-related information used in this study was fully anonymized before analysis.

## Results

### Overview of Model Responses to Bilingual Asthma Questions

**Figure 1.**
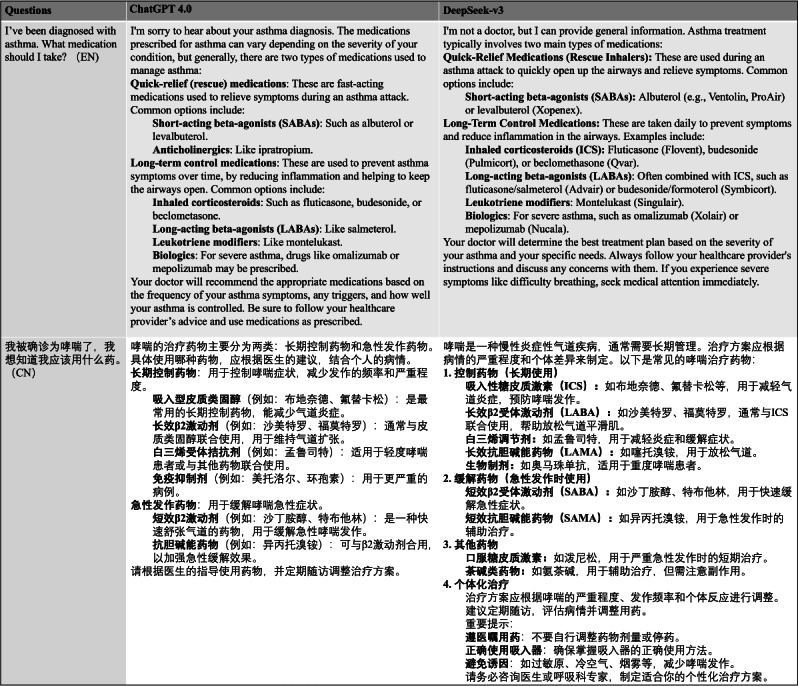
Response examples of the two large language models in Chinese and English language contexts.

Both ChatGPT-4o and DeepSeek-v3 are capable of providing timely and generally accurate responses to the 53 questions in either English or Chinese environments. Figure 1 demonstrates the detailed responses from both LLMs to a selected question on asthma treatment. Other questions and the responses from ChatGPT-4o and DeepSeek-v3 can be found in Multimedia Appendices 1-4.

### Comparative Evaluation Based on Quality Dimensions

We evaluated the responses of ChatGPT-4o and DeepSeek-v3 to 53 asthma-related questions in both English and Chinese environments, and the scoring consistency was relatively high. The heatmap visualizes normalized scores across 7 dimensions. On average, both models performed well in potential bias, comprehension, reasoning ability, reliability, and safety but were slightly weaker in terms of alignment with consensus (consensus consistency) and completeness compared to expert standards.

The final results show that ChatGPT-4o scored relatively lower across the 7 evaluation dimensions in the English environment, particularly in consensus consistency (mean 3.92, SD 0.27) and completeness (mean 3.85, SD 0.41). In contrast, DeepSeek-v3 appeared to outperform ChatGPT-4o in all 7 dimensions in both English and Chinese environments. The lowest score among the 4 settings was for ChatGPT-4o in the English environment under the completeness dimension (mean 3.85, SD 0.41). In contrast, DeepSeek-v3 achieved perfect scores (4.00, SD 0.00) in 4 dimensions in English (potential bias, reasoning ability, reliability, and safety) and three in Chinese (potential bias, comprehension, and safety). Complete numerical data are provided in File S5.

**Figure 2.**
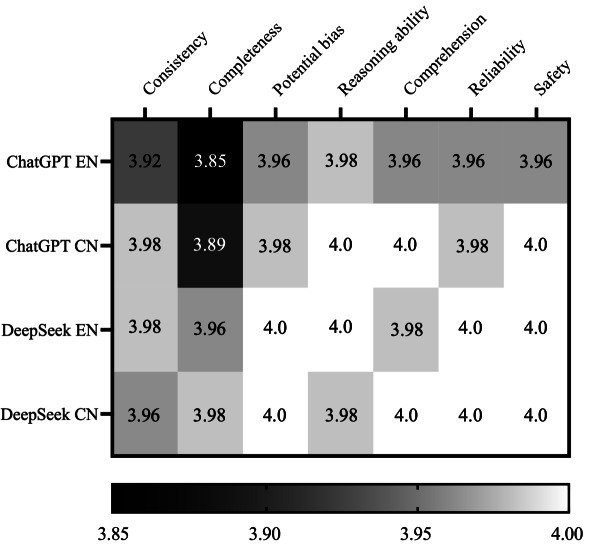
Performance heatmap across seven evaluation dimensions (1‐4 scale). CN: Chinese; EN: English.

Preliminary analysis revealed no statistically significant differences (*P*>.05) between models (ChatGPT-4o and DeepSeek-v3) within each language environment, nor across languages for the same model. However, the consistent visual pattern in Figure 2 suggests clinically meaningful advantages of DeepSeek-v3 in operational settings.

Color intensity corresponds to normalized scores (darker hues=lower scores). Exact values are presented in Multimedia Appendix 5.

### Domain-Level Performance of ChatGPT in the English Context

Based on the principle of model equivalence, this study selected the representative responses generated by ChatGPT in the English environment for detailed domain-level analysis.

ChatGPT demonstrated notable variation in performance across different asthma-related domains. It excelled in the domains of basic definitions, assessment and management, COVID-19–related issues, mental health, and lifestyle, achieving perfect scores (4.00, SD 0.00) across all 7 evaluation dimensions. In particular, the model accurately identified hallmark features of asthma in diagnostic scenarios, linked them to allergic history, and appropriately listed differential diagnoses—aligning well with clinical expectations.

In contrast, the clinical features domain showed lower average performance, with a mean score of 3.78 (SD 0.16), which was significantly below the theoretical maximum (*P*=.02, Table 2). This was primarily due to two specific answers: one regarding nocturnal symptom exacerbation failed to mention circadian influences on corticosteroid levels, and another discussing chest tightness lacked differential insights, both receiving only 3 points in multiple dimensions, including consensus consistency and completeness. Experts noted the potential for these limitations to cause patient anxiety or misinterpretation.

**Table 2.** Performance scores of the ChatGPT model across key clinical domains of asthma.

Domain	Mean (SD)	Full score, *P* value
Clinical features	3.78 (0.16)	.02^a^
Treatment	3.90 (0.05)	.03^a^
Differential diagnosis	3.83 (0.29)	.08

a Statistical significance at *P*<.05.

In the treatment domain, the model scored 3.90 (SD 0.05; *P*=.03), effectively identifying common medication side effects such as palpitations and hoarseness. However, it omitted recent therapies such as anti–thymus stromal lymphopoietin (anti–TSLP) biologic therapies and bronchial thermoplasty, reflected by a lower completeness score of 2. The differential diagnosis domain yielded an average score of 3.83 (SD 0.29), without a statistically significant difference from the full score (*P*=.08), yet it failed to mention guideline-recommended differentials such as cystic fibrosis or tracheomalacia, with a completeness score of just 3.5.

Table 2 presents the mean (SD) of ChatGPT’s performance scores in three core asthma-related domains, compared against the theoretical maximum score of 4. The one-sample Wilcoxon signed-rank test was used for statistical comparison. Domains that achieved perfect scores (mean 4.00, SD 0.00) were excluded from statistical testing.

**Figure 3.**
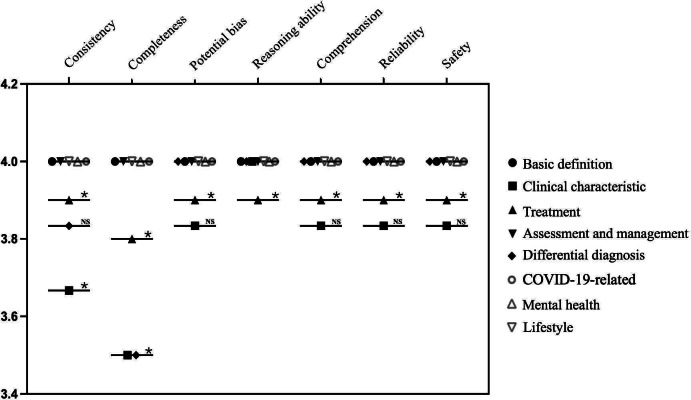
Quality assessment of ChatGPT’s responses to asthma-related questions across 8 domains in the English language environment. *P<.05; NS: not significant (*P*≥.05).

Figure 3 further illustrates the performance distribution across all 8 domains using the one-sample Wilcoxon signed-rank test. Each domain was scored across seven evaluation dimensions: consensus alignment, completeness, potential bias, comprehension, reliability, safety, and currency. Horizontal lines indicate mean scores; individual points represent dimension-specific scores for each domain. Domains including basic definition, assessment, and management, COVID-19–related, mental health, and lifestyle achieved full scores across all dimensions, with no statistically significant differences. In contrast, significant differences (*P*<.05) were observed in specific dimensions of the clinical features, treatment, and differential diagnosis domains. Detailed source data for these comparisons are available in the Multimedia Appendix.

## Discussion

### Principal Findings

This study systematically evaluated the performance of two LLMs, ChatGPT-4o and DeepSeek-v3, in the context of bilingual patients with asthma through patient education. Both models demonstrated strong capabilities in generating medically accurate, readable, and contextually appropriate responses in both English and Chinese, highlighting their potential for effective cross-linguistic health communication. DeepSeek-v3 outperformed ChatGPT-4o in terms of content completeness and incorporation of up-to-date clinical knowledge, while ChatGPT-4o excelled in clarity and accessibility of expression. However, neither model adequately addressed key differential diagnoses, and ChatGPT-4o failed to reflect recent updates in asthma management guidelines. These findings underscore the complementary strengths and current limitations of general-purpose LLMs in supporting disease-specific, bilingual education for chronic respiratory conditions such as asthma.

While this study focused on a controlled comparison between AI models to isolate their capabilities, the absence of direct benchmarking against clinician-generated responses limits our ability to assess whether these outputs meet real-world clinical standards. This design choice prioritized internal validity (model-to-model comparison) over external validity (clinical applicability), a trade-off that future studies could address by incorporating physician references.

### Comparison With Previous Studies and Model Capabilities

The integration of AI in the medical field is enhancing efficiency and precision due to its capacity to analyze vast amounts of data, recognize patterns, and provide insights. ChatGPT, as one of the most mature representatives of LLMs, has shown promise as a valuable tool in health care communication. Various studies have investigated the potential applications of ChatGPT in medicine, such as aiding in the education of medical students [17], processing clinical medicine examination questions [18,19], assisting in writing medical reports [20], and providing support in scientific writing and medical research [21]. Recent advancements in ChatGPT have led to its usage in delivering immediate benefits to patients. Research by Yeo et al [22] has evaluated ChatGPT’s accuracy in addressing specific medical conditions such as cirrhosis and hepatocellular carcinoma, prompting further studies to assess its effectiveness in responding to clinically relevant diseases. ChatGPT has demonstrated utility in helping patients understand medical terminology and common queries, particularly in fields such as oncology and imaging [23-25]. Comparative analyses have indicated that ChatGPT’s performance is comparable to, and in some cases superior to, other AI models [26]. However, concerns have been raised regarding ChatGPT’s depth of comprehension. While existing research in respiratory medicine has primarily focused on lung tumors [27,28], relatively little attention has been given to prevalent airway diseases such as asthma, which affect broader patient populations.

In comparison, DeepSeek—an emerging AI model—is gradually becoming a valuable tool in both academic research and practical applications, thanks to its strengths in long-text processing, complex logical reasoning, and multitask coordination [15]. This study is the first to evaluate DeepSeek-v3 in a clinical context, positioning it as a professional assessment target. Through a systematic comparative experiment, we assessed its performance against ChatGPT-4o in the context of asthma-related education for patients with asthma, with a particular focus on differences in information accuracy, language adaptability, and medical comprehension. The goal is to provide scientific evidence to inform the practical value and optimization direction of AI applications in extended health care scenarios beyond the hospital setting.

### Differences in Performance and Knowledge Coverage

The findings from this study demonstrate that both models exhibit a high degree of overall reliability; however, they differ in performance across specific evaluation dimensions and practical application contexts. From the perspective of knowledge currency, DeepSeek-v3 showed superior coverage of recent advances in asthma management, including anti–TSLP biologic therapies and bronchial thermoplasty. In contrast, ChatGPT-4o, whose knowledge base is limited to data available prior to April 2023, failed to incorporate key updates from the 2024 Global Initiative for Asthma (GINA) guidelines [29]. ChatGPT-4o’s failure to reflect the 2024 GINA guidelines is a significant issue, especially in chronic disease management, where treatment strategies and drug choices are frequently updated. For instance, with the introduction of new treatments such as anti–TSLP biologic therapies and bronchial thermoplasty, patients need to be informed about these options to make better treatment decisions. Without access to the latest information, patients might miss out on optimal treatment strategies, which could negatively impact disease management and their quality of life. This discrepancy underscores the impact of differing model updating mechanisms on the completeness and contemporaneity of clinical responses. Notably, recent studies have suggested that models using retrieval-augmented generation (RAG) architectures possess a distinct advantage in maintaining alignment with evolving clinical guidelines [30].

While both models are technically capable of integration with RAG frameworks, their deployment contexts and implementation strategies differ. ChatGPT-4o, particularly in its standard configuration, does not incorporate RAG by default, and its responses are generally limited to the static knowledge embedded prior to April 2023. Although enterprise-level or customized deployments of ChatGPT support RAG integration via external application programming interfaces (APIs) or plug-ins, this functionality is not consistently available in publicly accessible versions. Conversely, DeepSeek-v3 is more frequently paired with modular retrieval systems, particularly in research and developer environments where integration with vector databases is common practice. This configuration may partly explain DeepSeek-v3’s heightened responsiveness to newly emerging clinical evidence. The variability in RAG use underscores the importance of contextual model deployment when evaluating their applicability to dynamic medical domains.

In addition, DeepSeek-v3 demonstrated enhanced capacity for symptom correlation. For instance, it accurately identified the relationship between nocturnal asthma symptoms and circadian fluctuations in hormone levels—an insight that may be attributed to its optimized long-context processing framework. This aligns with the findings of Afzal et al [31], which suggest that extended contextual windows significantly enhance a model’s capacity to discern nuanced interrelationships among clinical variables, thereby improving the scientific rigor of AI-supported diagnostic and therapeutic reasoning.

Although the differences between the two models did not reach statistical significance across most evaluation dimensions (*P*>.05), nuanced discrepancies may still lead to varying clinical impacts. For instance, ChatGPT-4o demonstrated a tendency toward simplification when explaining atypical symptoms—for example, its response to the symptom “chest tightness” lacked a multietiological analysis, which could potentially mislead patients in certain contexts. In contrast, DeepSeek-v3 provided more comprehensive explanations, which contributed to greater informational completeness but might also increase cognitive load for users, particularly those with limited health literacy. Both models performed consistently in terms of safety, with no instances of inappropriate medical advice. This reliability can likely be attributed to the implementation of high-standard safety alignment techniques during model training and deployment [32].

### Language Adaptability and Application Scenarios

In terms of language adaptability, both ChatGPT-4o and DeepSeek-v3 demonstrated consistent scoring performance across both Chinese and English language settings, indicating strong cross-linguistic transfer capabilities and promising potential for global health care service delivery. This observation is consistent with previous findings by Wang et al [33], who noted that ChatGPT-4o effectively mitigates the impact of language differences on information accuracy. Such capabilities are particularly crucial for non–English-speaking patients to access accurate medical information and align with the broader movement toward equitable global medical education and health care services [34].

From an end-user application perspective, DeepSeek-v3’s strength in providing specialized therapeutic recommendations may be better suited for supporting clinical decision-making by health care professionals. In contrast, ChatGPT-4o, with its clear and concise language, appears more appropriate for delivering basic health education to patients with asthma. This distinction suggests that future AI-driven health care systems should adopt a scenario-based design tailored to users’ varying levels of health literacy and informational needs, enabling stratified service delivery. In addition, both models exhibited limitations in adequately covering key differential diagnoses—such as recurrent viral infections, cystic fibrosis, and tracheomalacia [29]—highlighting the need for further advancement in constructing a comprehensive clinical knowledge framework within LLMs.

### Domain-Specific Performance and Missing Clinical Updates

The analysis of ChatGPT-4o further reveals significant differences in the model’s performance across various aspects of asthma. It excels in structured knowledge output, scoring full marks (mean 4.00, SD 0.0) in defining asthma, its causes, and addressing COVID-19–related questions. However, its performance in clinical feature analysis is lower (3.78, SD 0.16), partly due to the omission of the influence of hormonal rhythms on symptoms. Its treatment-related score is also impacted by the failure to cover newer advancements such as anti–TSLP therapy and bronchial thermoplasty. Notably, recent trials have highlighted the benefits of formoterol as a rapid-relief medication, leading GINA to discourage the use of short-acting beta2-agonists as standalone therapy since 2022 due to the increased risk of asthma exacerbations and poor symptom control associated with their overuse [35]. Instead, GINA recommends inhaled corticosteroids–formoterol or inhaled corticosteroids–short-acting beta2-agonists as reliever treatment options [36]. However, ChatGPT-4o does not reflect these key updates, highlighting the issue of its database’s lag in incorporating recent developments.

### The Importance of Personalized, Evidence-Based Education Tools

As a chronic disease requiring long-term management, asthma treatment decisions must take into account multiple factors, including the patient’s gender, disease severity, inhaler technique, adherence, and the cost-effectiveness of medications. These considerations make treatment strategies highly individualized and complex, highlighting the need for timely, accurate, and personalized information support tools. Studies have shown that patients’ level of asthma-related knowledge is closely linked to their treatment adherence and overall health outcomes [37]. Agusala et al [38] conducted a study on an interactive asthma education program for children aged 2‐18 years and their caregivers in Ector County, Texas. The findings revealed that asthma-related education for both caregivers and children led to improved symptom management and reduced acute exacerbations, underscoring the significance of asthma education for patients. Walter et al [39] reviewed 6 intervention studies, all of which demonstrated a decrease in asthma attacks and notable enhancements in overall quality of life. Nevertheless, current patients with asthma and their caregivers often lack sufficient knowledge to effectively manage and prevent the disease, with their educational needs frequently going unmet. This limits the widespread implementation and effectiveness of educational interventions. These challenges underscore the importance of developing intelligent and highly accessible information support tools—such as language models—to bridge these gaps and enhance asthma care.

### Limitations and Future Directions

This study demonstrates the potential of ChatGPT-4o and DeepSeek-v3 as innovative tools for health education, capable of delivering clear and accurate medical information through conversational interactions. These capabilities may support patients and caregivers in making more informed treatment decisions. However, as LLMs, both systems exhibit inherent limitations that warrant critical consideration.

First, the knowledge base of LLMs is fixed at the time of their most recent update and does not reflect real-time advances in the medical field. This temporal constraint hinders the incorporation of new treatment protocols, emerging therapies, and updated clinical guidelines, thereby affecting the relevance and accuracy of generated recommendations [40].

Second, the training data for these models are primarily derived from publicly available internet sources, which may not comprehensively include high-quality medical resources such as evidence-based clinical guidelines, care pathways, textbooks, or peer-reviewed literature. Consequently, their outputs may lack sufficient grounding in evidence-based medicine, especially for complex or highly specialized clinical topics [41].

Third, the adequacy of model-generated responses is contingent upon the frequency and depth of topic representation in the training corpus. For rare diseases, novel treatments, or underrepresented conditions, both models may struggle to produce complete or satisfactory answers due to limited data exposure [40].

Fourth, the practical effectiveness of LLMs in patient-facing applications is influenced by users’ health literacy and ability to formulate clear, structured queries. Individuals without medical training may find it difficult to pose precise questions, potentially compromising the clarity and relevance of the AI’s responses. In addition, the models exhibit sensitivity to prompt phrasing, and subtle variations in user input can lead to divergent outputs.

Fifth, this study assessed only the models’ initial responses without considering the potential benefits of multiturn dialogue. In real-world applications, extended interactions may allow for clarification of vague input and generation of more comprehensive answers. Thus, single-turn evaluations may underestimate the practical value of these models in dynamic health care contexts. In multiturn conversations, the model needs to adjust its responses based on previous answers, clarify vague inputs, and provide more comprehensive advice. However, current AI models still face challenges in handling complex and extended dialogues, potentially failing to maintain consistency or provide deep enough analysis. This is particularly crucial in medical decision-making. For instance, when inquiring about medical history and symptoms, the AI needs to rely on prior inputs to form a coherent and evidence-based suggestion [42].

Finally, despite the impressive capabilities of LLMs in delivering medical information, they cannot serve as substitutes for licensed health care professionals. Medical decision-making is a complex and high-stakes process that requires not only accurate knowledge but also clinical experience, physical examination skills, ethical judgment, and contextual understanding—areas in which LLMs remain fundamentally limited. These models are incapable of performing physical assessments, interpreting nonverbal cues, or incorporating emotional and psychosocial factors into patient care [43,44]. In critical situations, such as managing acute exacerbations of chronic conditions or distinguishing between overlapping clinical syndromes, human expertise and oversight are essential. Overreliance on AI without professional validation may lead to delayed diagnoses or inappropriate treatments. Therefore, while LLMs can serve as valuable tools in health communication and patient education, their role must remain supportive rather than autonomous, with clearly defined boundaries for clinical responsibility and accountability [45].

In the future, as AI models continue to gain traction in health care settings, their potential impact will span the entire health care process, from primary health education to clinical decision support [46,47]. In practical applications, it is crucial to emphasize the role of AI as an auxiliary tool, avoiding the misconception of using it as the primary decision maker in clinical scenarios [48]. Generative AI has the potential to play a key role in enhancing patient health literacy and promoting the democratization of medical knowledge [49]. This is particularly important in areas with limited health care resources, where AI can provide patients with broader access to vital health information.

It is recommended that future intelligent health care systems be designed with a “layered response + human referral” approach, positioning AI as the “first responder” in routine management scenarios [50]. In cases of high risk or acute conditions, automatic alerts and human intervention mechanisms should be introduced to ensure both the safety and effectiveness of the service. This progressive integration of human-AI collaboration may become the key pathway for maximizing the value of AI technology in managing chronic respiratory diseases.

### Conclusion

This study systematically evaluated the performance of ChatGPT-4o and DeepSeek-v3 in providing asthma-related education to patients with asthma and found that both models have distinct strengths. DeepSeek-v3 excels in knowledge updates and clinical reasoning, covering a broader range of the latest treatment methods and complex symptom analysis. On the other hand, ChatGPT-4o is clearer and more accessible in explaining basic concepts and language. Both models provide generally accurate medical information, but there are still limitations, particularly in areas such as differential diagnosis and the completeness of treatment plans, with a noticeable lag in updates to the latest clinical guidelines.

These AI tools can serve as effective supplementary aids in providing asthma-related education to patients with asthma, helping patients better understand their condition and manage symptoms. However, they currently cannot fully replace professional medical advice, especially in complex cases or high-risk situations. Future improvements are needed in the models’ knowledge updating mechanisms and clinical reasoning capabilities, along with enhanced human-AI collaboration, to ensure patients receive health information that is both accurate and understandable. These findings are not only significant for asthma management but also provide valuable insights for the application of AI in the education of other chronic diseases.

## Supplementary material

10.2196/65365 Multimedia Appendix 1 ChatGPT-4.0’s responses to asthma-related inquiries in an English-language context.

10.2196/65365 Multimedia Appendix 2 ChatGPT-4.0’s responses to asthma-related inquiries in a Chinese-language context.

10.2196/65365 Multimedia Appendix 3 DeepSeek’s responses to asthma-related inquiries in an English-language context.

10.2196/65365 Multimedia Appendix 4 DeepSeek -3’s responses to asthma-related inquiries in a Chinese-language context.

10.2196/65365 Multimedia Appendix 5 Comparative scores of 2 artificial intelligence models across multiple evaluation dimensions in a bilingual context.

## Abbreviations

AI: artificial intelligence
API: application programming interface
GINA: Global Initiative for Asthma
LLM: large language model
RAG: retrieval-augmented generation
TSLP: thymus stromal lymphopoietin
