# Supplementary material for: Assessing the Role of Large Language Models Between ChatGPT and DeepSeek in Asthma Education for Bilingual Individuals: Comparative Study
Source: JMIR Med Inform. 2025 Aug 13;13:e65365. doi: 10.2196/65365 (PMC12349887; doi:10.2196/65365)
Supplement: Multimedia Appendix 1 [file medinform-v13-e65365-s001.doc]

**Supplementary Table 1. ChatGPT-4.0’s Responses to Asthma-Related Inquiries in an English-Language Context**

| **NO.** | Questions | **Responses** | **Consistency** | **Completeness** | **Potential Bias** | **Reasoning Ability** | **Comprehension** | **Reliability** | **Safety** |
| --- | --- | --- | --- | --- | --- | --- | --- | --- | --- |
|  | **Basic Definitions** |  |  |  |  |  |  |  |  |
| **Q1** | I am 25 years old and have been experiencing coughing and chest tightness, especially in the mornings and at night. I took amoxicillin, but it had no effect. Could this be asthma? | Your symptoms—coughing and chest tightness, especially in the mornings and at night—are common signs of asthma, particularly nocturnal asthma, where symptoms worsen at night or early morning. The fact that amoxicillin (an antibiotic) had no effect also suggests that your condition is unlikely to be caused by a bacterial infection, reinforcing the possibility of asthma or another respiratory condition.  Key Signs That Suggest Asthma  Coughing (especially at night, early morning, or after exercise) Chest tightness or discomfort Shortness of breath Wheezing (a whistling sound when breathing) Symptoms triggered by allergens, cold air, exercise, or strong smells  What You Should Do Next  Monitor Your Symptoms: Keep track of when and how often symptoms occur (e.g., after exposure to dust, exercise, or cold air).  Check for Triggers: Common asthma triggers include dust, pollen, pets, mold, strong odors, and cold air.  Try a Bronchodilator: If you have access to a short-acting beta-agonist inhaler (e.g., albuterol/salbutamol), see if it helps relieve symptoms. If it does, asthma is more likely.  Consult a Doctor:  You may need lung function tests (spirometry, peak flow test) to confirm asthma.  A trial of asthma medication (such as inhaled corticosteroids or bronchodilators) may be recommended.  Since you already have asthma (as per your medical history), your symptoms could indicate poorly controlled asthma. You may need adjustments to your treatment plan (e.g., adding a controller inhaler if you’re not using one).  Would you like help in preparing questions for your doctor or understanding potential treatment options? | 4 | 4 | 4 | 4 | 4 | 4 | 4 |
| **Q2** | Is asthma a common condition? Could I have it? | Yes, asthma is a common condition. It affects millions of people worldwide, including children and adults. Asthma is a chronic inflammatory disease of the airways that causes symptoms like wheezing, shortness of breath, chest tightness, and coughing. Triggers can include allergens (like pollen or dust mites), respiratory infections, cold air, exercise, and stress.  As for whether you have asthma, common signs include:  -Frequent coughing, especially at night or after exercise  - Wheezing (a whistling sound when breathing)  - Shortness of breath or feeling like you can’t get enough air  - Chest tightness or pain  - Symptoms worsening with certain triggers (e.g., allergens, cold air, smoke)  If you suspect you have asthma, it’s best to see a doctor for a proper evaluation. They may perform lung function tests, such as spirometry, to assess your breathing and confirm a diagnosis. Let me know if you’d like more details! | 4 | 4 | 4 | 4 | 4 | 4 | 4 |
| **Q3** | suspect I have asthma. How can I get a diagnosis? | Since you suspect you have asthma, the best way to get a diagnosis is to see a healthcare provider, such as a primary care physician or a pulmonologist. Here’s what you can expect during the diagnostic process:  1. Medical History & Symptoms Discussion  Your doctor will ask about:  Your symptoms (wheezing, shortness of breath, coughing, chest tightness).  When and how often symptoms occur (e.g., during exercise, at night, after exposure to allergens).  Any known triggers (dust, pollen, cold air, smoke, etc.).  Family history of asthma or allergies.  2. Physical Examination  Your doctor will listen to your lungs using a stethoscope to check for wheezing or other abnormalities.  3. Lung Function Tests (Spirometry or Peak Flow Test)  Spirometry: Measures how much air you can breathe in and out and how fast.  Peak Flow Test: Uses a peak flow meter to measure how well you can push air out of your lungs.  4. Bronchodilator Test  If spirometry results suggest asthma, you may be given an inhaler (bronchodilator) and asked to repeat the test to see if your lung function improves. A significant improvement suggests asthma.  5. Additional Tests (If Needed)  Methacholine Challenge Test: If your symptoms are unclear, this test checks how sensitive your airways are.  Allergy Testing: Identifies potential triggers like pollen, dust mites, or pet dander.  Fractional Exhaled Nitric Oxide (FeNO) Test: Measures airway inflammation.  Chest X-ray or CT Scan: Used to rule out other conditions like infections or structural lung issues.  Next Steps  If diagnosed, your doctor will prescribe an asthma action plan with medications (e.g., inhalers) and lifestyle recommendations.  If asthma is ruled out, they may look into other possible causes of your symptoms, such as allergies, acid reflux, or anxiety-related breathing issues.  Would you like tips on preparing for your doctor’s visit? | 4 | 4 | 4 | 4 | 4 | 4 | 4 |
| **Q4** | What tests are needed to diagnose asthma? Are they blood tests or something else? | Asthma is primarily diagnosed using **lung function tests** rather than blood tests. Here are the key tests used to diagnose asthma:  **1. Spirometry (Main Test)**  Measures how much air you can inhale and exhale and how quickly you can exhale.  You breathe into a device called a **spirometer** before and after using a bronchodilator (a medication that opens the airways).  If your lung function improves significantly after using the bronchodilator, it suggests **asthma**.  **2. Peak Flow Measurement**  A simple handheld device (**peak flow meter**) measures how fast you can blow air out of your lungs.  Lower readings may indicate **airway obstruction**, which is common in asthma.  **3. Methacholine Challenge Test (If Spirometry Is Inconclusive)**  Methacholine is inhaled to see if it causes airway narrowing.  If your lung function drops by a certain percentage, it's a sign of **asthma**.  **4. FeNO Test (Fractional Exhaled Nitric Oxide Test)**  Measures the level of **nitric oxide** in your breath, which indicates airway inflammation.  Higher levels suggest **eosinophilic (allergic) asthma**.  **5. Allergy Testing (Optional)**  Skin or blood tests check for allergic triggers (e.g., pollen, dust mites).  This is helpful if asthma symptoms are linked to **allergic reactions**.  **6. Chest X-ray or CT Scan (To Rule Out Other Conditions)**  These imaging tests help ensure that symptoms aren't due to **lung infections, COPD, or other diseases**.  **7. Blood Tests (Less Common for Asthma Diagnosis)**  While not a primary test, blood tests may check for:  **Eosinophil count** (high levels suggest allergic inflammation).  **IgE levels** (linked to allergic asthma).  **Conclusion**  Asthma is mainly diagnosed through **lung function tests** (like **spirometry**), not blood tests. However, **blood tests and allergy tests** may be done if allergic asthma is suspected. Since you have asthma, have you ever undergone spirometry or other tests? | 4 | 4 | 4 | 4 | 4 | 4 | 4 |
| **Q5** | I've been coughing, wheezing, and feeling tightness in my chest recently. Do these symptoms mean I have asthma? | Coughing, wheezing, and chest tightness are common symptoms of asthma, but they can also be caused by other conditions like allergies, respiratory infections, or even anxiety. These symptoms do not automatically mean you have asthma, but they do suggest that your airways may be irritated or inflamed.  Asthma is typically diagnosed based on:  1. Symptom history: If these symptoms occur intermittently, especially with triggers such as exercise, cold air, or allergens, it may point towards asthma.  2. physical exam: Your doctor will listen for wheezing or other signs of airflow limitation.  3. Pulmonary function tests: Spirometry or other tests may be done to measure how well your lungs are functioning and to determine if the airflow is reduced, which is common in asthma.  4. Response to medication: If symptoms improve with the use of bronchodilators (medications that open up the airways), it may indicate asthma.  It’s important to see a healthcare provider to get a proper diagnosis. They may recommend tests to confirm whether you have asthma or if another condition is causing your symptoms. If it is asthma, your doctor can help develop an appropriate treatment plan to manage your symptoms. | 4 | 4 | 4 | 4 | 4 | 4 | 4 |
| **Q6** | I have asthma. Why do doctors say it might be related to genetics? | Doctors suggest that asthma may be related to genetics because studies have shown that it often runs in families. If a close family member, such as a parent or sibling, has asthma, you are more likely to develop it.  Here’s why genetics play a role:   1. **Inherited Risk** – Certain genes influence how the immune system responds to allergens, pollution, and infections. These genes can make some people more prone to inflammation and airway sensitivity, leading to asthma. 2. **Family Patterns** – Research has found that children of parents with asthma have a higher risk of developing it, especially if both parents are affected. 3. **Immune System Genes** – Genes related to the immune system, such as those involved in allergic reactions (like IgE production), can increase the likelihood of asthma. 4. **Gene-Environment Interaction** – While genetics contribute to asthma risk, environmental factors (such as pollen, dust, smoke, and respiratory infections) can trigger symptoms, especially in genetically predisposed individuals.   Even though asthma has a genetic component, lifestyle and environmental factors also play a major role in its development and severity. Do you have family members with asthma or allergies? | 4 | 4 | 4 | 4 | 4 | 4 | 4 |
| **Q7** | The doctor said I have asthma. Why are my allergy tests negative? | Asthma and allergies are related but not the same. You can have asthma without allergies, and negative allergy tests do not rule out asthma. Here are some possible explanations:   1. **Non-Allergic Asthma** – Not all asthma is triggered by allergens. Irritants like smoke, pollution, strong odors, cold air, stress, or exercise can trigger asthma symptoms. 2. **Sensitivity to Non-Allergic Triggers** – You may be reacting to weather changes, respiratory infections, or other non-allergic factors. 3. **False Negative Allergy Test** – Allergy tests (skin or blood tests) are not perfect. They might miss certain allergens or not detect delayed allergic reactions. 4. **Other Underlying Conditions** – Conditions like intrinsic asthma (which is not allergy-driven) or airway hyperreactivity (where airways overreact to stimuli) can cause asthma-like symptoms without allergies.   Your doctor may recommend further tests, like spirometry or a methacholine challenge test, to confirm the type and severity of your asthma. If you notice triggers that worsen your symptoms, keeping a symptom diary can help identify patterns. Let me know if you need help understanding your diagnosis! | 4 | 4 | 4 | 4 | 4 | 4 | 4 |
|  | **Clinical characteristics** |  |  |  |  |  |  |  |  |
| **Q1** | What are the typical symptoms of asthma? How can I tell if I have it? | Asthma symptoms can vary from person to person, but the most common ones include:  **Typical Symptoms of Asthma:**   1. **Shortness of Breath** – Feeling out of breath, especially during physical activity or at night. 2. **Wheezing** – A whistling or squeaky sound when breathing, especially when exhaling. 3. **Coughing** – Persistent cough, often worse at night or early in the morning. 4. **Chest Tightness or Pain** – A feeling of pressure or discomfort in the chest. 5. **Increased Mucus Production** – The airways may produce extra mucus, leading to congestion.   **How to Tell If You Have Asthma:**  You may have asthma if you experience these symptoms regularly, especially if they:  Worsen with exercise, cold air, or allergens (e.g., dust, pollen, smoke).  Occur more at night or early morning.  Improve with asthma medications (such as inhalers).  **What to Do Next?**  Since you already have asthma and take daily medication, it’s important to monitor your symptoms. If you notice worsening symptoms, more frequent attacks, or reduced effectiveness of your medication, you may need to adjust your treatment plan with your doctor. Keeping a symptom diary and using a peak flow meter can help track your asthma control.  Would you like guidance on how to manage triggers or improve symptom control? | 4 | 4 | 4 | 4 | 4 | 4 | 4 |
| **Q2** | Besides coughing and wheezing, are there other less common symptoms of asthma? | Yes, asthma can have several less common symptoms, in addition to coughing and wheezing. Some of these include:   1. **Shortness of breath**: Difficulty breathing or feeling out of breath, especially during physical activity or at night. 2. **Chest tightness**: A sensation of pressure or constriction in the chest, which may feel like something is heavy on your chest. 3. **Fatigue**: Feeling unusually tired or weak, especially after exertion, due to reduced airflow. 4. **Sleep disturbances**: Asthma symptoms, like coughing or shortness of breath, may worsen at night, leading to poor sleep. 5. **Frequent need to clear your throat**: A persistent urge to clear your throat due to mucus or irritation. 6. **Pale or bluish lips or face**: This can occur if asthma symptoms are severe and lead to inadequate oxygen levels in the blood. 7. **Difficulty talking or performing daily activities**: When asthma symptoms flare up, it may be hard to speak in full sentences or carry out normal tasks due to breathlessness.   Asthma symptoms can vary from person to person, so it's important to recognize all the signs to better manage the condition. | 4 | 4 | 4 | 4 | 4 | 4 | 4 |
| **Q3** | Can asthma cause other physical problems, like headaches or loss of appetite? | Yes, asthma can contribute to other physical issues, including headaches and loss of appetite, although they are not direct symptoms of asthma itself. Here’s how asthma may be linked to these problems:   1. **Headaches**: Asthma attacks or poorly controlled asthma can cause tension and strain, leading to headaches. These headaches may occur due to the body’s stress response during an asthma flare-up or from the side effects of certain medications (e.g., bronchodilators). 2. **Loss of Appetite**: Asthma medications, particularly oral steroids, can sometimes cause changes in appetite. Additionally, severe asthma attacks or difficulty breathing can make eating uncomfortable, leading to reduced appetite. In some cases, the body’s stress response during an asthma flare may also affect appetite.   If these symptoms are persistent or severe, it’s important to discuss them with your doctor to ensure that your asthma management plan is optimal and to rule out other potential causes. | 4 | 4 | 4 | 4 | 4 | 4 | 4 |
| **Q4** | What would an asthma attack look like? What should I do during an attack? | An asthma attack typically involves the following symptoms:   1. **Wheezing**: A high-pitched whistling sound while breathing, especially during exhalation. 2. **Shortness of breath**: Feeling like you can’t catch your breath or like you're suffocating. 3. **Coughing**: Frequent coughing, especially at night or early morning. 4. **Chest tightness**: A feeling of pressure or constriction in the chest. 5. **Increased mucus production**: You might cough up more mucus than usual.   **What to do during an asthma attack:**   1. **Stay calm**: Try to keep calm, as panic can make symptoms worse. 2. **Use your inhaler**: If you have a rescue inhaler (e.g., albuterol), use it as prescribed. Follow the correct technique to ensure proper delivery. 3. **Sit up**: Avoid lying down, as it can make breathing more difficult. Sitting upright can help open up your airways. 4. **Focus on slow, controlled breathing**: Inhale slowly through your nose, hold for a few seconds, and then exhale slowly through your mouth. 5. **Seek help**: If symptoms don’t improve after using your inhaler, or if they worsen, seek emergency medical help immediately.   If you experience an asthma attack regularly, it’s important to have a management plan in place, which may involve daily medication and regular check-ups with your doctor. | 4 | 3 | 4 | 4 | 4 | 4 | 4 |
| **Q5** | Why do I have no issues during the day, but start coughing and feeling tight at night? Is this characteristic of asthma? | Yes, it is quite characteristic of asthma to have symptoms worsen at night, a phenomenon known as "nocturnal asthma." This happens for a few reasons:   1. **Airway cooling**: At night, your body temperature drops, which can cause the airways to constrict, triggering coughing and wheezing. 2. **Posture**: Lying down can increase mucus production and make it harder for your lungs to clear it, leading to coughing and tightness. 3. **Increased airway sensitivity**: Asthma tends to be more active at night, with heightened airway sensitivity to allergens, irritants, and even changes in hormone levels. 4. **Relief from daytime activity**: During the day, physical activity and movement help keep the airways more open. At night, when you're at rest, the lack of movement may make it easier for asthma symptoms to surface.   If this is a common pattern for you, it could be worth discussing with your healthcare provider to ensure you're managing your asthma effectively, especially at night. | 3 | 3 | 4 | 4 | 4 | 4 | 4 |
| **Q6** | I often feel tightness in my chest. Could this be a sign of an asthma attack? | Yes, chest tightness can be a sign of an asthma flare-up or an impending asthma attack. Since you have asthma, it's important to monitor your symptoms closely. Other signs of an asthma attack include:  **Shortness of breath** (especially if it worsens or doesn’t improve with medication) **Wheezing** or a whistling sound while breathing **Coughing** (especially at night or after exercise) **Increased mucus production** **Difficulty speaking** due to breathlessness  **What to Do:**   1. **Use Your Rescue Inhaler** – If you have an albuterol (or similar) inhaler, use it as prescribed. If the tightness doesn’t improve after using it, that’s a warning sign. 2. **Monitor Symptoms** – If your symptoms worsen or you struggle to breathe, seek **immediate medical help.** 3. **Avoid Triggers** – Identify and avoid common triggers like allergens, smoke, cold air, or stress. 4. **Track Peak Flow (if available)** – If you use a peak flow meter, a low reading can indicate worsening asthma.   If you frequently experience chest tightness, it might mean your asthma isn’t well-controlled. You may need a medication adjustment, so consider consulting your doctor. Would you like advice on improving asthma control? | 3 | 3 | 3 | 4 | 3 | 3 | 3 |
|  | **Treatment** |  |  |  |  |  |  |  |  |
| **Q1** | I suspect I have asthma. Can I use any medications to relieve the symptoms before seeing a doctor? | If you're experiencing asthma-like symptoms, such as wheezing, shortness of breath, or a persistent cough, it’s important to seek medical advice as soon as possible. Self-diagnosing and using medication without a doctor’s guidance may not be effective or safe.  In the meantime, if you're unsure and need some relief, here are a few options that could help, but they should only be used as a temporary measure:   1. **Inhalers (Salbutamol or Albuterol)**: These are commonly used as rescue medications to quickly relieve shortness of breath and wheezing. They work by opening up the airways. However, using this type of medication without a proper diagnosis may mask the symptoms. 2. **Avoiding Triggers**: If you can identify what might be triggering your symptoms (such as dust, smoke, or cold air), it’s best to avoid those triggers until you get a diagnosis.   Remember, these are just temporary measures, and seeing a doctor is essential for determining whether you have asthma and for receiving appropriate treatment.  If you are already an asthma patient and have medication prescribed, make sure to follow your treatment plan and consult your doctor if symptoms worsen. | 4 | 4 | 4 | 4 | 4 | 4 | 4 |
| **Q2** | I’ve been diagnosed with asthma. What medication should I take? | I'm sorry to hear about your asthma diagnosis. The medications prescribed for asthma can vary depending on the severity of your condition, but generally, there are two types of medications used to manage asthma:   1. **Quick-relief (rescue) medications**: These are fast-acting medications used to relieve symptoms during an asthma attack. Common options include:    - **Short-acting beta-agonists (SABAs)**: Such as albuterol or levalbuterol.    - **Anticholinergics**: Like ipratropium. 2. **Long-term control medications**: These are used to prevent asthma symptoms over time, by reducing inflammation and helping to keep the airways open. Common options include:   **Inhaled corticosteroids**: Such as fluticasone, budesonide, or beclometasone.  **Long-acting beta-agonists (LABAs)**: Like salmeterol.  **Leukotriene modifiers**: Like montelukast.  **Biologics**: For severe asthma, drugs like omalizumab or mepolizumab may be prescribed.  Your doctor will recommend the appropriate medications based on the frequency of your asthma symptoms, any triggers, and how well your asthma is controlled. Be sure to follow your healthcare provider’s advice and use medications as prescribed. | 4 | 4 | 4 | 4 | 4 | 4 | 4 |
| **Q3** | The doctor prescribed me budesonide. Can you tell me what side effects I might experience? | Budesonide is a corticosteroid commonly prescribed to manage asthma and other respiratory conditions. While it is generally well tolerated, there are some potential side effects that you might experience, especially if used over a long period. These may include:   1. **Local side effects (inhaled form)**:   Hoarseness or a sore throat  Oral thrush (a fungal infection in the mouth)  Coughing or irritation in the throat   1. **Systemic side effects (rare but possible)**:   Increased risk of infections  Headache  Nausea  Weight gain  Increased blood sugar levels (which could be a concern for people with diabetes)  Mood changes, such as anxiety or depression  Osteoporosis (long-term use)  To reduce the risk of local side effects, it’s recommended to rinse your mouth after using the inhaler. Always follow your doctor’s instructions for usage, and report any side effects you experience. If you’re concerned about long-term effects, you can discuss alternative options with your doctor. | 4 | 4 | 4 | 4 | 4 | 4 | 4 |
| **Q4** | After using budesonide-formoterol, my heart rate increased, and I felt uncomfortable. Is this a normal reaction? | An increase in heart rate and feeling uncomfortable can occur as a side effect of using budesonide-formoterol, as the formoterol component is a long-acting beta agonist (LABA), which can stimulate the heart and cause symptoms like palpitations or increased heart rate in some individuals.  While this reaction isn't uncommon, it may not be normal for everyone. If you experience significant discomfort, persistent palpitations, or other concerning symptoms, it's important to consult your healthcare provider. They can assess your symptoms and determine whether you should adjust your medication or explore alternatives. | 4 | 4 | 4 | 4 | 4 | 4 | 4 |
| **Q5** | My father also has asthma. After using salmeterol/fluticasone, he had a sore throat and hoarseness. Are these side effects of the medication? | Yes, sore throat and hoarseness can be side effects of using **salmeterol/fluticasone**. This combination medication is a long-acting beta-agonist (LABA) and a corticosteroid, and while it helps manage asthma by reducing inflammation and opening the airways, it can cause side effects like:  **Sore throat**: This is a common side effect, as inhaled corticosteroids can irritate the throat.  **Hoarseness**: This can occur because the inhaled corticosteroid can cause irritation to the vocal cords and surrounding tissues.  To reduce the risk of these side effects, it’s helpful to rinse the mouth and throat with water after each use of the inhaler. If your father continues to experience these symptoms, it’s advisable to speak with his doctor, as they may recommend an alternative medication or further adjustments to the treatment plan. | 4 | 4 | 4 | 4 | 4 | 4 | 4 |
| **Q6** | Are there any new treatments that can help reduce frequent asthma attacks? | A promising new treatment for reducing frequent asthma attacks is Benralizumab, a monoclonal antibody that targets eosinophils, white blood cells involved in inflammation. Recent studies have shown that Benralizumab, when administered during an asthma flare-up, can significantly improve outcomes compared to the traditional treatment with steroids like prednisolone. It reduces the risk of treatment failure, including the need for additional medication, hospital visits, or even death, by a substantial margin.  In trials, those receiving Benralizumab alone experienced fewer treatment failures and improved symptom recovery, with fewer side effects than those using steroids. It is particularly beneficial because it does not carry the severe side effects associated with steroids, such as high blood sugar, which makes it a safer option for long-term or frequent flare-up management ([A Breakthrough in Asthma Treatment: A New Hope for Millions - Industry Tap](https://www.industrytap.com/a-breakthrough-in-asthma-treatment-a-new-hope-for-millions/75915)) ([First New Treatment in 50 Years for Asthma Attacks and COPD ‘Could be a Game-Changer’](https://www.goodnewsnetwork.org/first-new-treatment-in-50-years-for-asthma-attacks-and-copd-called-game-changer/)) ([New Drug For Asthma And COPD Is The First Found in 50 Years : ScienceAlert](https://www.sciencealert.com/new-drug-for-asthma-and-copd-is-the-first-found-in-50-years)).  This breakthrough could mark the first significant advancement in asthma treatment in over 50 years. While the drug is already used for long-term asthma management, its application during exacerbations, as tested in these trials, is still being explored in larger, phase 3 trials ([First new treatment for asthma attacks in 50 years](https://medicalxpress.com/news/2024-11-treatment-asthma-years.pdf)) ([New Drug For Asthma And COPD Is The First Found in 50 Years : ScienceAlert](https://www.sciencealert.com/new-drug-for-asthma-and-copd-is-the-first-found-in-50-years)). | 3 | 2 | 3 | 3 | 3 | 3 | 3 |
| **Q7** | What’s the difference between inhaled and oral medications for asthma? Which is more effective? | Inhaled and oral asthma medications both help control asthma symptoms, but they differ in how they are delivered and absorbed in the body. Here’s a breakdown:  **Inhaled Medications:**  **Delivery:** Inhaled medications go directly to the lungs, where they are needed. They are typically administered via inhalers or nebulizers.  **Types:** There are two main types of inhaled asthma medications:  **Relievers (Short-acting bronchodilators):** They work quickly to open the airways during an asthma attack (e.g., albuterol).  **Controllers (Long-acting bronchodilators or corticosteroids):** They help reduce inflammation and prevent symptoms over time (e.g., fluticasone, salmeterol).  **Effectiveness:** Because the medication is directly delivered to the lungs, it acts faster and requires lower doses to be effective. It also minimizes systemic side effects.  **Oral Medications:**  **Delivery:** Oral medications are taken by mouth and need to be absorbed into the bloodstream before reaching the lungs, which means they take longer to act.  **Types:** These include corticosteroids (like prednisone), leukotriene modifiers (e.g., montelukast), and theophylline.  **Effectiveness:** Oral medications can be very effective, especially for long-term control or when asthma is not well-controlled with inhalers alone. However, they may come with more significant side effects, such as weight gain, mood changes, or bone thinning (especially with long-term use of corticosteroids).  **Which is more effective?**  **Inhaled medications** are generally considered more effective for asthma management because they target the lungs directly, offering faster relief and fewer side effects.  **Oral medications** are often used when inhaled treatments aren’t sufficient or when inflammation is more widespread.  Inhaled medications are the preferred first-line treatment for most asthma patients due to their targeted action and lower risk of systemic side effects. However, a combination of both may be needed depending on the severity of the asthma. Always consult your healthcare provider to determine the best treatment plan for you. | 4 | 4 | 4 | 4 | 4 | 4 | 4 |
| **Q8** | I’m using asthma medication, but I forgot to take it today. What should I do to make up for it? | If you've forgotten to take your asthma medication today, here’s what you can do:   1. **If it's a long-term control medication** (e.g., inhaled corticosteroids, leukotriene modifiers), it's important to take it as soon as you remember, even if it's later in the day. Do not double the dose to make up for the missed one, as this could increase the risk of side effects. 2. **If it's a rescue inhaler** (e.g., albuterol), take it only if you are experiencing symptoms like wheezing, shortness of breath, or chest tightness. It’s typically used on an as-needed basis rather than regularly. 3. **Monitor your symptoms**: If you feel any signs of worsening asthma, like increased shortness of breath or wheezing, contact your doctor for guidance.   For future prevention, setting a reminder on your phone or in a daily planner can help you stay on track with your medication schedule. | 4 | 4 | 4 | 4 | 4 | 4 | 4 |
| **Q9** | If I don't want to rely on medication forever, can I manage asthma with diet or exercise? | While asthma is a chronic condition, it's possible to manage it with a combination of lifestyle changes, including diet and exercise, along with medical treatments. While diet and exercise can't replace medications entirely, they can help reduce symptoms and improve overall health. Here's how:  **Diet**  **Anti-inflammatory foods**: Eating foods that reduce inflammation, such as fruits (berries, oranges), vegetables (leafy greens, bell peppers), and fatty fish (salmon, mackerel), may help lower asthma symptoms.  **Magnesium-rich foods**: Magnesium can help with bronchial relaxation. Foods like spinach, almonds, and avocados are good sources.  **Avoid triggers**: Some people find that certain foods, like dairy or processed foods, can worsen asthma symptoms. Keep track of your triggers and avoid them when possible.  **Exercise**  **Regular physical activity**: Aerobic exercise (like walking, swimming, or cycling) can improve lung function and overall stamina. However, you should consult your doctor about which exercises are safest for you, especially if you're prone to exercise-induced asthma.  **Breathing exercises**: Techniques like pursed-lip breathing or diaphragmatic breathing can help control shortness of breath and improve lung function.  **Strength training**: Building muscle can help support better posture and improve respiratory function.  Always consult with your doctor before making any major changes to your asthma management, especially if you're considering reducing medication. They can help you create a balanced approach that works for your specific condition. | 4 | 4 | 4 | 4 | 4 | 4 | 4 |
| **Q10** | The doctor prescribed inhaled steroids after diagnosing asthma. Will I become dependent on them in the future? | Inhaled steroids are commonly prescribed to manage asthma and help reduce inflammation in the airways. They are considered a long-term treatment and are generally safe when used as directed. The goal of inhaled steroids is to control asthma symptoms, reduce flare-ups, and prevent long-term damage to the lungs.  Inhaled steroids are not typically associated with dependency like other types of medications, such as certain painkillers. However, they need to be used consistently as prescribed to be effective. Stopping the medication suddenly without medical guidance can lead to worsened asthma symptoms.  If you have concerns about long-term use or side effects, it's important to discuss them with your doctor. They can monitor your progress and adjust the dosage if necessary, sometimes reducing it over time as your asthma improves. | 4 | 4 | 4 | 4 | 4 | 4 | 4 |
|  | **Assessment and management** |  |  |  |  |  |  |  |  |
| **Q1** | Will asthma affect my life expectancy? How can I manage my condition to avoid serious consequences? | Asthma, when properly managed, generally doesn't have a major impact on life expectancy. However, if asthma is uncontrolled or severe, it can lead to complications that might affect long-term health. For example, chronic asthma can increase the risk of respiratory infections, damage the lungs, and in rare cases, lead to asthma-related deaths due to severe attacks.  To manage asthma effectively and minimize serious consequences, here are some tips:   1. **Follow your treatment plan**: Take your prescribed medications (inhalers, steroids, etc.) as directed by your healthcare provider. These medications help control inflammation and prevent symptoms. 2. **Monitor symptoms**: Keep track of your asthma symptoms (coughing, wheezing, shortness of breath). This helps identify when your asthma is worsening so you can take action early. 3. **Avoid triggers**: Identify and avoid asthma triggers like allergens (dust, pollen, pet dander), smoke, strong odors, and cold air. Minimizing exposure can help reduce flare-ups. 4. **Stay active**: Regular physical activity can improve lung function and overall health. Just be sure to warm up properly and keep medication handy in case of a flare-up during exercise. 5. **Manage allergies**: If you have allergies, controlling them can reduce asthma symptoms. Antihistamines or allergy shots may help. 6. **Vaccinations**: Stay up to date on vaccines, especially the flu shot and pneumonia vaccines, to reduce your risk of infections that could worsen asthma. 7. **Regular check-ups**: Visit your doctor regularly to adjust your treatment plan if needed and to ensure you're managing the condition effectively.   By staying on top of your asthma and making lifestyle adjustments, you can minimize the risk of complications and lead a healthy, active life. | 4 | 4 | 4 | 4 | 4 | 4 | 4 |
| **Q2** | I have asthma but still smoke. Will this worsen my condition? | Yes, smoking can significantly worsen your asthma. It irritates the airways and makes it harder to breathe, which can trigger asthma attacks or exacerbate symptoms. Smoking also damages the lungs over time, increasing the risk of respiratory infections, chronic bronchitis, and other lung diseases.  Even though you are managing asthma with medication, smoking reduces the effectiveness of your treatments and increases inflammation in your airways, making your asthma harder to control. Quitting smoking can help improve your lung health and reduce asthma symptoms. If you need support in quitting, there are resources and programs that can assist you in the process. | 4 | 4 | 4 | 4 | 4 | 4 | 4 |
| **Q3** | What symptoms should I watch out for during an asthma attack, and when should I go to the hospital for treatment? | During an asthma attack, the symptoms can range from mild to severe. It's important to recognize them early so you can manage the situation effectively. Common symptoms to watch out for include:   1. **Shortness of breath**: Difficulty breathing or a feeling of tightness in the chest. 2. **Wheezing**: A whistling sound when you breathe, especially when exhaling. 3. **Coughing**: Persistent coughing, especially at night or early morning. 4. **Increased mucus production**: Thick, sticky mucus in the airways. 5. **Chest tightness**: A feeling of pressure or tightness in the chest. 6. **Difficulty talking**: Struggling to finish sentences or speak in full sentences due to shortness of breath. 7. **Fatigue**: Feeling unusually tired due to difficulty breathing.   You should seek immediate medical attention (go to the hospital) if:  **Severe wheezing or shortness of breath** that doesn't improve with your usual medication.  **Inability to speak full sentences** due to shortness of breath.  **Use of accessory muscles** to breathe (you might see your neck or chest muscles contracting more than usual).  **Lips or face turning blue** (sign of low oxygen levels).  **Not responding to inhaler** or other rescue medications.  **Symptoms worsening** despite taking your medication.  It’s important to have an asthma action plan in place and follow it if you experience these symptoms. If you're ever uncertain about the severity of an attack, it's better to err on the side of caution and seek medical help right away. | 4 | 4 | 4 | 4 | 4 | 4 | 4 |
| **Q4** | If I have asthma, how can I assess the severity of my condition? Are there self-assessment methods? | Yes, there are several self-assessment methods that can help you monitor the severity of your asthma. While a healthcare professional should provide a comprehensive evaluation, these methods can help you track your symptoms and manage your condition effectively. Here are some common ways to assess asthma severity:  **1. Symptom Tracking**  **Frequency of Symptoms**: Track how often you experience symptoms like wheezing, coughing, shortness of breath, and chest tightness. Frequent symptoms may indicate poorly controlled asthma.  **Nighttime Symptoms**: Asthma that causes waking during the night or early morning is a sign that your asthma may not be well-controlled.  **2. Peak Flow Monitoring**  A **peak flow meter** is a small device that measures how fast you can exhale air. Your healthcare provider will give you a personal best peak flow value. If your peak flow drops below 80% of this value, it could indicate worsening asthma, and you may need to adjust your treatment.  **3. Asthma Action Plan**  Follow an **asthma action plan** developed with your healthcare provider. This plan includes personalized steps based on symptom severity and peak flow measurements. It helps guide treatment adjustments, including when to use rescue medications or seek medical help.  **4. Medication Usage**  **Rescue Inhaler Use**: Frequent use of your rescue inhaler (e.g., albuterol) may suggest worsening asthma or insufficient control. You should only need to use your rescue inhaler occasionally (not more than twice a week).  **Daily Medication**: If you need to increase the dosage of your daily asthma medication or add more frequent doses, it may indicate that your asthma is getting worse.  **5. Activity Limitation**  Assess whether asthma is limiting your physical activity, such as exercise or even daily tasks. If you're unable to engage in activities without feeling shortness of breath or fatigue, your asthma may need to be reassessed.  **6. Exhalation Sounds**  Listening for wheezing, coughing, or a tight chest can help you recognize worsening asthma. If these symptoms worsen or don't improve with treatment, you may need to consult your doctor.  If you notice worsening symptoms, it’s essential to reach out to your healthcare provider for a more thorough evaluation and adjustment of your treatment plan. | 4 | 4 | 4 | 4 | 4 | 4 | 4 |
| **Q5** | I’m an asthma patient, and I take medication daily. Once my condition stabilizes, can I stop taking it? | It's important not to stop your asthma medication without consulting your doctor. Even if your asthma symptoms are under control, stopping medication could lead to a relapse or worsening of the condition. Your doctor can evaluate your asthma and advise if any adjustments to your medication are appropriate, including whether you can reduce or stop it based on your current health status. Always follow your healthcare provider's guidance when managing asthma treatment. | 4 | 4 | 4 | 4 | 4 | 4 | 4 |
| **Q6** | How can I use a peak flow meter to monitor my asthma symptoms? When is it most effective to use it? | A peak flow meter is a helpful tool for monitoring asthma symptoms and detecting early signs of worsening asthma. Here's how you can use it effectively:  **How to Use a Peak Flow Meter:**   1. **Set it up:**   Make sure the peak flow meter is clean and in good working condition.  Stand or sit up straight for an accurate reading.   1. **Take a deep breath:**   Take a deep breath in, filling your lungs completely.   1. **Blow out forcefully:**   Place the mouthpiece of the peak flow meter in your mouth.  Blow out as hard and fast as possible (not slowly). This measures how fast you can exhale.   1. **Read the result:**   Record the highest number that appears on the meter after blowing out. This is your peak flow reading.  Repeat the test 2–3 times and use the highest reading for accuracy.   1. **Track your readings:**   Keep a log of your peak flow readings over time. Compare them with your personal best peak flow (the highest reading you've ever recorded when you're well) to help assess how your asthma is doing.  **When is it Most Effective to Use It?**  **Morning and Evening:** Take readings twice a day (usually once in the morning before taking your medication and once in the evening) to track changes in your lung function.  **During Asthma Flare-ups:** If you’re experiencing symptoms like coughing, wheezing, or shortness of breath, check your peak flow more often to detect any decline in lung function.  **Before and After Medication:** Measure your peak flow before using your inhaler or medication, and then take another reading 15-20 minutes after using it. This helps assess how well your medication is working.  **Interpreting Peak Flow Readings:**  **Green Zone (80–100% of your personal best):** Your asthma is under control, and you’re not experiencing significant symptoms.  **Yellow Zone (50–80% of your personal best):** Your asthma is worsening, and you may need to use your rescue medication or adjust your treatment.  **Red Zone (below 50% of your personal best):** You are experiencing a severe asthma flare-up, and you need to take immediate action (use a rescue inhaler or seek medical attention).  By regularly monitoring with a peak flow meter, you can detect changes in your asthma early and take preventive measures before symptoms worsen. | 4 | 4 | 4 | 4 | 4 | 4 | 4 |
| **Q7** | What factors affect the long-term outlook of asthma, and how can I avoid them? | The long-term outlook of asthma can be influenced by several factors, and taking steps to manage these factors can help prevent complications. Key factors that affect asthma outcomes include:  **1. Severity of Asthma**  **Impact**: Severe asthma can result in frequent flare-ups, reduced lung function, and increased risk of complications.  **How to Manage**: Adhering to your prescribed medication regimen (e.g., inhalers, oral medications) and working with your healthcare provider to adjust treatment based on symptoms is crucial.  **2. Environmental Triggers**  **Impact**: Exposure to allergens, air pollution, smoke, and other irritants can trigger asthma symptoms and exacerbate the condition.  **How to Manage**:   - - Avoid known allergens (e.g., dust mites, pet dander, pollen).   - Limit exposure to smoke (cigarette or environmental).   - Keep your home environment clean, use air purifiers, and avoid outdoor activities during high pollution days.   **3. Allergies**  **Impact**: Allergies, particularly to pollen, mold, or pet dander, can worsen asthma symptoms.  **How to Manage**: Consider allergy testing and medications such as antihistamines or immunotherapy (allergy shots) to reduce the effect of allergens.  **4. Infections**  **Impact**: Respiratory infections like the flu or colds can make asthma worse by inflaming the airways.  **How to Manage**: Get vaccinated against flu and pneumonia, practice good hygiene (e.g., handwashing), and avoid close contact with people who are sick.  **5. Physical Activity**  **Impact**: Exercise-induced bronchoconstriction (EIB) can lead to asthma symptoms during physical exertion.  **How to Manage**: Use a bronchodilator before exercising (if recommended by your doctor) and choose exercises that don't overly strain the lungs, such as swimming.  **6. Obesity**  **Impact**: Being overweight or obese can increase the frequency and severity of asthma symptoms.  **How to Manage**: Maintain a healthy weight through diet and regular physical activity.  **7. Medication Adherence**  **Impact**: Non-adherence to prescribed asthma medications can lead to poorly controlled asthma, increased flare-ups, and long-term lung damage.  **How to Manage**: Follow your treatment plan closely and use a medication reminder if necessary. Regular check-ups with your healthcare provider will help assess if adjustments are needed.  **8. Mental Health**  **Impact**: Stress, anxiety, and depression can worsen asthma symptoms, as emotional triggers may lead to flare-ups.  **How to Manage**: Practice stress reduction techniques such as deep breathing, mindfulness, or yoga. Seek support from a mental health professional if needed.  By addressing these factors with your healthcare provider, you can optimize your asthma management and improve long-term health outcomes. | 4 | 4 | 4 | 4 | 4 | 4 | 4 |
| **Q8** | I’ve had asthma for two years. I no longer have wheezing, but I still feel short of breath after physical activity. What should I do? | It’s great that your wheezing has improved! However, shortness of breath after physical activity can still be a sign that your asthma isn’t fully controlled, or it may indicate that you're pushing your body harder than usual. Here are some steps you can take:   1. **Monitor your asthma control**: Keep track of your symptoms, peak flow readings (if you use a peak flow meter), and any triggers you might encounter during or after physical activity. 2. **Check your inhaler technique**: If you’re using a rescue inhaler (like albuterol), ensure you’re using it properly to get the maximum benefit. Sometimes, technique can impact effectiveness. 3. **Use a pre-exercise inhaler**: Some people benefit from using a preventive inhaler (like a corticosteroid or bronchodilator) before exercising. Check with your doctor to see if this might be appropriate for you. 4. **Warm up and cool down**: Gradually increasing and decreasing your exercise intensity can help minimize shortness of breath. 5. **Pace yourself**: It might help to adjust your activity levels to avoid pushing yourself too hard. Start with lower-intensity exercise and gradually increase it as your body adapts. 6. **Consider an asthma action plan**: If you don’t already have one, your doctor may suggest a personalized action plan to address physical activity and other triggers.   If this issue persists or worsens, or if you experience additional symptoms, it’s important to follow up with your healthcare provider to reassess your asthma management plan. | 4 | 4 | 4 | 4 | 4 | 4 | 4 |
| **Q8** | Can asthma be completely cured? Will I be able to live a normal life afterward? | Asthma currently does not have a complete cure, but it can be effectively managed with the right treatment plan. Many people with asthma live normal, active lives by following their prescribed medication regimen, avoiding triggers, and monitoring their symptoms. With proper management, asthma can be controlled, and flare-ups can be minimized.  While asthma may not go away, its symptoms can be reduced to the point where they don't interfere significantly with daily activities. Working with a healthcare provider to create a tailored asthma action plan is key to achieving long-term control and maintaining a good quality of life. | 4 | 4 | 4 | 4 | 4 | 4 | 4 |
| **Q10** | I have asthma, and my condition is well-controlled, but why haven’t I seen an overall improvement in my health? | While asthma can be well-controlled with medication, there are several factors that could influence whether you notice an overall improvement in your health. Here are a few possibilities:   1. **Underlying Health Issues**: Asthma can coexist with other conditions like allergies, sinus problems, or even stress, which might impact your overall health. If these aren't managed, they can make it harder to feel a complete improvement. 2. **Medication Adjustment**: Even if your asthma is well-controlled, the medications you're taking might not be the most suitable for your overall health. Sometimes, the doses or types of medication need adjustment to address other concerns like fatigue or inflammation. 3. **Diet and Lifestyle**: Factors like poor diet, lack of exercise, or inadequate sleep can hinder overall health improvement. Even if asthma is under control, these factors can prevent you from feeling your best. 4. **Environmental Triggers**: While your asthma might be controlled, exposure to environmental triggers like pollution, allergens, or extreme weather could still affect your general well-being. 5. **Mental Health**: Chronic conditions can take a toll on mental health, leading to fatigue, stress, or depression, which can make it feel like your physical health isn't improving even though you're managing your asthma well. 6. **Time and Gradual Changes**: Improvement in overall health may take time. Asthma control doesn't always translate immediately to a feeling of improved energy or wellness.   If you feel like there is no improvement despite good asthma control, it could be helpful to review your health with a doctor to ensure there aren't other factors affecting your well-being. | 4 | 4 | 4 | 4 | 4 | 4 | 4 |
| **Q11** | The doctor diagnosed me with asthma. If my symptoms don’t improve after using medication, how can I tell if I need to seek further medical attention? | If your asthma symptoms don't improve after using medication, it is important to monitor your symptoms and seek further medical attention if:   1. **Increased shortness of breath**: If you continue to experience difficulty breathing despite medication, or if your shortness of breath worsens, it's important to get checked. 2. **Frequent or worsening symptoms**: If your symptoms, like coughing, wheezing, or tightness in the chest, become more frequent or severe, it could indicate that your asthma is not well controlled. 3. **No relief from rescue inhaler**: If your rescue inhaler (fast-acting bronchodilator) doesn't provide relief or if you need to use it more than usual, it may suggest that your asthma isn't being managed effectively. 4. **Blue lips or face**: This could indicate a lack of oxygen and requires immediate medical attention. 5. **Feeling unusually fatigued**: If you're feeling much more tired than usual or unable to perform normal activities due to breathing issues, it may be a sign that your asthma is worsening. 6. **Difficulty speaking**: If you have trouble speaking in full sentences or get too winded to talk, it's a sign you need medical attention right away.   If any of these occur, contact your healthcare provider or go to the emergency room. You may need an adjustment in your medication or a more intensive treatment plan. | 4 | 4 | 4 | 4 | 4 | 4 | 4 |
|  | **Differential diagnosis** |  |  |  |  |  |  |  |  |
| **Q1** | Besides asthma, what other conditions could cause coughing and wheezing? | Coughing and wheezing can be caused by various conditions besides asthma. Some of the common ones include:   1. **Allergic Rhinitis**: Allergies can lead to inflammation in the airways, causing symptoms like coughing, wheezing, and nasal congestion. 2. **Chronic Obstructive Pulmonary Disease (COPD)**: This includes chronic bronchitis and emphysema, which can cause persistent cough and wheezing, often related to smoking or long-term exposure to irritants. 3. **Bronchitis**: Inflammation of the bronchial tubes, often due to a viral or bacterial infection, can result in coughing and wheezing. 4. **Gastroesophageal Reflux Disease (GERD)**: Acid reflux can irritate the airways and cause coughing, wheezing, and other respiratory symptoms, particularly at night. 5. **Respiratory Infections**: Viral infections like the common cold or flu can cause coughing, wheezing, and shortness of breath as a result of inflammation and mucus production. 6. **Pulmonary Embolism**: A blood clot in the lungs can cause sudden coughing, wheezing, chest pain, and shortness of breath, often accompanied by other symptoms like rapid heartbeat. 7. **Interstitial Lung Disease**: This is a group of diseases that cause scarring of lung tissue and can lead to coughing and difficulty breathing. 8. **Heart Failure**: In some cases, heart failure can lead to fluid buildup in the lungs, causing wheezing and coughing, especially when lying down. 9. **Bronchiectasis**: A condition where the airways are permanently dilated and damaged, leading to chronic coughing, wheezing, and mucus production.   If you experience persistent coughing or wheezing, it’s important to consult a healthcare professional for an accurate diagnosis. | 4 | 4 | 4 | 4 | 4 | 4 | 4 |
| **Q2** | I have chronic coughing and shortness of breath. The doctor suspects asthma. Could it be something else? | Chronic coughing and shortness of breath can be symptoms of various conditions, and while asthma is a common cause, other possibilities could include:   1. **Chronic Obstructive Pulmonary Disease (COPD):** This condition often affects people with a history of smoking, but it can also occur in non-smokers. It involves long-term breathing problems like chronic bronchitis or emphysema. 2. **Gastroesophageal Reflux Disease (GERD):** Acid reflux can irritate the airways and cause coughing or wheezing, which might be mistaken for asthma. 3. **Allergic Rhinitis or Sinusitis:** Allergies or sinus issues can lead to post-nasal drip, resulting in a chronic cough and breathing difficulties. 4. **Heart Failure:** Sometimes fluid buildup from heart problems can cause shortness of breath and coughing, especially when lying down. 5. **Infections:** Conditions like pneumonia or tuberculosis can cause chronic cough and breathing difficulties, though these are often accompanied by other symptoms like fever. 6. **Pulmonary Embolism:** A blood clot in the lung can lead to shortness of breath, cough, and chest pain, and it requires immediate attention.   It's important to follow up with your doctor for further testing, such as lung function tests, imaging, or blood tests, to confirm the diagnosis and rule out other conditions. | 4 | 3 | 4 | 4 | 4 | 4 | 4 |
| **Q3** | How can I distinguish asthma from other conditions, like chronic bronchitis or allergic cough? | Distinguishing asthma from other conditions like chronic bronchitis or allergic cough involves recognizing specific symptoms, triggers, and the pattern of respiratory issues. Here's how you can tell them apart:  **Asthma:**   1. **Wheezing**: A high-pitched whistling sound, especially during exhalation. 2. **Shortness of Breath**: Difficulty breathing, especially during physical exertion or at night. 3. **Coughing**: Often worsens at night or in the early morning. 4. **Chest Tightness**: Feels like something is squeezing or sitting on the chest. 5. **Triggers**: Common triggers include allergens (dust, pollen, pet dander), exercise, cold air, respiratory infections, or smoke. 6. **Variability**: Symptoms tend to fluctuate, with periods of worsening followed by improvement or periods of relative normalcy.   **Chronic Bronchitis:**   1. **Chronic Cough**: Persistent cough, often with mucus production lasting at least three months for two consecutive years. 2. **Sputum Production**: Coughing up mucus, which may be clear, yellow, or greenish. 3. **Wheezing and Shortness of Breath**: These symptoms are more constant and may worsen with physical exertion or respiratory infections. 4. **History**: Often associated with smoking or long-term exposure to irritants like dust or fumes. 5. **Less Variation**: Unlike asthma, the symptoms of chronic bronchitis are usually continuous, though they may worsen seasonally or during infections.   **Allergic Cough:**   1. **Dry Cough**: A persistent, dry cough, typically triggered by exposure to allergens (dust, pollen, mold, pet dander). 2. **Seasonal Pattern**: Symptoms often worsen during allergy season or when exposed to known allergens. 3. **No Wheezing or Chest Tightness**: Typically, there’s no significant wheezing or tightness in the chest, as seen in asthma. 4. **Nasal Symptoms**: Often associated with other allergic symptoms like sneezing, runny nose, or itchy eyes.   **Key Differences:**   - **Asthma** usually has a pattern of wheezing, chest tightness, and variability in symptoms. It’s often triggered by specific factors like allergens or exercise. - **Chronic bronchitis** presents with a persistent cough and mucus production, typically in the context of smoking or long-term exposure to irritants. - **Allergic cough** is often dry and triggered by allergens, without wheezing or significant chest tightness.   A healthcare provider can perform tests like **spirometry** to confirm asthma or chronic bronchitis, as well as evaluate your medical history, symptoms, and triggers. | 3 | 4 | 4 | 4 | 4 | 4 | 4 |
| **Q4** | What’s the difference between allergic asthma and bronchial asthma? | Allergic asthma and bronchial asthma are both types of asthma, but they differ in their causes and triggers:   1. **Allergic Asthma**:   **Cause**: This type of asthma is triggered by allergens (substances that cause allergic reactions), such as pollen, dust mites, mold, pet dander, or certain foods.  **Mechanism**: The immune system overreacts to allergens, leading to inflammation in the airways. It is often associated with other allergic conditions like hay fever or eczema.  **Symptoms**: Common symptoms include wheezing, coughing, shortness of breath, and chest tightness, especially when exposed to allergens.   1. **Bronchial Asthma**:   **Cause**: Bronchial asthma is a more general term that refers to asthma caused by inflammation and narrowing of the airways. It can be triggered by various factors, including respiratory infections, exercise, cold air, smoke, or environmental pollutants.  **Mechanism**: The inflammation in the bronchial tubes (airways) leads to constriction and difficulty breathing. It is not necessarily related to an allergic reaction.  **Symptoms**: Similar to allergic asthma, symptoms include wheezing, coughing, and shortness of breath, but these can be triggered by a wider range of factors.  In essence, **allergic asthma** is a subset of **bronchial asthma** where allergies specifically trigger the symptoms. | 4 | 3 | 4 | 4 | 4 | 4 | 4 |
| **Q5** | If I often feel short of breath, does that mean I have asthma, or could it be something else? | Shortness of breath can be caused by a variety of conditions, and asthma is one possibility, especially if it's accompanied by wheezing, coughing, or chest tightness. However, it could also be related to other conditions such as:  **Allergies**: Allergic reactions can cause shortness of breath.  **Respiratory infections**: Infections like pneumonia or bronchitis can lead to breathing difficulties.  **Heart conditions**: Conditions like heart failure or arrhythmias can cause shortness of breath.  **Anxiety or stress**: Panic attacks or anxiety can also make you feel short of breath.  **Chronic obstructive pulmonary disease (COPD)**: A progressive lung disease that affects airflow and can cause breathing problems.  Since you're already aware that you have asthma, it’s important to monitor your symptoms. If you're frequently experiencing shortness of breath, it may be related to your asthma, or it might indicate a need for a change in treatment or management. It would be a good idea to consult your doctor to evaluate whether it’s related to asthma or another condition. | 4 | 3 | 4 | 4 | 4 | 4 | 4 |
| **Q6** | I only have a cough without wheezing. The doctor says I have asthma. Is this diagnosis accurate? | Yes, it is possible to have asthma without wheezing. While wheezing is a common symptom of asthma, not all individuals with asthma experience it. Asthma symptoms can vary, and some people may only experience a persistent cough, especially at night or after exercise. This type of asthma is sometimes called "cough-variant asthma."  If your doctor has diagnosed you with asthma based on your symptoms and other factors, the diagnosis is likely accurate. However, it's important to follow up with your healthcare provider to discuss your symptoms, treatment plan, and any further tests that may help manage your condition. | 4 | 4 | 4 | 4 | 4 | 4 | 4 |
|  | **COVID-19 related** |  |  |  |  |  |  |  |  |
| **Q1** | As an asthma patient, am I at higher risk of contracting COVID-19? How should I protect myself? | Having asthma doesn't inherently increase the risk of contracting COVID-19, but it can make you more vulnerable to severe illness if you do get infected. People with asthma are more likely to experience complications due to the respiratory nature of the virus. To protect yourself, consider these steps:   1. **Follow preventive measures**:   **Wear a mask** when in crowded or indoor spaces.  **Wash hands frequently** and use hand sanitizers with at least 60% alcohol.  **Maintain physical distance** from others, especially in crowded areas.   1. **Monitor asthma**:   **Stick to your medication regimen** and ensure your asthma is under control to reduce your risk of complications.  **Carry your inhaler** and any other emergency medication you need.   1. **Stay informed**:   Follow guidance from healthcare providers and local health authorities.  Stay up to date on COVID-19 vaccinations, as they help prevent severe illness.   1. **Avoid triggers**:   Minimize exposure to environmental triggers like smoke, allergens, and pollution, which could weaken your immune response.  If you notice any worsening of asthma symptoms or suspect you might have COVID-19, it's important to seek medical attention promptly. | 4 | 4 | 4 | 4 | 4 | 4 | 4 |
| **Q2** | If I contract COVID-19, how should I manage it as someone with asthma? | If you have asthma and contract COVID-19, it's important to manage both your asthma and the virus carefully. Here are some key steps you should follow:  **1. Monitor Your Symptoms:**  **Track your asthma symptoms**: Pay attention to any changes in your usual asthma symptoms, like coughing, wheezing, shortness of breath, or chest tightness.  **Look for COVID-19 symptoms**: Common signs of COVID-19 include fever, cough, fatigue, and difficulty breathing. If you experience any severe symptoms (like persistent chest pain, confusion, or difficulty breathing), seek medical help immediately.  **2. Continue Your Asthma Medications:**  **Use inhalers as prescribed**: Continue using your daily asthma medications (including inhaled corticosteroids) and rescue inhalers if needed, even if you have COVID-19.  **Avoid stopping any medication**: Stopping your asthma medication can trigger an asthma attack and make it harder to breathe.  **3. Seek Medical Advice:**  **Consult your doctor**: Reach out to your healthcare provider to discuss your COVID-19 diagnosis, especially if you have moderate or severe asthma. Your doctor might adjust your treatment plan to better manage both conditions.  **4. Use a Peak Flow Meter:**  **Check your peak flow regularly**: This helps monitor how well your lungs are working. If your peak flow drops, it may indicate that your asthma is worsening.  **5. Stay Hydrated:**  **Drink plenty of fluids**: Staying hydrated can help loosen mucus and reduce the strain on your lungs.  **6. Rest and Isolate:**  **Get plenty of rest**: Your body needs energy to fight the virus.  **Self-isolate**: Follow guidelines to avoid spreading COVID-19 to others.  **7. Avoid Triggers:**  **Reduce exposure to asthma triggers**: Avoid smoke, strong odors, or allergens that might make your asthma worse while you're recovering from COVID-19.  **8. Emergency Care:**  **Know when to seek emergency help**: If you experience worsening shortness of breath, chest pain, or if you're unable to manage your asthma despite using your inhalers, go to the emergency room.  By staying on top of both your asthma treatment and COVID-19 symptoms, you'll have a better chance of managing both conditions effectively. | 4 | 4 | 4 | 4 | 4 | 4 | 4 |
| **Q3** | Should I get the COVID-19 vaccine? Will it affect my asthma? | Yes, it is generally recommended for individuals with asthma to get the COVID-19 vaccine. The vaccine does not affect asthma directly, and it can provide protection against severe illness, which is especially important for those with respiratory conditions like asthma.  The COVID-19 virus can potentially worsen asthma symptoms or lead to complications. Vaccination helps reduce the risk of severe infection, hospitalization, and other health issues. However, if you have concerns about specific allergies or reactions, it's always a good idea to consult with your healthcare provider to ensure the vaccine is safe for you based on your medical history and asthma condition.  Would you like more information on the vaccine options available for people with asthma? | 4 | 4 | 4 | 4 | 4 | 4 | 4 |
| **Q4** | Are COVID-19 symptoms more severe for asthma patients compared to others? What should I be especially mindful of? | Yes, COVID-19 symptoms can be more severe for asthma patients compared to others. Asthma affects the lungs and airways, making them more vulnerable to respiratory infections like COVID-19. This can lead to an increased risk of complications such as pneumonia, bronchitis, and difficulty breathing.  As an asthma patient, here are some key things to be especially mindful of:   1. **Monitor your symptoms closely**: If you start experiencing COVID-19 symptoms, like fever, cough, and shortness of breath, it’s essential to monitor your asthma symptoms as well. Asthma flare-ups can worsen with viral infections. 2. **Keep your inhaler or other medications on hand**: Make sure you have quick access to your rescue inhaler and any maintenance medications you need, as managing asthma properly is critical during respiratory infections. 3. **Stay up to date with vaccinations**: The COVID-19 vaccine has been shown to reduce the severity of infection, so ensuring you are vaccinated and have received any booster shots is important. 4. **Use a mask**: Since COVID-19 is a respiratory illness, wearing a mask when you're around others, especially in crowded places, can help reduce exposure and prevent potential viral infections. 5. **Stay indoors during high pollution or allergy season**: If you have asthma, it’s important to avoid triggers like pollution, dust, and allergens, which can worsen your condition. This is even more crucial during a viral infection. 6. **Seek medical advice promptly**: If you feel your asthma is becoming harder to manage or your breathing worsens, contact your healthcare provider. It's vital to receive the necessary treatment as soon as possible to avoid complications.   Would you like advice on any specific aspect of managing asthma during the pandemic? |  |  |  |  |  |  |  |
|  | **Mental health** |  |  |  |  |  |  |  |  |
| **Q1** | I have asthma and often feel very tired and weak. Is this normal? | Yes, it is quite common for people with asthma to feel fatigued or weak, especially if their asthma isn't well-controlled. Asthma can make it harder to get enough oxygen, leading to tiredness. Also, frequent asthma flare-ups, poor sleep due to breathing difficulties, or the side effects of asthma medications (like steroids) can contribute to fatigue.  It’s a good idea to talk to your doctor about these symptoms, as they can help optimize your asthma treatment plan. If you feel excessively tired or weak, they may want to check your asthma control, medication, or look for other possible causes. | 4 | 4 | 4 | 4 | 4 | 4 | 4 |
| **Q2** | Asthma is affecting my mood, and I often feel anxious. What can I do to manage this? | Managing asthma and the anxiety that comes with it can be challenging, but there are several strategies you can try to improve your mood and reduce stress:   1. **Breathe Properly**: Practice deep breathing exercises to help reduce anxiety and improve lung function. Techniques like diaphragmatic breathing or box breathing (inhaling for 4 counts, holding for 4, exhaling for 4) can calm your nervous system. 2. **Stay Consistent with Your Medication**: Ensure that you are taking your asthma medications as prescribed, including any preventive inhalers, to keep symptoms under control. This can reduce flare-ups and the stress that comes with them. 3. **Limit Triggers**: Identify and avoid asthma triggers (e.g., allergens, pollutants, or weather changes) to prevent worsening symptoms that could contribute to stress and anxiety. 4. **Mindfulness and Meditation**: These practices can help you stay present and reduce anxiety. Apps like Headspace or Calm offer guided meditations specifically for breathing and relaxation. 5. **Exercise**: Gentle exercises, like walking or yoga, can improve your lung capacity and reduce anxiety. Just make sure to check with your healthcare provider to ensure it’s safe for your asthma. 6. **Seek Professional Support**: If you find that anxiety is overwhelming, it might be helpful to speak with a therapist who can guide you through relaxation techniques or cognitive behavioral therapy (CBT) for anxiety. 7. **Sleep Well**: Ensure that you're getting enough rest. Poor sleep can worsen both asthma symptoms and anxiety, so maintaining a good sleep routine is crucial. 8. **Support Networks**: Talking to friends, family, or joining a support group for asthma or anxiety can help you feel understood and less isolated in your experience.   If anxiety becomes more intense or your asthma symptoms worsen, don’t hesitate to reach out to your healthcare provider for additional guidance and support. | 4 | 4 | 4 | 4 | 4 | 4 | 4 |
| **Q3** | Asthma makes me worry about sudden attacks. Is there a way to stay calm? | It's understandable to feel anxious about asthma attacks, but there are several strategies you can use to stay calm and manage anxiety:   1. **Practice deep breathing**: Slow, deep breaths can help reduce anxiety. Techniques like diaphragmatic or abdominal breathing encourage relaxation. 2. **Mindfulness and meditation**: Mindfulness exercises can help you focus on the present moment rather than worrying about potential triggers. Apps like Headspace or Calm can guide you through relaxation exercises. 3. **Have a plan**: Knowing exactly what to do in case of an asthma attack can reduce anxiety. Ensure you always have your medication with you and follow the steps outlined by your healthcare provider. 4. **Positive visualization**: Visualizing yourself managing an attack successfully can help build confidence. Imagine yourself using your inhaler and feeling relief. 5. **Regular exercise**: Engaging in gentle physical activity can improve your lung function and reduce anxiety over time. Make sure to consult your doctor for safe activities tailored to your condition. 6. **Stay informed**: Learn about asthma and its triggers so you can feel more in control of your health and less anxious about the unknown.   Would you like more tips on how to manage anxiety specifically related to asthma? | 4 | 4 | 4 | 4 | 4 | 4 | 4 |
|  | Lifestyle |  |  |  |  |  |  |  |  |
| **Q1** | How should I adjust my diet to avoid triggering asthma? | To help manage asthma through diet and avoid triggering symptoms, consider the following adjustments:   1. **Anti-inflammatory foods**: Incorporate foods rich in antioxidants and omega-3 fatty acids to help reduce inflammation. These include:   Fatty fish (salmon, mackerel, sardines)  Leafy greens (spinach, kale)  Berries (blueberries, strawberries)  Nuts (walnuts, almonds)  Olive oil   1. **Vitamin D**: Low vitamin D levels may be linked to increased asthma symptoms. Foods high in vitamin D include:   Fatty fish  Fortified foods (milk, cereal, and plant-based alternatives)  Eggs   1. **Avoid common food triggers**: Some foods may worsen asthma in certain individuals, including:   **Sulfites**: Found in wine, dried fruit, and some processed foods.  **Food additives and preservatives**: Like MSG (monosodium glutamate) and artificial colors.  **Dairy**: Some people find that dairy products can increase mucus production, which might trigger asthma symptoms.   1. **Limit processed foods**: Highly processed and sugary foods can lead to inflammation and worsen asthma symptoms. Focus on whole foods instead. 2. **Ginger and turmeric**: These have anti-inflammatory properties that may help manage asthma symptoms. 3. **Caffeine**: Some people find that caffeine (from coffee or tea) can help open up airways temporarily, though it should be consumed in moderation. 4. **Hydration**: Staying well-hydrated can help thin mucus and prevent it from blocking the airways. Drink plenty of water throughout the day.   Each person’s asthma triggers can differ, so it may be helpful to keep a food diary to track any specific foods that worsen symptoms for you. If you notice any patterns, avoid those foods and consult with your healthcare provider. | 4 | 4 | 4 | 4 | 4 | 4 | 4 |
| **Q2** | I have pets at home. Could this worsen my asthma symptoms? | Yes, pets can potentially worsen asthma symptoms, especially if you're allergic to pet dander. Pet hair, skin flakes, and saliva can trigger asthma attacks in sensitive individuals. It's important to manage exposure by keeping pets out of bedrooms, cleaning your home regularly, and using air purifiers. If you're unsure whether your asthma is affected by your pets, consider consulting with a doctor for specific advice and testing. | 4 | 4 | 4 | 4 | 4 | 4 | 4 |
| **Q3** | Can I exercise? What types of exercise are best for asthma patients? | Yes, you can exercise even with asthma! In fact, physical activity can help improve lung function and overall fitness. However, it's important to take certain precautions to avoid triggering asthma symptoms.  Here are some asthma-friendly exercises:   1. **Walking** – A low-impact exercise that is gentle on the lungs and joints. It's a great way to start building stamina. 2. **Swimming** – The warm, humid air around the pool can be easier on the lungs, and the water offers low-impact resistance, making it a fantastic option. 3. **Cycling** – If done at a moderate pace, cycling can be a great cardiovascular workout that’s less likely to trigger asthma symptoms. 4. **Yoga** – Focuses on controlled breathing and flexibility, which can help manage asthma symptoms over time. 5. **Strength training** – Lifting weights or bodyweight exercises can improve muscle strength without putting too much stress on your lungs.   **Tips for exercising with asthma:**  **Warm-up and cool down**: Start slow and gradually increase intensity.  **Use your inhaler** as prescribed before exercising if necessary.  **Avoid triggers**: Stay away from places with high pollen, pollution, or extreme temperatures.  **Monitor your symptoms**: If you start wheezing or feel short of breath, stop and use your inhaler if needed.  It's always a good idea to consult with your healthcare provider before starting a new exercise regimen. | 4 | 4 | 4 | 4 | 4 | 4 | 4 |
| **Q4** | I’m overweight. Could losing weight help improve my asthma symptoms? | Yes, losing weight can potentially help improve asthma symptoms. Being overweight can contribute to worsening asthma because excess weight may put pressure on the lungs and diaphragm, reducing lung capacity and making breathing more difficult. Additionally, excess body fat can lead to inflammation and other health issues that can trigger asthma.  Losing weight might improve overall lung function and reduce the severity of asthma attacks. It's always a good idea to work with your healthcare provider to develop a safe and effective weight-loss plan tailored to your needs, especially considering your asthma. Regular physical activity, when done safely, can also help manage asthma symptoms. | 4 | 4 | 4 | 4 | 4 | 4 | 4 |
| **Q5** | As an asthma patient, what should I pay attention to in daily life? | As an asthma patient, there are several key factors to keep in mind to manage your condition and avoid triggers:   1. **Medication Adherence**: Take your prescribed medications regularly, including inhalers or other treatments. Never skip doses, and consult your doctor before making any changes to your medication routine. 2. **Monitor Symptoms**: Keep track of any asthma symptoms such as shortness of breath, coughing, wheezing, or chest tightness. If symptoms worsen, consult your doctor to adjust your treatment plan. 3. **Avoid Triggers**: Identify and avoid your personal asthma triggers, such as:    - Allergens (dust, pollen, pet dander)    - Smoke or air pollution    - Strong odors or chemicals    - Cold air or weather changes 4. **Maintain a Clean Environment**: Keep your home free of dust, mold, and pet dander. Regularly clean areas that may collect allergens, such as carpets and upholstery. 5. **Exercise Wisely**: If exercise is a trigger, talk to your doctor about strategies, such as using your inhaler before exercising or participating in activities that don't stress your lungs too much. 6. **Emergency Plan**: Have an asthma action plan in place for emergencies. Know when and how to use your rescue inhaler and when to seek medical help. 7. **Stay Hydrated**: Drink plenty of water to help keep your airways hydrated and reduce the likelihood of mucus buildup. 8. **Weather Considerations**: Pay attention to weather changes, such as extreme cold or high humidity, as these can trigger asthma symptoms. Dress appropriately and consider staying indoors during poor air quality. 9. **Stress Management**: Emotional stress can sometimes trigger asthma symptoms, so practice relaxation techniques like deep breathing, yoga, or meditation.   By keeping these points in mind, you'll be better equipped to manage your asthma and reduce the chances of flare-ups. | 4 | 4 | 4 | 4 | 4 | 4 | 4 |
| **Q6** | My asthma symptoms often worsen during seasonal changes. Besides increasing my medication, what else can I do to alleviate the symptoms? | Managing asthma during seasonal changes can be tricky, but there are several strategies you can use alongside medication to help alleviate symptoms:   1. **Monitor Air Quality**: Stay updated on local air quality, pollen counts, and pollution levels. On days with poor air quality, try to limit outdoor activities. 2. **Keep Your Home Clean**: Dust, mold, and pet dander can trigger asthma. Regular cleaning, especially in areas prone to dust and mold, can help reduce these triggers. Using HEPA filters in your home's air purifiers or in your vacuum cleaner can also help. 3. **Control Allergens**: If seasonal allergies are a trigger, consider using an allergen-proof pillowcase and bedding. Showering and changing clothes after being outside can reduce exposure to pollen. 4. **Humidifiers or Dehumidifiers**: Use a humidifier in dry weather and a dehumidifier in humid conditions to keep the air at a comfortable level, as both extremes can irritate your airways. 5. **Stay Hydrated**: Drinking plenty of fluids helps keep the airways moist and can make it easier to breathe. 6. **Wear a Mask Outdoors**: If pollen or pollution is a major trigger during seasonal changes, wearing a mask can help reduce exposure while outside. 7. **Exercise Indoors**: During times of increased outdoor allergens, consider indoor activities to maintain your physical health without worsening asthma symptoms. 8. **Manage Stress**: Stress can exacerbate asthma symptoms, so engaging in relaxation techniques like deep breathing, yoga, or meditation may help reduce flare-ups.   If you find that your symptoms are still problematic, be sure to consult your healthcare provider for additional tailored advice. | 4 | 4 | 4 | 4 | 4 | 4 | 4 |
