# Supplementary material for: Assessing the Role of Large Language Models Between ChatGPT and DeepSeek in Asthma Education for Bilingual Individuals: Comparative Study
Source: JMIR Med Inform. 2025 Aug 13;13:e65365. doi: 10.2196/65365 (PMC12349887; doi:10.2196/65365)
Supplement: Multimedia Appendix 2 [file medinform-v13-e65365-s002.doc]

**Supplementary Table 2. ChatGPT-4.0’s Responses to Asthma-Related Inquiries in a Chinese-Language Context**

| **N.** | **Questions** | **Responses** | **Consistency** | **Completeness** | **Potential Bias** | **Reasoning Ability** | **Comprehension** | **Reliability** | **Safety** |
| --- | --- | --- | --- | --- | --- | --- | --- | --- | --- |
|  | **Basic Definitions** |  |  |  |  |  |  |  |  |
| **Q1** | 我今年25岁，最近咳嗽和胸闷，早上和夜间尤其明显，吃了阿莫西林也没效果，这会不会是哮喘？ | 你的症状（咳嗽、胸闷，尤其在早晨和夜间加重）确实与哮喘相似，但也可能是其他呼吸系统疾病，如过敏性咳嗽、慢性支气管炎或咳嗽变异性哮喘（CVA）。阿莫西林是抗生素，主要用于细菌感染，而如果你的症状是由哮喘或过敏引起的，抗生素通常不会有效。  建议你尽快就医，医生可能会安排肺功能检查（如支气管激发试验或支气管舒张试验）来确诊是否为哮喘。同时，你可以观察是否有以下情况：  运动、冷空气或接触过敏原（如尘螨、花粉）后症状加重  家族中是否有哮喘或过敏史  夜间或清晨咳嗽明显加重  如果症状持续或加重，建议及时就诊，以获得准确的诊断和治疗方案。 | 4 | 4 | 4 | 4 | 4 | 4 | 4 |
| **Q2** | 哮喘这种病常见吗？我是不是得了它？ | 1. 哮喘常见吗？  是的，哮喘是一种常见的慢性呼吸系统疾病，全球约有3亿多人受到影响。儿童和成年人都可能患上哮喘，尤其是在有过敏史、家族遗传或长期接触刺激性物质（如烟雾、花粉、尘螨）的人群中更常见。  2. 你是不是得了哮喘？  如果你有以下典型症状，可能需要警惕哮喘：  反复出现喘息（呼吸时有“哨音”）  气短或呼吸困难（尤其是在运动、冷空气或接触过敏原后加重）  咳嗽（特别是夜间或清晨）  胸闷（感觉肺部受压或无法完全呼吸）  但仅凭症状无法确诊哮喘，建议你去医院做专业检查，如 肺功能测试、支气管激发试验 或 过敏原检测，医生可以根据你的情况给出准确的诊断。  如果你近期有频繁咳嗽、气短或喘息的情况，建议尽快就医！  你可以告诉我你的具体症状，我可以帮你分析更详细的信息 | 4 | 4 | 4 | 4 | 4 | 4 | 4 |
| **Q3** | 我怀疑自己得了哮喘，怎么才能确诊？ | 如果你怀疑自己得了哮喘，建议尽快就医，医生会通过以下方法进行确诊：  1. 病史与症状评估  医生会询问你的症状，包括：  是否有喘息、气短、胸闷、慢性咳嗽等症状？  这些症状是否反复发作，特别是在夜间或清晨加重？  是否有过敏史、家族哮喘史，或接触某些触发因素（如花粉、灰尘、冷空气、运动）后症状加重？  2. 肺功能测试（最常见的确诊方法）  肺活量测定（spirmetry）：测量你的肺功能，特别是FEV₁（第一秒用力呼气量），如果吸入支气管扩张剂后FEV₁显著改善，提示哮喘。  峰流速测定（PEF）：每天早晚测量峰值呼气流速（PEF），如果波动较大，可能是哮喘。  3. 激发试验（必要时）  如果肺功能测试正常但仍怀疑哮喘，医生可能会让你做支气管激发试验（吸入低剂量组胺或乙酰胆碱等物质，看是否引发气道反应）。  4. 过敏测试  皮肤点刺试验或血清IgE检测，检查是否对花粉、尘螨等过敏，以判断是否存在过敏性哮喘。  5. 影像学检查（排除其他疾病）  胸部X光或CT，用于排除肺结核、慢性阻塞性肺病（CPD）等其他可能导致喘息的疾病。  建议行动  尽快就医，找呼吸科或过敏科医生进行检查。  记录症状（如发作时间、诱因、持续时间等），方便医生诊断。  如果已有症状严重影响生活，可临时使用支气管扩张剂（如沙丁胺醇）缓解，但不可长期自行用药。  如果确诊哮喘，医生会根据病情制定个体化的治疗方案，包括吸入性糖皮质激素（ICS）、支气管扩张剂等药物。 | 4 | 4 | 4 | 4 | 4 | 4 | 4 |
| **Q4** | 诊断哮喘需要做哪些检查？是通过血液还是其他检查？ | 诊断哮喘通常需要结合病史、体格检查和多个呼吸系统功能测试，而不仅仅依赖血液检查。以下是主要的检查方法：   1. 肺功能测试（主要检查方式）肺活量测定（Spirmetry）：测量肺的通气能力，检查是否存在气流受限。 2. 支气管舒张试验：患者吸入支气管扩张剂（如沙丁胺醇），然后再次进行肺活量测定。如果肺功能明显改善，则提示哮喘。   峰流速（PEF）测定：用于监测每日气道通畅度的变化。  2. 气道反应性测试（当肺功能检查不明确时使用）  支气管激发试验（如乙酰甲胆碱或运动激发）：观察气道是否对刺激物过度敏感。  3. 过敏检测（帮助查找哮喘诱因）  皮肤点刺试验：检测常见过敏原（如尘螨、花粉、霉菌）。  特异性IgE血液检测：检测是否存在针对特定过敏原的免疫反应。  4. 炎症相关检查 痰液嗜酸性粒细胞检查：检查气道炎症情况。 呼出气一氧化氮（FeN）检测：测量气道炎症水平，特别是嗜酸性炎症型哮喘。  5. 影像学检查（用于排除其他肺部疾病）  胸部X光或CT：排除肺结核、肺炎或慢阻肺（CPD）等疾病。  6. 血液检查（辅助检查） 外周血嗜酸性粒细胞计数：升高时提示可能存在嗜酸性粒细胞型哮喘。  总IgE检测：用于评估是否存在过敏相关哮喘。  总结  哮喘的主要诊断依据是肺功能测试（尤其是支气管舒张试验），血液检查可用于辅助判断病因（如过敏因素或嗜酸性炎症）。如有疑问，建议到呼吸科或变态反应科就诊，医生会根据个人情况选择合适的检查方法。 | 4 | 4 | 4 | 4 | 4 | 4 | 4 |
| **Q5** | 我最近咳嗽、喘气、胸闷，这些症状是不是代表我得了哮喘？ | 你的症状（咳嗽、喘气、胸闷）可能与哮喘有关，但也可能由其他疾病引起，例如：  过敏性支气管炎  呼吸道感染（如感冒、支气管炎、肺炎）  慢性阻塞性肺疾病（CPD）（如果你是长期吸烟者）  胃食管反流病（GERD）（胃酸反流可能引起慢性咳嗽和胸闷）  焦虑或心理压力（可能导致类似喘气的症状）  哮喘的典型特点：  反复发作：症状时好时坏，通常有诱因（如冷空气、灰尘、运动、花粉等）  夜间或清晨加重  缓解后正常：发作时喘不过气，使用支气管扩张剂（如沙丁胺醇）能缓解  家族史：有哮喘、过敏性鼻炎或湿疹的家族史  建议你做的事：  1️⃣ 观察症状是否有特定诱因（如天气变化、运动、接触粉尘等）  2️⃣ 去医院检查（肺功能检查、支气管舒张试验、过敏原测试等可帮助确诊）  3️⃣ 避免可能的触发因素（如烟雾、冷空气、花粉、动物毛发等）  如果症状持续加重，或者你呼吸急促到影响正常活动，建议尽快就医！ | 4 | 4 | 4 | 4 | 4 | 4 | 4 |
| **Q6** | 我得了哮喘，为什么医生说它可能与遗传有关？ | 哮喘的发生通常是多因素共同作用的结果，其中遗传因素在个体患病风险中起着重要作用。医生说你的哮喘可能与遗传有关，主要基于以下几个原因：  1. 家族遗传影响  如果你的父母或近亲中有人患有哮喘，你患病的风险会增加。例如：  父母一方有哮喘：子女患哮喘的风险约为 25%。  父母双方都有哮喘：子女患哮喘的风险可达 50% 甚至更高。  2. 相关基因的作用  研究发现，哮喘与多个基因相关，尤其是影响免疫系统和气道反应性的基因，例如：  IL-4、IL-5、IL-13：与过敏反应和炎症有关。  ADAM33：影响气道平滑肌的生长和收缩。  RMDL3/GSDMB：可能与气道过度敏感和炎症反应相关。  这些基因可能使你的免疫系统更容易对外界刺激产生过敏反应，从而引发或加重哮喘。  3. 过敏体质的遗传  哮喘与过敏密切相关，比如花粉、尘螨、霉菌、宠物毛发等过敏原。  如果你的父母有过敏性疾病（如过敏性鼻炎、湿疹、过敏性结膜炎），你更容易发展为过敏性哮喘。  4. 环境因素仍然重要  尽管遗传因素影响很大，但环境因素（如空气污染、吸烟、感染、饮食）也会触发或加重哮喘。如果你有家族遗传倾向，但避免环境诱因，哮喘的发作频率和严重程度可能会降低。  总结  医生说你的哮喘可能与遗传有关，是因为：  你的家族可能有人有哮喘或过敏性疾病。  相关基因可能使你的免疫系统更容易过度反应。  遗传和环境因素共同影响哮喘的发生。  如果你想更清楚地了解自己的遗传风险，可以考虑做过敏原测试或基因检测，并采取科学的管理措施来控制哮喘。 | 4 | 4 | 4 | 4 | 4 | 4 | 4 |
| **Q7** | 医生说我得了哮喘，为什么我的过敏原是阴性的？ | 哮喘的诊断主要依据 临床症状 和 肺功能检查，而不仅仅是过敏原测试的结果。你的过敏原测试是阴性的，可能有以下情况：  1. 非过敏性哮喘（内源性哮喘）  并非所有哮喘都是由过敏引起的，约 30%-50% 的哮喘患者对常见过敏原测试呈阴性。  可能的诱因：冷空气、运动、空气污染、病毒感染、情绪波动、药物（如阿司匹林）等。  2. 过敏原测试的局限性  过敏原测试（如皮肤点刺试验或血清IgE检测）只能检测 已知的常见过敏原，但并不代表你对所有过敏原都没有反应。  你可能对某些 未检测的过敏原 过敏，如霉菌、某些化学物质或食物。  3. 哮喘可能是由气道高反应性引起的  哮喘的本质是 气道慢性炎症和高反应性，即使没有明确的过敏原，气道仍然可能对 刺激物（如烟雾、香水、尘埃） 产生过度反应。  4. 过敏原测试可能存在误差  皮肤点刺试验受药物（如抗组胺药）、皮肤状态等影响，可能出现假阴性。  血清IgE水平可能在某些情况下不敏感，导致未检测出低浓度过敏原。  你可以怎么做？  遵循医生的治疗方案（如吸入激素、支气管扩张剂）。  记录发作诱因，观察是否有特定触发因素。  考虑支气管激发试验（如乙酰甲胆碱试验），以进一步明确哮喘的气道高反应性。  咨询医生是否需要进一步过敏原检测（如特异性IgE、斑贴试验）。  如果你的症状仍然难以控制，可以找呼吸科或变态反应科的医生做更详细的评估。 | 4 | 4 | 4 | 4 | 4 | 4 | 4 |
|  | **Clinical Features** |  |  |  |  |  |  |  |  |
| **Q1** | 哮喘的典型症状是什么？我应该怎么识别自己是不是得了哮喘？ | 哮喘的典型症状通常包括：  1.气喘：感觉呼吸急促或胸部紧迫，尤其在运动或暴露于过敏源时。  2.咳嗽：特别是在夜间或清晨时常发生，常常伴随有痰。  3.胸闷：感到胸部紧绷或难以深呼吸。  4.呼吸急促：可能会发现自己呼吸时不正常或有喘息的声音。  如果你怀疑自己可能得了哮喘，通常可以通过以下方式来识别：  如果在运动、接触过敏原（如花粉、尘螨、动物皮屑等）时出现上述症状。  如果症状在晚上或早晨加重。  如果症状持续数周或反复出现。  若出现这些症状，最好尽早就医，医生会通过问诊、体格检查和一些呼吸功能测试（如肺功能测试、峰流速测定等）来确诊是否患有哮喘。 | 4 | 3 | 4 | 4 | 4 | 4 | 4 |
| **Q2** | 除了咳嗽和喘息，哮喘还有其他不典型的症状吗？ | 是的，哮喘的症状除了咳嗽和喘息之外，还可能包括以下不典型的症状：  1.胸闷：一些哮喘患者可能会感到胸部紧绷或压迫感。  2.呼吸困难：哮喘发作时可能会出现呼吸急促，特别是在活动时或夜间。  3.咳痰：虽然不是所有哮喘患者都会有明显的痰液，但部分患者会在咳嗽时排出白色或透明的痰。  4.疲劳感：长期的哮喘症状可能会导致身体疲劳，因为身体需要更多能量来应对呼吸困难。  5.夜间咳嗽：一些患者在晚上或清晨出现咳嗽症状，这通常是哮喘的一种表现。  6.嗓音变化：有些人可能会在哮喘发作时出现声音嘶哑或咳嗽声较大。  这些症状有时可能较为隐匿，难以与其他疾病区分。如果你怀疑自己有哮喘，最好去看医生进行全面评估和诊断。 | 4 | 4 | 4 | 4 | 4 | 4 | 4 |
| **Q3** | 哮喘是否会引起其他身体上的问题，比如头痛或者食欲不振？ | 哮喘本身主要是影响呼吸系统的疾病，但它也可能引起一些与呼吸困难相关的身体问题。哮喘发作时，因呼吸不畅，可能导致氧气供应不足，进而引发头痛。头痛可能是由于缺氧、呼吸急促或睡眠质量差等因素引起的。  此外，哮喘药物的副作用也可能导致食欲不振。某些药物，如类固醇，可能会影响胃肠道，引起不适或改变食欲。长期的呼吸困难和疲劳感也可能导致食欲下降。  如果头痛和食欲不振与哮喘症状相关，建议咨询医生，确保正确管理哮喘并排除其他可能的原因。 | 4 | 4 | 4 | 4 | 4 | 4 | 4 |
| **Q4** | 如果我哮喘发作，应该会有什么样的表现？我应该怎么办？ | 哮喘发作时，通常会出现以下症状：  1.呼吸急促：你可能会感觉呼吸困难，尤其是在深呼吸时。  2.胸部紧迫感：有时胸部会感到压迫或紧绷。  3.咳嗽：特别是在夜间或早晨，咳嗽可能会加剧，尤其是干咳。  4.喘息：呼吸时可能会伴随“哨声”或类似于哮喘的声音。  5.气短：特别是在运动或接触到过敏源时，会感到无法正常呼吸。  如果发生哮喘发作时，你应该采取以下步骤：  1.使用吸入剂：如果你有快速缓解的药物（如短效β2激动剂吸入剂），立即使用它。通常情况下，药物能够迅速缓解症状。  2.保持镇定：尽量避免焦虑，保持冷静，集中注意力于缓慢、深呼吸。  3.找个安全的地方：坐下，避免剧烈运动或任何可能加重症状的活动。  4.避免过敏源：如果你知道自己的哮喘是由某些物质引起的，尽量远离这些过敏源。  5.寻求紧急帮助：如果症状没有缓解，或感到极度呼吸困难，及时寻求医疗帮助或拨打急救电话。  请注意，长期控制哮喘需要依赖医生的指导和药物管理。如果你还没有明确的治疗方案，建议咨询医生以制定合适的治疗计划。 | 4 | 4 | 4 | 4 | 4 | 4 | 4 |
| **Q5** | 为什么我白天没有问题，一到晚上就咳嗽喘憋？是哮喘的特征吗？ | 你在白天没有问题，但一到晚上就咳嗽、喘憋，确实可能是哮喘的一种特征。这种现象在医学上被称为夜间哮喘，它是哮喘患者常见的症状之一，通常发生在夜间或早晨早些时候。夜间哮喘的原因可能包括以下几种：  1.气道的自然变化：夜间身体处于休息状态，气道可能会出现轻微的收缩，导致呼吸困难。随着夜间体温和空气湿度的变化，气道可能更容易受刺激。  2.过敏源的暴露：在晚上，床上的过敏源（如尘螨、枕头、被褥上的霉菌等）可能会加剧过敏反应，导致气道收缩，进而引发咳嗽和喘息。  3.身体位置的变化：躺下时，重力的变化可能使得粘液更容易积聚在呼吸道中，导致堵塞，进而引发咳嗽或喘息。  4.气温和空气湿度：晚上的气温和空气湿度可能与白天不同，这会刺激敏感的气道，尤其是对哮喘患者来说。  5.激素水平的变化：体内的激素水平（如皮质醇）在夜间会自然下降，这可能会影响气道的炎症控制，使症状加重。  如果你在夜间经常出现咳嗽、喘息等症状，建议咨询医生，进行详细的检查和评估，确保诊断准确，并制定合适的治疗计划。医生可能会推荐使用特定的控制药物，帮助减少夜间症状。 | 4 | 4 | 4 | 4 | 4 | 3 | 4 |
| **Q6** | 我经常感到胸口发紧，这是不是哮喘发作的征兆？ | 胸口发紧可能是哮喘发作的症状之一，但也有其他多种原因可能导致这种感觉。例如，焦虑、胃酸倒流、心脏问题等也能引起胸部紧绷感。如果你有哮喘史，胸口发紧可能是哮喘发作的表现，通常伴随着咳嗽、喘息和呼吸急促。  建议你尽早就医，尤其是如果这种胸口发紧的感觉持续或加重，医生可以通过诊断确认病因并给出相应的治疗建议。 | 4 | 4 | 4 | 4 | 4 | 4 | 4 |
|  | **Treatment** |  |  |  |  |  |  |  |  |
| **Q1** | 我怀疑自己得了哮喘，在去医院之前可以自己使用什么药物缓解症状吗？ | 如果你怀疑自己得了哮喘，以下是一些可以暂时缓解症状的药物，但这并不能替代专业医生的诊断和治疗：  1.速效支气管扩张剂（如沙丁胺醇）：这类药物通常是吸入剂，可以迅速缓解哮喘引起的气道收缩，帮助缓解呼吸困难。沙丁胺醇（Ventlin）是常见的例子。如果你之前有过类似的处方，可以按照医生的建议使用。  2.吸入型类固醇（如果医生已经开过）：对于长期控制症状的药物，如果你曾经被开过吸入型类固醇，如布地奈德（Pulmicrt）等，可以按照医嘱使用。  3.抗组胺药：如果你的哮喘症状与过敏有关，可以适量服用抗组胺药（例如扑尔敏），但这通常只能缓解一些过敏症状，对哮喘的急性发作帮助有限。  注意事项：  在使用任何药物之前，最好还是咨询医生，尤其是如果你从未被诊断为哮喘，或者之前没有过相关药物的使用经验。  如果你感觉呼吸急促，胸闷，或者症状加重，应尽快去医院就诊，尤其是在使用药物后没有显著缓解时。  及时寻求医生的帮助是最重要的，哮喘症状如果不能得到适当的管理，可能会加重并导致严重的呼吸困难。 | 4 | 4 | 4 | 4 | 4 | 4 | 4 |
| **Q2** | 我被确诊为哮喘，我想知道我应该用什么药? | 哮喘的治疗药物主要分为两类：长期控制药物和急性发作药物。具体使用哪种药物，应根据医生的建议，结合个人的病情。  1.长期控制药物：用于控制哮喘症状，减少发作的频率和严重程度。  吸入型皮质类固醇（例如：布地奈德、氟替卡松）：是最常用的长期控制药物，能减少气道炎症。  长效β2激动剂（例如：沙美特罗、福莫特罗）：通常与皮质类固醇联合使用，用于维持气道扩张。  白三烯受体拮抗剂（例如：孟鲁司特）：适用于轻度哮喘患者或与其他药物联合使用。  免疫抑制剂（例如：美托洛尔、环孢素）：用于更严重的病例。  2.急性发作药物：用于缓解哮喘急性症状。  短效β2激动剂（例如：沙丁胺醇、特布他林）：是一种快速舒张气道的药物，用于缓解急性哮喘发作。  抗胆碱能药物（例如：异丙托溴铵）：可与β2激动剂合用，以加强急性缓解效果。  请根据医生的指导使用药物，并定期随访调整治疗方案。 | 4 | 4 | 4 | 4 | 4 | 4 | 4 |
| **Q3** | 医生让我吸入布地奈德，我能知道这个药会有什么副作用吗？ | 布地奈德（Budesnide）是一种常用于治疗哮喘和慢性阻塞性肺病（CPD）的吸入类药物，它是一种类固醇药物，主要通过减少气道的炎症来起效。像所有药物一样，布地奈德可能会引起一些副作用，尽管并不是每个人都会经历这些副作用。常见的副作用包括：  1.口腔和咽喉不适：吸入布地奈德可能导致口腔或喉咙干涩、刺激或感染。为了减少这些症状，使用吸入器后，建议漱口并吐出水，以清除药物残留。  2.喉部感染：长期使用类固醇药物可能会增加口腔和喉咙感染的风险，尤其是念珠菌感染（俗称鹅口疮）。  3.声音沙哑或咳嗽：这也是吸入类固醇药物的常见副作用。  4.头痛：有些人可能会感到头痛或头晕。  5.恶心或消化不良：虽然较少见，但布地奈德也可能引起轻微的消化不适。  6.长期使用的副作用：如果布地奈德长期使用，可能会影响身体的免疫功能或骨骼健康，导致骨质疏松、眼压升高等问题。  如果你有任何不适或副作用加重的情况，应该立即向医生报告，以便调整用药方案或采取其他措施。 | 4 | 4 | 4 | 4 | 4 | 4 | 4 |
| **Q4** | 我使用了布地奈德福莫特罗后，心跳很快，觉得不舒服，这是不是正常的反应？ | 布地奈德福莫特罗（Budesnide/frmterl）是一种常用于治疗哮喘和慢性阻塞性肺病（CPD）的药物，结合了糖皮质激素和长效β2受体激动剂。心跳加快或心悸是使用这种药物后可能会出现的副作用之一，尤其是福莫特罗这种成分具有刺激心脏的作用，可能导致心跳加快或不适。  如果你感到不舒服，或者症状持续或加重，建议你尽快联系医生，评估是否需要调整药物剂量或更换治疗方案。对于这种情况，医生可能会调整药物的使用方式或考虑其他替代治疗方法。 | 4 | 4 | 4 | 4 | 4 | 4 | 4 |
| **Q5** | 我爸爸也有哮喘，使用沙美特罗替卡松后嗓子痛、声音嘶哑，这是不是药物的副作用？ | 沙美特罗替卡松是一种常用于治疗哮喘的组合药物，通常由沙美特罗（长效β2激动剂）和替卡松（类固醇）组成。它们能够帮助控制哮喘症状并减少炎症。然而，有些人可能会出现副作用，包括嗓子痛和声音嘶哑。  这些症状可能是由于药物中的类固醇成分引起的，类固醇可以导致喉部和声带的刺激。为了减少这些副作用，使用者可以采取以下预防措施：  在使用吸入药物后，用水漱口或刷牙，以减少药物残留在口腔和喉部的时间。  检查是否正确使用吸入器，避免药物进入喉咙和口腔。  如果症状持续或严重，建议就医，医生可能会调整用药剂量或选择其他治疗方法。  如果你爸爸的症状比较严重或持续，建议他联系医生进行评估。 | 4 | 4 | 4 | 4 | 4 | 4 | 4 |
| **Q6** | 有没有新的治疗方法，能让我的哮喘不再频繁发作？ | 哮喘的治疗方法在不断发展，近年来一些新的疗法已经被提出，并显示出良好的效果，帮助减少哮喘的发作频率。以下是几种可能对您有所帮助的治疗方法：  1.生物制剂（Bilgics）： 生物制剂是一类通过靶向免疫系统中特定蛋白质来减轻炎症的药物。对于那些药物控制效果较差的哮喘患者，生物制剂可以有效地减少哮喘发作的频率。这些药物包括：  Dupilumab (Dupixent)：用于治疗由过敏引发的哮喘。  malizumab (Xlair)：针对由过敏引起的哮喘，特别是对某些过敏原的反应较强的人群。  Meplizumab (Nucala) 和 Reslizumab (Cinqair)：针对嗜酸性粒细胞过多的哮喘患者有效。  2.免疫疗法（Allergen Immuntherapy）： 如果您的哮喘是由特定过敏原（如花粉、尘螨等）引发的，免疫疗法可能是一个长效的治疗选择。这种治疗方式通过逐渐增加您对过敏原的耐受性来减少过敏反应，从而减少哮喘症状。  3.持续的药物调整和个性化治疗： 近年来，哮喘治疗的个性化程度逐步提高。医生可能会根据您的具体病情和哮喘类型，调整治疗方案，使用更精细的药物来避免过多的药物负担，并提高效果。某些患者可能需要更频繁的使用短效吸入性支气管扩张剂，或者采用长效药物来控制症状。  4.吸入类皮质类固醇（ICS）和长期控制药物： 在一些新的治疗方案中，ICS药物的剂量和使用方式得到了优化，更加精细化的药物管理可以帮助减少哮喘发作的频率。  建议您咨询您的医生，根据您的哮喘类型和发作频率，制定一个适合您的治疗计划。同时，保持与医生的定期沟通，适时调整治疗方案以达到最佳效果。 | 4 | 3 | 4 | 4 | 4 | 4 | 4 |
| **Q7** | 治疗哮喘吸入药物和口服药物有什么区别？哪个更有效？ | 治疗哮喘的吸入药物和口服药物在药理作用、使用方式和效果方面有所不同：  吸入药物  1.作用机制：吸入药物通过气道直接作用，能够迅速发挥药效。它们主要是局部药物，直接作用于呼吸道，减少全身副作用。  2.常见类型：  支气管扩张剂：如沙丁胺醇等，用于缓解哮喘急性症状。  抗炎药物：如吸入性糖皮质激素（ICS），用于控制哮喘的慢性炎症。  3.效果：吸入药物通常更快速有效，因为药物直接作用于目标部位，即气道和肺部。吸入治疗常用于长期控制哮喘和急性发作。  4.副作用：因为药物局部作用，副作用通常较少，但长时间使用可能会出现喉咙刺激或口腔念珠菌感染。  口服药物  1.作用机制：口服药物通过消化系统进入血液循环，再作用于全身。它们可能对气道产生间接的影响，但由于需要通过全身循环，药效的发作通常较慢。  2.常见类型：  口服糖皮质激素：如泼尼松，用于控制急性哮喘发作或严重的哮喘症状。  口服抗白三烯药物：如孟鲁司特，帮助减少过敏反应。  3.效果：口服药物通常用于控制更为严重的哮喘症状或在吸入药物无法控制症状时使用。它们通常比吸入药物慢，但效果较持久。  4.副作用：口服药物可能引起较多的全身性副作用，如体重增加、水肿、高血糖和骨质疏松等，尤其是长期使用时。  哪个更有效？  吸入药物通常是哮喘管理的首选，因为它们能够更直接地作用于气道，效果更快且副作用较小。  口服药物通常用于更严重的情况，或者当吸入药物效果不足时。长期口服药物可能带来更大的副作用风险，因此应在医生指导下使用。  总结来说，吸入药物在哮喘控制中通常更有效，但口服药物对于急性发作或重症哮喘仍然是重要的治疗手段。 | 4 | 4 | 4 | 4 | 4 | 4 | 4 |
| **Q8** | 我正在使用治疗哮喘的药物，但我今天忘记用药了，应该怎么补救？ | 如果你今天忘记使用哮喘药物，补救的步骤取决于你正在使用的药物类型：  1.吸入型药物（如吸入型皮质类固醇）：  如果你忘记使用了一剂药物，并且记得是在一天中的常规时间段之后忘记使用，通常可以尽快补充这剂药物。  如果距离下次用药还有很短时间（例如几个小时），可以跳过这剂药物，按照原计划继续使用下次的剂量。  注意：不要使用两倍剂量来弥补错过的剂量。  2.短效或长效支气管扩张剂（如沙丁胺醇或沙美特罗）：  如果你错过了短效支气管扩张剂的使用，并且症状有所加重，可以立即使用补充剂量。  长效药物（如长效支气管扩张剂）通常需要按时补充，但如果错过时间，可以等到下一个时间点再使用。  3.复合药物（如联合型吸入剂）：  同样，根据药物类型，尽量补充错过的剂量，但不应加倍剂量。  总之，最安全的做法是根据药物说明书或医生的指导来处理，如果有不确定的地方，最好咨询医生或药剂师。记得，长期遵循治疗计划非常重要，避免错过剂量。 | 4 | 4 | 4 | 4 | 4 | 4 | 4 |
| **Q9** | 如果我不想一直依赖药物，能通过饮食或运动来控制哮喘吗？ | 哮喘的管理通常包括药物治疗，但饮食和运动也能在一定程度上帮助控制病情，作为药物治疗的辅助。以下是一些饮食和运动方面的建议：  饮食  1.避免触发食物：一些食物可能会加剧哮喘症状，如含有高盐、人工色素或防腐剂的食物。避免这些可能的触发物可以减少哮喘的发作。  2.增加抗炎食物：富含抗氧化剂和欧米伽-3脂肪酸的食物（如蓝莓、深海鱼、坚果、橄榄油等）有助于减轻体内的炎症反应，可能有助于哮喘的控制。  3.高纤维饮食：富含纤维的食物（如全谷类、蔬菜和水果）有助于改善肺部健康，可能帮助缓解哮喘症状。  运动  1.有氧运动：适度的有氧运动（如慢跑、游泳、骑车）可以增强心肺功能，提高耐力，帮助改善哮喘控制。选择低过敏风险的环境进行运动，比如在空气清新、无污染的地方。  2.避免剧烈运动：剧烈运动可能会诱发哮喘症状，尤其是在寒冷的天气里。建议进行温和的热身，避免在气温极低或污染严重的情况下运动。  3.呼吸练习：某些呼吸练习（如腹式呼吸、普拉提中的呼吸控制）可以帮助改善肺部功能和增强对气道的控制。  虽然饮食和运动可以有助于控制哮喘，但如果你的哮喘症状严重，药物治疗仍然是必要的。建议与你的医生沟通，探讨如何在保证健康的情况下减少对药物的依赖。 | 4 | 4 | 4 | 4 | 4 | 4 | 4 |
| **Q10** | 诊断哮喘后医生让我吸入激素，我以后会不会有激素依赖？ | 吸入激素（通常是类固醇）是治疗哮喘的常用药物之一。它能有效减轻气道的炎症，预防哮喘症状和急性发作。通常，如果正确使用吸入激素，它并不会导致激素依赖。  不过，如果长期过量使用吸入激素，可能会有一些副作用，如口腔感染、声音嘶哑等，但这些副作用通常是可控的，并不等同于“激素依赖”。激素依赖通常是指身体对激素产生了适应性，需要更多的激素来发挥效果，这在吸入激素治疗中较少发生。  重要的是，按医生的指导正确使用药物，定期复诊调整药物剂量。这样可以确保治疗效果，同时减少潜在副作用的风险。如果有任何不适或疑虑，建议及时向医生咨询。 | 4 | 4 | 4 | 4 | 4 | 4 | 4 |
|  | **Assessment and Management** |  |  |  |  |  |  |  |  |
| **Q1** | 哮喘是否会影响我的寿命？我应该如何管理病情以避免严重后果？ | 哮喘本身通常不会直接影响寿命，但如果没有得到适当管理，它可能会导致严重的并发症，如急性哮喘发作或慢性肺功能下降，从而影响生活质量。在一些情况下，未控制的哮喘可能增加心肺问题的风险。  要有效管理哮喘并避免严重后果，您可以遵循以下几点建议：  1.遵循治疗计划：按照医生的建议，定期使用控制药物（如吸入性类固醇）来减少气道炎症，并在需要时使用急救药物（如快速起效的支气管扩张剂）。  2.避免触发因素：确定并避免可能引发哮喘发作的因素，例如空气污染、过敏原、烟草烟雾、运动或感染。  3.定期监测：使用峰流速仪监测呼吸功能，帮助您及早发现病情变化。记录症状和药物使用情况，与医生定期沟通。  4.保持健康生活方式：保持适当的体重，避免吸烟，避免过度劳累，保持良好的睡眠，增强免疫力。  5.接种疫苗：如流感疫苗和肺炎疫苗等，有助于预防可能加重哮喘症状的呼吸道感染。  通过积极管理哮喘，您可以大大减少发作的频率和严重性，从而保持较好的生活质量并降低并发症的风险。如果您有任何症状变化或对治疗有疑问，及时与医生沟通非常重要。 | 4 | 4 | 3 | 4 | 4 | 4 | 4 |
| **Q2** | 我有哮喘，但还是抽烟了，这会加重病情吗？ | 是的，吸烟会加重哮喘病情。吸烟是哮喘的主要触发因素之一，它会刺激气道，增加炎症，使得气道更加狭窄，从而导致哮喘症状加重，比如呼吸急促、咳嗽、喘息等。长期吸烟还可能降低药物治疗的效果，并导致肺功能进一步恶化。  为了控制哮喘，最好的做法是戒烟。如果你感到戒烟困难，寻求医生的帮助和支持可能对你有所帮助。 | 4 | 4 | 4 | 4 | 4 | 4 | 4 |
| **Q3** | 哮喘发作时有哪些症状，我应该什么时候去医院治疗？ | 哮喘发作时，常见的症状包括：  1.气短：呼吸困难，特别是在呼气时感觉空气流动受限。  2.喘息：呼吸时会发出哮哮的声音，尤其是在呼气时。  3.咳嗽：通常是干咳，特别是在夜间或清晨更为严重。  4.胸部紧迫感：感觉胸部被压迫或紧缩。  5.呼吸急促：喘不过气来，甚至在轻微活动后也感到极度疲劳。  何时去医院治疗：  如果你发现自己无法通过常规的吸入性药物（如沙丁胺醇）缓解症状。  如果呼吸困难持续加重，无法说话或进行日常活动。  如果胸部紧迫感严重，呼吸急促不止。  如果发作后症状未能在短时间内得到控制，且症状加重。  如果你或他人出现这些症状并且没有改善，建议尽早就医，尤其是在家中的药物无法缓解症状时。紧急处理可以防止病情进一步恶化。 | 4 | 4 | 4 | 4 | 4 | 4 | 4 |
| **Q4** | 如果我得了哮喘，怎么评估自己病情的严重程度？有自测方法吗？ | 评估哮喘病情严重程度可以通过以下几种方法来进行自测：  1. 使用峰值流速计：  峰值流速计（PEF）是一个简单的小型设备，可以帮助你测量你呼吸时的最大流速。通过定期测量和记录你的峰值呼气流速，你可以了解自己的呼吸功能。  如何使用：  每天早上和晚上测量一次，最好在没有使用吸入药物时。  根据医生的建议，将结果与你的“个人最佳值”进行对比。低于此值可能意味着你的哮喘控制不佳。  常见的评估标准：  绿区（80%-100% 的最佳值）：哮喘控制良好。  黄区（50%-80% 的最佳值）：需要调整治疗。  红区（低于50% 的最佳值）：需要立即就医或使用救援药物。  2. 观察症状：  日常症状如咳嗽、喘息、胸闷和呼吸急促也可以帮助评估病情的严重性。如果症状在白天或夜间频繁出现，或者需要使用短效支气管扩张剂（救援药物）频繁缓解症状，可能表明病情加重。  3. 药物使用频率：  长期控制药物（如吸入类固醇）应每天按时使用，控制哮喘症状。  如果你需要更多的救援药物（如沙丁胺醇）来缓解症状，尤其是夜间使用，可能意味着哮喘控制不佳。  4. 评估活动限制：  哮喘是否限制了你的日常活动，如工作、运动或休息时的活动，若有明显限制，可能表示病情加重。  5. 症状频率与严重性：  每周超过两次的症状发作，或者夜间咳嗽每个月超过一次，说明可能需要调整治疗。  6. 医生评估：  最重要的是定期去看医生，尤其是在病情加重时。医生可以通过检查肺功能、进行过敏测试等手段，全面评估病情。  请注意，自测只能作为日常管理的参考，任何病情变化或加重，尤其是出现严重喘息或呼吸困难时，应该尽快寻求专业医疗帮助。 | 4 | 4 | 4 | 4 | 4 | 4 | 4 |
| **Q5** | 我是一个哮喘患者，每天我都在用药，病情稳定后，我可以停药吗？ | 对于哮喘患者，是否可以停药取决于个体的病情和医生的建议。如果病情稳定，医生通常会建议逐步减少药物剂量，但不能自行停药。哮喘管理需要长期控制，尤其是对于那些已经达到了稳定状态的患者，停药时需要谨慎，并在医生的指导下进行。  如果你觉得病情得到很好的控制，建议与医生讨论停药的可能性。医生会根据你的具体情况制定合理的治疗计划。 | 4 | 4 | 4 | 4 | 4 | 4 | 4 |
| **Q6** | 我怎样使用峰流速计来监测我的哮喘症状？什么时候使用它最有效？ | 峰流速计（Peak Flw Meter）是一种便捷的设备，用于监测你的呼吸流速，帮助你了解哮喘的控制情况。通过测量最大呼气流速（即峰流速），你可以观察到哮喘症状是否加剧或有所改善。以下是如何使用峰流速计和最佳使用时机：  使用峰流速计的步骤：  1.确保设备清洁：每次使用之前，确保峰流速计干净并准备好。  2.站立或坐直：保持身体直立，避免弯腰。  3.深吸气：深深吸气，将肺部充满空气。  4.用力呼气：用力将空气迅速吹出，尽可能快速而强力。  5.记录读数：峰流速计会显示一个数字，记下这个值。可以进行三次测量，记录最高值。  6.对比你的个人最佳值：通过与自己日常的峰流速比较，可以了解当前的呼吸情况。  使用峰流速计的最佳时机：  1.每天早晨和晚上：每天早晚测量峰流速，了解你的基础值和变化。  2.症状加剧时：当你感觉哮喘症状加重（如呼吸急促、胸闷或咳嗽）时，使用峰流速计检查你的流速。  3.在运动前后：如果你参与体育活动或锻炼，运动前后检查峰流速，确保活动没有影响到你的呼吸。  4.在环境变化时：如果你处于一个有污染或过敏源的环境中，使用峰流速计监测你的呼吸情况。  结果解读：  绿色区域：如果你的峰流速接近个人最佳值，表示哮喘得到良好控制。  黄色区域：如果流速低于个人最佳值的80%，你可能处于症状加重的风险中，需进行药物调整。  红色区域：如果流速低于个人最佳值的50%，说明你的哮喘可能处于严重发作状态，应该立即寻求医疗帮助。  定期监测峰流速有助于你早期发现哮喘症状的变化，及时采取措施控制病情。 | 4 | 4 | 4 | 4 | 4 | 4 | 4 |
| **Q7** | 哪些因素会影响哮喘的预后，我该如何避免？ | 哮喘的预后可能受到多个因素的影响，以下是一些常见的因素以及你可以采取的措施来避免或减轻其影响：  1. 哮喘的控制情况  影响因素：如果哮喘症状没有得到有效控制，可能会导致急性发作或长期的肺功能损害。  如何避免：坚持按时服用药物（如吸入性类固醇、支气管扩张剂等），定期复查，遵循医生的治疗计划，并避免接触过敏源。  2. 过敏原暴露  影响因素：过敏原（如花粉、尘螨、宠物毛发、霉菌等）可能诱发或加重哮喘。  如何避免：尽量减少接触过敏原，保持家中清洁，定期更换床单，使用防尘螨的床垫和枕头套，外出时佩戴口罩。  3. 环境污染  影响因素：空气污染（如二氧化硫、氮氧化物、臭氧等）和烟草烟雾等有害物质可能刺激气道，加重哮喘症状。  如何避免：尽量避免在空气污染严重时外出，避免二手烟，保持室内空气清新，使用空气净化器。  4. 肥胖  影响因素：肥胖会加重哮喘症状，可能影响肺功能，导致哮喘控制困难。  如何避免：保持健康的体重，均衡饮食，定期锻炼，避免暴饮暴食。  5. 药物依从性  影响因素：不按时或不规范地使用药物可能导致症状加重，影响哮喘的长期控制。  如何避免：坚持医生的治疗方案，确保按时使用药物，定期复诊，跟踪症状。  6. 其他慢性疾病  影响因素：如鼻窦炎、胃食管反流病等其他慢性疾病，可能与哮喘的症状相互作用，导致哮喘控制难度增大。  如何避免：合理治疗其他慢性疾病，避免引发哮喘的因素。  通过积极管理和避免这些风险因素，你可以有效提高哮喘的预后，减少症状发作的频率和严重性。如果你有任何疑虑或症状变化，及时咨询医生是非常重要的。 | 4 | 4 | 4 | 4 | 4 | 4 | 4 |
| **Q8** | 我患有哮喘2年了，我现在已经没有喘息的症状了，但活动后还有些气短，应该怎么做？ | 既然你已经没有喘息的症状，但在活动后依然有气短的情况，可能是因为运动后气道仍然较为敏感或是轻微的气道收缩。以下是一些可能帮助缓解气短症状的方法：  1.热身和放松：在进行任何运动之前，确保进行充分的热身，并在结束时做适当的放松，帮助气道适应运动强度，减少剧烈的运动后气短。  2.调整运动强度：避免剧烈的运动，选择较为温和且能逐渐增加强度的活动，比如散步、瑜伽或游泳，避免突然增加运动量。  3.使用预防性药物：如果你的医生建议使用吸入型药物进行预防性治疗，确保按时使用它们，尤其是在运动前。  4.控制过敏源：有时过敏也是引起气短的原因，保持空气清新，避免过敏源，尤其是在运动前和运动过程中。  5.增强肺部健康：考虑进行肺功能训练或呼吸练习，增强气道耐受力。比如，深呼吸训练有助于改善呼吸效率。  6.监测症状：记录气短的出现频率和强度，以便与你的医生讨论，看看是否需要调整治疗方案。  如果症状持续或加重，建议咨询你的医生，评估是否需要调整哮喘治疗方案。 | 4 | 4 | 4 | 4 | 4 | 4 | 4 |
| **Q9** | 我有哮喘，能彻底治愈吗？以后还能正常生活吗？ | 哮喘目前没有完全治愈的方法，但可以通过适当的治疗和管理来控制症状。大多数人通过使用药物（如吸入性类固醇、支气管扩张剂等）和避免触发因素，能够过上相对正常的生活。治疗的关键是根据个人的病情调整药物，定期复查，并遵循医生的建议。  有些人可能随着年龄的增长或通过长期控制，症状会有所缓解，但这并不意味着完全治愈。重要的是保持健康的生活方式，避免可能的诱因，并根据医生的建议进行持续管理。 | 4 | 4 | 4 | 4 | 4 | 4 | 4 |
| **Q10** | 我是一个哮喘患者，我的病情控制得不错，为什么感觉身体状况并没有好转？ | 即使哮喘得到了较好的控制，你仍然可能会感觉身体状况没有显著好转，原因可能有几个：  1.长期炎症的影响：哮喘是由气道长期的慢性炎症引起的，即使症状得到控制，炎症可能依然存在，这可能会影响到身体的整体感受。  2.体力活动受限：如果哮喘在过去影响了你的体力或运动能力，即使控制得好，恢复到正常水平可能需要时间。  3.其他健康问题：除了哮喘，可能还有其他健康问题（如过敏、呼吸系统疾病等）影响你的身体状况，导致你没有感到明显的好转。  4.心理因素：长期管理慢性疾病可能导致心理上的疲惫感，即使生理上状况有所改善，心理上的压力或焦虑感也可能影响到你对健康的感知。  如果你有任何疑虑，最好和医生沟通，看看是否有其他因素影响你的身体状况，或者是否需要调整治疗方案。 | 4 | 4 | 4 | 4 | 4 | 4 | 4 |
| **Q11** | 医生说我是哮喘，如果我的症状在使用药物后没有缓解，我该如何判断是否需要再就医？ | 如果在使用药物后你的哮喘症状没有得到缓解，可能需要考虑重新就医。你可以根据以下几种情况判断是否需要就医：  1.症状持续加重：如果你的呼吸困难、咳嗽、喘息或胸闷持续或加重，可能表示药物没有起效，或你的哮喘控制不理想。  2.急性发作：如果出现急性哮喘发作，药物无法快速缓解症状，或者你发现自己需要比平时更多的急救药物（如吸入型支气管扩张剂），应该立即就医。  3.夜间症状：如果你发现哮喘症状在夜间加重，或者影响到你的睡眠，这也可能是需要重新评估治疗的信号。  4.体力活动受限：如果你发现运动或日常活动受到哮喘症状的限制，或症状让你难以正常生活，可能需要调整治疗方案。  5.其他合并症或反应：如果你使用药物后出现任何异常反应，如药物过敏、呼吸困难加重等，也应尽快就医。  建议及时联系你的医生或前往急诊，特别是如果你感到症状严重且无法控制时。 | 4 | 3 | 4 | 4 | 4 | 4 | 4 |
|  | **Differential Diagnosis** |  |  |  |  |  |  |  |  |
| **Q1** | 除了哮喘，还有哪些其他病症会让我出现咳嗽和喘息？ | 除了哮喘，以下是一些可能引起咳嗽和喘息的常见病症：  1.慢性阻塞性肺病（CPD）：这是一种渐进性的肺部疾病，通常由长期吸烟引起。症状包括持续性咳嗽、咳痰和喘息。  2.过敏性反应：如过敏性鼻炎或过敏性支气管炎，接触到花粉、灰尘或动物皮屑等过敏原时，可能会导致咳嗽、喘息和呼吸急促。  3.支气管炎：急性或慢性支气管炎（气管和支气管的炎症）可导致咳嗽、痰多和喘息，通常与感染有关。  4.肺炎：肺部感染引发的炎症，可能导致咳嗽、喘息、呼吸困难和发热。  5.胃食管反流病（GERD）：胃酸反流到食管并进入呼吸道，可能引发咳嗽、喉咙不适和喘息。  6.心力衰竭：心脏无法有效泵血，可能导致肺部积液，从而引起呼吸困难、咳嗽和喘息。  7.肺部肿瘤或肺癌：某些肺部肿瘤或癌症可能导致持续的咳嗽和喘息，尤其是在病情进展时。  8.过敏性哮喘或非过敏性哮喘：哮喘症状可能由于过敏或环境因素引起，不一定与免疫系统的过敏反应直接相关。  9.呼吸道感染：如流感、感冒等病毒性感染会引起暂时的咳嗽和喘息。  如果你有持续的咳嗽和喘息，建议尽早去看医生，以便确定具体原因并接受适当的治疗。 | 4 | 4 | 4 | 4 | 4 | 4 | 4 |
| **Q2** | 我有慢性咳嗽和气短，医生怀疑是哮喘，是否可能是其他疾病？ | 慢性咳嗽和气短确实是哮喘的常见症状，但也可能与其他一些疾病有关。除了哮喘之外，以下几种疾病也可能引起类似症状：  1.慢性阻塞性肺病（CPD）：这是一种慢性肺部疾病，通常与吸烟或长期接触空气污染物有关，症状包括咳嗽、气短和呼吸困难。  2.过敏性鼻炎：如果你有过敏性鼻炎，可能会出现持续咳嗽、气短等症状，尤其是在过敏原暴露后。  3.胃食管反流病（GERD）：胃酸反流可以刺激喉咙，导致咳嗽、气短等症状，特别是在夜间或饭后。  4.支气管扩张症：这种疾病会导致支气管扩张，伴随慢性咳嗽和粘痰。  5.心脏病：某些心脏疾病，如心衰，也可能引起气短、咳嗽等症状。  如果你还没有做全面检查，建议与医生进一步沟通，可能需要进行肺功能测试、胸部X光、过敏测试等检查来帮助诊断病因。 | 4 | 3 | 4 | 4 | 4 | 4 | 4 |
| **Q3** | 怎么区分哮喘和其他疾病，比如慢性支气管炎或过敏性咳嗽？ | 区分哮喘、慢性支气管炎和过敏性咳嗽通常需要综合考虑症状、病史和检查结果。以下是一些关键的区分点：  1.哮喘：  症状：常见症状包括反复的喘息、气促、胸闷和咳嗽，这些症状通常在夜间或清晨加重。  诱因：受寒冷空气、过敏原（如尘螨、花粉）或运动等刺激时症状容易加剧。  体检和测试：肺功能测试（如FEV1、PEF）显示可逆性气流受限，支气管激发试验（如支气管扩张剂）后症状改善较明显。  2.慢性支气管炎：  症状：长期咳嗽和痰多，尤其是早晨咳痰明显。症状通常逐渐发展，持续至少三个月以上。  病史：长期吸烟或长期暴露于有害气体（如空气污染）是常见的危险因素。  体检和检查：胸部X光、肺功能测试等检查可能显示慢性气道炎症和气流受限，但症状不像哮喘那样有明显的可逆性。  3.过敏性咳嗽：  症状：以咳嗽为主，尤其是晚上或接触过敏原时（如灰尘、花粉、动物皮屑等）。  特点：没有明显的喘息和气促。咳嗽往往干咳或轻微咳痰。  病史：有过敏性鼻炎或其他过敏症状，如湿疹、结膜炎等。  此外，医生可能会根据临床症状进行详细检查，例如肺功能测试、过敏原检测、支气管镜检查等，以帮助准确诊断。  如果你怀疑自己有其中一种疾病，建议及时就医，做必要的检查和诊断，以便得到正确的治疗。 | 4 | 3 | 4 | 4 | 4 | 4 | 4 |
| **Q4** | 过敏性哮喘和支气管哮喘有什么不同？ | 过敏性哮喘和支气管哮喘是两种类型的哮喘，它们在发病机制和诱因上有所不同：  1.过敏性哮喘：  原因：过敏性哮喘主要由过敏反应引起。常见的过敏原包括花粉、尘螨、霉菌、宠物毛发等。这些过敏原进入呼吸道后，引发免疫系统过度反应，导致气道炎症和收缩。  症状：典型症状包括喘息、咳嗽、呼吸急促、胸闷等，通常与接触过敏原有关。  免疫机制：涉及IgE抗体介导的过敏反应，免疫系统识别这些过敏原并触发一系列反应，引起气道过敏性炎症。  2.支气管哮喘：  原因：支气管哮喘是一种慢性气道炎症性疾病，虽然过敏因素可以是一个诱因，但它也可能由非过敏因素（如感染、寒冷空气、运动、烟雾等）引发。  症状：与过敏性哮喘类似，包括喘息、咳嗽、呼吸急促等，但病因更加广泛。  免疫机制：支气管哮喘的炎症机制可能不完全依赖于过敏反应，虽然也存在免疫反应，但可能涉及其他炎症介质，如细胞因子和其他免疫细胞。  总结：过敏性哮喘是支气管哮喘的一种类型，过敏性哮喘主要由过敏原引起，而支气管哮喘的诱因更为多样。两者的症状相似，但发病机制有所不同。 | 4 | 4 | 4 | 4 | 4 | 4 | 4 |
| **Q5** | 如果我经常感觉喘不过气来，是否就是得了哮喘？还是有其他可能？ | 感觉喘不过气来可能与哮喘相关，但也有其他可能的原因。哮喘的典型症状包括喘息、胸闷、咳嗽和呼吸急促，通常在夜间或清晨更加严重。如果你已经被诊断为哮喘并且正在使用药物管理，那么喘不过气的感觉可能是由以下因素引起的：  1.哮喘控制不良：如果药物治疗没有得到充分控制，可能会导致症状加剧。  2.过敏源或诱因：接触到过敏原（如花粉、灰尘、宠物毛发等）或其他触发因素（如冷空气、烟雾等）可能导致呼吸困难。  3.上呼吸道感染：感冒或其他病毒性感染可能加重呼吸系统症状。  4.压力或焦虑：精神状态的改变，如压力或焦虑，可能引发类似哮喘的症状。  当然，也有其他非哮喘的可能性，例如：  慢性阻塞性肺疾病（CPD）：这是一种与吸烟有关的长期肺部疾病，常伴随呼吸急促。  过度换气症候群：紧张或焦虑时，呼吸模式不正常，可能导致喘不过气的感觉。  心脏问题：心脏疾病也可能导致类似哮喘的症状，如呼吸急促。  如果你经常感觉喘不过气来，建议你咨询医生进行详细的检查和诊断，找出症状的具体原因，并调整治疗方案。 | 4 | 3 | 4 | 4 | 4 | 4 | 4 |
| **Q6** | 我只有咳嗽不喘，医生说我得了哮喘，这样判断准确吗？ | 是的，哮喘的症状不仅仅包括喘息，咳嗽也是常见的症状之一，特别是在夜间或清晨。医生的诊断是根据你的症状、体检和其他检查结果来判断的。如果你只出现咳嗽而没有喘息，也有可能是哮喘的一种表现，特别是咳嗽型哮喘。咳嗽型哮喘常常在呼吸道受到刺激时表现为持续或间歇性的干咳。  如果你有任何疑虑或症状变化，建议与医生保持沟通，确保你的病情得到及时的管理和调整。 | 4 | 4 | 4 | 4 | 4 | 4 | 4 |
|  | **COVID-19 Related** |  |  |  |  |  |  |  |  |
| **Q1** | 我是哮喘患者，感染新冠的风险是否比普通人高？我该怎么保护自己？ | 是的，作为哮喘患者，感染新冠的风险可能比普通人更高。哮喘患者的气道已经存在一定的炎症和狭窄，感染新冠后可能会加重呼吸困难、气喘等症状，甚至可能引发哮喘急性发作。因此，保护自己非常重要。  以下是一些保护措施：  1.接种疫苗：接种新冠疫苗是减少重症风险的重要手段。根据医生建议，确保按时接种疫苗并接受加强针。  2.佩戴口罩：在人群密集的地方佩戴口罩，特别是N95口罩，能有效防止病毒传播。  3.保持社交距离：避免去人多的地方，减少与他人的接触，尤其是在公共场所和封闭环境中。  4.增强免疫力：保持健康的饮食、规律的作息，补充足够的维生素C、D等，保持身体的免疫力。  5.合理使用药物：继续按医生的指示服用哮喘药物，特别是控制药物，保持哮喘症状的稳定。如果感到不适或症状加重，请及时就医。  6.保持良好的手卫生：勤洗手，避免用手触摸面部，尤其是眼睛、鼻子和嘴巴，减少病毒通过接触传播的机会。  7.避免接触有症状的人：如果你身边的人出现感冒、发热等症状，尽量避免接触，减少被感染的风险。  8.监测症状：如果你感觉到任何呼吸困难、气喘或其他不寻常的症状，及时联系医生，做好必要的检查和治疗。  通过这些措施，可以在一定程度上降低感染新冠的风险，并减轻感染后可能带来的不适。 | 4 | 4 | 4 | 4 | 4 | 4 | 4 |
| **Q2** | 如果我得了新冠病毒，作为哮喘患者应该怎么处理？ | 如果你得了新冠病毒，作为哮喘患者，应该特别注意以下几个方面：  1.密切监测症状：新冠病毒可能会加重哮喘症状，如咳嗽、呼吸急促和喘息。务必密切监测这些症状，并记录下它们的变化。  2.继续按时使用哮喘药物：即使感染了新冠病毒，也不要停止使用你的常规哮喘药物（如吸入性皮质类固醇、支气管扩张剂等），除非医生指示停止。按时使用这些药物有助于控制哮喘症状，并减少因病毒引发的呼吸道炎症。  3.注意休息和保持水分：保持足够的休息，确保充足的睡眠，并多喝水以帮助身体对抗病毒。  4.保持社交距离和隔离：为了避免传染他人，尽量保持与他人的距离，并按医生的建议进行自我隔离。  5.避免诱发因素：如环境中的过敏原、空气污染等，可以加重哮喘症状，因此要尽量避免这些因素。  6.关注呼吸困难和体温变化：如果你感到呼吸急促或体温超过38°C，或者有明显的胸闷、持续的咳嗽等症状，应该立即联系医生或寻求紧急医疗帮助。  7.及时就医：如果你的哮喘症状加重，或者感到呼吸困难、胸痛等，及时就医，避免症状进一步恶化。  总之，作为哮喘患者，感染新冠时要特别谨慎，确保按时治疗并及时处理任何突发的健康问题。如果有任何不适，尽早联系医生进行咨询和治疗。 | 4 | 4 | 4 | 4 | 4 | 4 | 4 |
| **Q3** | 我应该接种新冠疫苗吗？对哮喘有什么影响吗？ | 接种新冠疫苗对大多数人来说是安全的，包括哮喘患者。事实上，哮喘患者接种新冠疫苗甚至是强烈推荐的，因为他们可能面临较高的感染风险和因新冠导致的严重疾病风险。  关于哮喘患者接种新冠疫苗的影响，以下是一些需要注意的要点：  1.疫苗安全性：新冠疫苗的临床试验表明，大多数哮喘患者可以安全接种。对哮喘的影响通常是微乎其微的。  2.疫苗副作用：就像其他人一样，哮喘患者可能会出现疫苗的常见副作用，如注射部位的疼痛、发热或疲劳。这些反应一般是短暂的，不会加重哮喘症状。  3.疫苗后过敏反应：虽然极少数人可能会对疫苗产生过敏反应，但过敏反应在哮喘患者中并不更常见。如果你对某些成分过敏（如聚乙二醇或其他疫苗成分），可以提前咨询医生。  4.控制好哮喘：接种疫苗前，确保哮喘得到良好的控制。如果你的哮喘症状不稳定，建议在接种前与医生讨论，确保最佳时机。  总之，大部分哮喘患者可以安全接种新冠疫苗，但始终最好咨询你的医生，尤其如果你有任何疑虑或健康状况不稳定时。 | 4 | 4 | 4 | 4 | 4 | 4 | 4 |
| **Q4** | 哮喘患者感染新冠后的症状会比正常人更严重吗？需要特别注意什么？ | 哮喘患者感染新冠后，症状可能比正常人更严重。因为哮喘本身就会影响呼吸道的健康，而新冠病毒（尤其是严重型）可能加重肺部症状，增加呼吸困难的风险。哮喘患者的气道可能已经敏感，感染后可能会导致更严重的呼吸道问题，例如咳嗽、气短、呼吸急促和胸闷等。  特别注意事项：  1.保持药物治疗：哮喘患者应继续按医嘱使用控制药物（如吸入性类固醇等），即使在感染期间，也不要擅自停药或改变剂量。  2.及时就医：如出现呼吸急促、持续性咳嗽、胸闷等加重症状，应及时联系医生，必要时寻求急诊治疗。  3.密切监测症状：定期监测呼吸情况，使用峰流速计检查肺功能，确保气道没有受限。  4.避免感染恶化：保持良好的卫生习惯，佩戴口罩，避免与感染者密切接触。  5.注意症状变化：如果感觉症状加重或有新的症状（如高烧、嗓子痛等），应考虑新冠感染的可能性，及时做核酸或抗原检测。  6.保持适当的氧气供应：如果出现呼吸困难，可考虑使用氧气治疗，尤其是有低氧血症风险的患者。  在感染新冠的情况下，哮喘患者需要更加小心，保持与医生的联系，以确保获得最佳的治疗方案。 | 4 | 4 | 4 | 4 | 4 | 4 | 4 |
|  | **Mental Health** |  |  |  |  |  |  |  |  |
| **Q1** | 我有哮喘，生活中总觉得很累，感到很无力，这正常吗？ | 哮喘患者由于气道受限和呼吸不畅，可能会感到比较疲劳和无力。长期的呼吸困难可能影响到氧气的摄取，导致身体缺氧，从而产生疲劳感。此外，如果哮喘症状控制不良，或是服药不够规律，疲劳感也可能加剧。  建议你定期复诊，确保哮喘得到适当的管理，并按照医生的指示正确使用药物。如果疲劳感持续或加重，最好与医生讨论，看看是否有其他因素或健康问题可能导致这些症状。保持良好的睡眠、均衡的饮食和适度的运动也有助于缓解疲劳感。 | 4 | 4 | 4 | 4 | 4 | 4 | 4 |
| **Q2** | 哮喘影响了我的心情，我常常焦虑，应该怎么办？ | 哮喘对身体和情绪的影响是可以理解的，尤其是当你感到呼吸困难时，可能会导致焦虑和压力。以下是一些可能帮助你缓解焦虑和改善情绪的方法：  1.呼吸练习：深呼吸练习可以帮助放松身体，减轻焦虑。你可以尝试腹式呼吸，即慢慢地通过鼻子吸气，感受腹部膨胀，再慢慢地通过嘴巴呼气。这样可以帮助你冷静下来。  2.冥想与放松技巧：冥想和放松技巧有助于减轻焦虑。你可以尝试静坐冥想，专注于当下的感受，或者使用放松音乐和引导冥想应用来帮助你放松。  3.运动：适度的运动有助于释放压力，并改善情绪。选择低强度的运动，如散步、瑜伽或游泳，这些都可以帮助你更好地管理哮喘和焦虑。  4.建立支持系统：和家人、朋友或专业人士沟通，表达你的感受，可能会让你感到更加宽慰。有时候，谈论你的焦虑和哮喘问题可以带来心理上的支持和安慰。  5.遵循治疗方案：确保按时服用哮喘药物，并与医生定期沟通，确保病情得到有效管理。控制哮喘可以减少焦虑带来的压力。  6.心理治疗：如果焦虑影响到你的日常生活，可以考虑寻求专业心理治疗，如认知行为疗法（CBT），这可以帮助你更好地应对情绪。  照顾好自己的身心健康，逐步找到适合自己的应对方法，如果焦虑持续或加重，建议咨询医生或心理专家。 | 4 | 4 | 4 | 4 | 4 | 4 | 4 |
| **Q3** | 哮喘让我总是担心会突然发作，是否有方法可以让我保持冷静？ | 哮喘的突发确实会让人感到不安，但有一些方法可以帮助你保持冷静并有效应对突发情况：  1.深呼吸练习：当你感到焦虑时，进行深呼吸练习可以帮助你冷静下来。尝试缓慢、深而长的呼吸，确保每次吸气和呼气都非常有意识，这有助于放松身体，减轻焦虑。  2.使用舒缓技巧：如冥想、瑜伽、或者听轻松的音乐等放松技巧，都能有效减轻压力，帮助你保持冷静。  3.随身携带药物：确保你总是带着你的哮喘药物，特别是救急药物。知道有药物在手可以让你感觉更有掌控感，减少突发时的恐惧。  4.学习应急应对：了解如何识别哮喘加重的早期症状，并提前准备好应急计划。掌握如何使用你的药物（如吸入器）以及何时寻求医疗帮助。  5.定期锻炼：适度的运动可以增强你的肺功能，减少哮喘发作的频率。确保你选择适合哮喘患者的锻炼方式，如游泳等。  6.保持与医生的沟通：定期与医生讨论你的哮喘状况，确保你正在使用最合适的治疗方案。这有助于你对自己情况的掌握，减少不确定性带来的焦虑。  通过这些方法，你可以在面对可能的哮喘发作时，保持冷静并及时应对，增强自信。 | 4 | 4 | 4 | 4 | 4 | 4 | 4 |
|  | **Lifestyle** |  |  |  |  |  |  |  |  |
| **Q1** | 我该怎么调整饮食，避免引发哮喘？ | 对于哮喘患者来说，饮食对控制病情至关重要。以下是一些建议，可以帮助你调整饮食，减少引发哮喘的风险：  1.避免过敏原食物：某些食物可能引发过敏反应，进而导致哮喘发作。常见的过敏原包括：  奶制品（如牛奶、奶酪）  坚果（如花生、杏仁）  贝类（如虾、蟹）  鸡蛋 如果你知道自己对某些食物过敏，尽量避免食用。  2.减少加工食品和高盐食物：加工食品、即食食品以及高盐食物可能会增加身体的炎症反应，加重哮喘症状。尽量选择新鲜、自然的食材，避免过多的盐分和人工添加剂。  3.增加抗炎食物的摄入：一些食物具有天然的抗炎作用，可以帮助减轻哮喘症状。常见的抗炎食物包括：  富含Ω-3脂肪酸的食物（如三文鱼、亚麻籽、核桃）  富含抗氧化物的食物（如蓝莓、樱桃、胡萝卜、绿叶蔬菜）  大蒜、姜等具有天然抗炎作用的食材。  4.适量摄入维生素D：有研究显示，低维生素D水平与哮喘发作频率增高有关。你可以通过阳光照射、食物（如鱼类、蛋黄、强化奶制品）或者补充剂来增加维生素D的摄入。  5.保持水分：保持充足的水分摄入有助于稀释呼吸道的粘液，缓解哮喘症状。尤其是在寒冷天气或者干燥环境中，更要注意多喝水。  6.避免引发胃食道反流的食物：胃食道反流可能加重哮喘症状，尤其是晚上。避免油腻、辛辣、巧克力、咖啡等食物。  每个人的哮喘触发因素可能不同，所以调整饮食时需要留心观察哪些食物会引起你的症状。如果有需要，可以咨询医生或营养师，定制适合你的饮食计划。 | 4 | 4 | 4 | 4 | 4 | 4 | 4 |
| **Q2** | 我的家里有宠物，会加重我的哮喘症状吗？ | 是的，宠物可能会加重哮喘症状。宠物的毛发、皮屑和唾液等可能会成为过敏源，引发过敏反应，进而刺激哮喘症状。特别是猫和狗，它们的皮屑和毛发会在空气中飘浮，容易被吸入，从而可能导致呼吸急促、咳嗽或喘息等症状。  如果你有哮喘，最好采取以下措施来减轻宠物对健康的影响：  1.保持宠物的清洁，定期洗澡和梳毛。  2.在家里设置宠物的专属区域，避免宠物进入卧室和其他活动频繁的地方。  3.定期清洁家里的空调、空气净化器和地面，减少宠物的毛发和皮屑积聚。  4.佩戴口罩或使用空气净化设备，减少空气中过敏源的浓度。  如果症状加重，建议咨询医生，可能需要调整哮喘的治疗方案。 | 4 | 4 | 4 | 4 | 4 | 4 | 4 |
| **Q3** | 我可以做运动吗？哪些运动对哮喘患者更好？ | 哮喘患者通常是可以做运动的，但需要注意选择适合的运动项目，并采取一些预防措施。运动有助于增强体力和呼吸功能，但某些剧烈运动可能会诱发哮喘症状。因此，以下是一些对哮喘患者较为适合的运动建议：  适合的运动：  1.游泳：水中的湿润空气有助于减少呼吸道的刺激，是哮喘患者推荐的运动。游泳可以增强心肺功能，并且运动时较少暴露在过敏源或空气污染物中。  2.散步和慢跑：适度的有氧运动如散步或慢跑有助于提升心肺健康，哮喘患者可以根据自身状况选择合适的强度，避免剧烈运动带来不适。  3.瑜伽：瑜伽有助于改善呼吸控制和放松，对增强肺功能及减轻压力非常有益。通过深呼吸和冥想练习，有助于管理哮喘症状。  4.骑自行车：适度的骑行对增强心肺耐力有帮助，但需要注意避免高强度骑行，尤其是在空气污染或过敏源较高的环境中。  5.力量训练：轻度或中等强度的力量训练有助于增强肌肉力量，改善整体健康，而不会过度刺激气道。  注意事项：  在运动前，建议提前使用哮喘药物（如吸入型激素或支气管扩张剂），以防止运动诱发哮喘症状。  避免在空气污染严重、过敏源高的日子进行户外运动。  运动时要保持适度，避免过度劳累。  运动结束后要慢慢放松，避免骤然停止活动。  总之，选择适合自己身体状况的运动并适度进行，能帮助你提高生活质量。 | 4 | 4 | 4 | 4 | 4 | 4 | 4 |
| **Q4** | 我体重比较重，能通过减肥来改善哮喘症状吗？ | 减肥可能有助于改善哮喘症状，尤其是在体重较重的人群中。过重的体重可能增加呼吸系统的负担，加剧哮喘症状。减轻体重可以减少胸部压力，使肺部功能得到改善，进而减少哮喘的发生和症状的严重程度。  然而，减肥并不能替代哮喘的常规治疗。您仍然需要按照医生的建议，使用哮喘药物来管理病情。建议您在减肥过程中，保持健康饮食、适度运动，并与医生保持联系，确保自己的哮喘症状在减肥过程中得到有效控制。  如果您有进一步的健康问题或减肥计划，建议与医生讨论，制定一个适合您身体状况的减肥计划。 | 4 | 4 | 4 | 4 | 4 | 4 | 4 |
| **Q5** | 作为哮喘患者，我平时生活中应该注意哪些事项？ | 作为哮喘患者，日常生活中需要特别注意一些事项来减少哮喘发作的风险，以下是一些建议：  1.避免过敏源：  尽量避免接触已知的过敏源，如花粉、尘螨、宠物毛发、霉菌等。  定期清洁家中，特别是床上用品和地毯，避免积尘。  保持空气清新，使用空气净化器。  2.保持环境湿度适中：  空气过于干燥或湿气过重都可能诱发哮喘，尽量保持适宜的湿度。  在冬季使用加湿器，夏季使用除湿机。  3.避免剧烈运动：  如果天气较冷或空气污染较严重时，避免户外剧烈运动。  可选择在室内进行运动，或适当调整运动强度。  4.使用药物：  定时按医生建议使用吸入药物或其他哮喘药物。  始终携带速效药物（如救急吸入器），以应对突发症状。  5.注意天气变化：  寒冷天气、湿气和空气污染较重的日子容易引发哮喘。出门时尽量穿戴防护，避免过度劳累。  6.控制体重和饮食：  保持健康的体重，避免肥胖，这有助于减少哮喘症状。  避免食用容易引起过敏或刺激性强的食物，如辛辣食物和高脂肪食物。  7.定期监测病情：  记录自己的症状变化，并定期与医生沟通调整治疗方案。  使用肺功能监测仪定期检测肺部健康。  通过这些措施，能够有效减轻哮喘症状，降低发作的频率，改善生活质量。 | 4 | 4 | 4 | 4 | 4 | 4 | 4 |
| **Q6** | 我在换季时常常会出现哮喘症状加重，除了增加药物治疗外，我还能做些什么来减轻症状？ | 换季时哮喘症状加重是很常见的情况，主要是因为气候变化、空气中的过敏原（如花粉、尘螨等）以及温度和湿度的波动都可能诱发哮喘发作。除了增加药物治疗，你还可以采取一些措施来减轻症状：  1. 避免过敏原：换季时，空气中往往有更多的过敏原，比如花粉、霉菌或尘螨。尽量避免在花粉高峰期外出，特别是在早晨或傍晚。另外，家中要定期清洁，使用空气净化器减少空气中的过敏原。  2. 保持室内空气湿润：干燥空气可能刺激呼吸道。使用加湿器增加空气湿度，避免空气过于干燥，尤其是寒冷干燥的季节。  3. 穿戴适当：避免穿过多衣物而导致汗液积聚，因为湿气也可能引发哮喘发作。注意保暖，但避免因过热或出汗而加重呼吸困难。  4. 定期运动：适量的运动可以提高身体的免疫力，帮助改善呼吸功能，但要注意选择适合的运动方式和强度，避免在空气污染较重或过敏原浓度较高的环境中运动。  5. 保持良好的生活习惯：确保充足的睡眠和合理的饮食，有助于增强免疫系统。尽量避免吸烟和二手烟，这些都会加剧哮喘症状。  6. 监测症状和峰值流量：定期监测自己的峰值流量，及时了解肺功能的变化。如果发现症状加重或用药效果不明显，及时就医调整治疗方案。  7. 避免剧烈天气变化：尽量避免在天气变化剧烈时外出，尤其是在气温突然下降时，这可能会刺激气道，引发哮喘症状。  这些措施可以在药物治疗之外帮助你更好地管理哮喘症状，但最重要的是定期跟医生沟通，确保你的治疗方案是最适合的。如果症状加重或者发生突发情况，一定要及时就医。 | 4 | 4 | 4 | 4 | 4 | 4 | 4 |
