# Supplementary material for: Assessing the Role of Large Language Models Between ChatGPT and DeepSeek in Asthma Education for Bilingual Individuals: Comparative Study
Source: JMIR Med Inform. 2025 Aug 13;13:e65365. doi: 10.2196/65365 (PMC12349887; doi:10.2196/65365)
Supplement: Multimedia Appendix 3 [file medinform-v13-e65365-s003.doc]

**Supplementary Table 3. DeepSeek’s Responses to Asthma-Related Inquiries in an English-Language Context**

| **NO.** | **Questions** | **Responses** | **Consistency** | **Completeness** | **Potential Bias** | **Reasoning Ability** | **Comprehension** | **Reliability** | **Safety** |
| --- | --- | --- | --- | --- | --- | --- | --- | --- | --- |
|  | **Basic Definitions** |  |  |  |  |  |  |  |  |
| **Q1** | I am 25 years old and have been experiencing coughing and chest tightness, especially in the mornings and at night. I took amoxicillin, but it had no effect. Could this be asthma? | I'm not a doctor, but I can provide some general information that might help you understand your symptoms. Coughing and chest tightness, especially in the morning and at night, can indeed be symptoms of asthma. Asthma is a chronic condition that causes inflammation and narrowing of the airways, leading to symptoms like wheezing, shortness of breath, chest tightness, and coughing. These symptoms are often worse at night or early in the morning.  However, these symptoms can also be caused by other conditions, such as:  Allergies: Allergic reactions to pollen, dust, or pet dander can cause similar symptoms.  Chronic Obstructive Pulmonary Disease (COPD): Although more common in older adults with a history of smoking, it can cause similar symptoms.  Gastroesophageal Reflux Disease (GERD): Acid reflux can irritate the airways and cause coughing, especially at night.  Postnasal Drip: Mucus from the sinuses dripping down the throat can cause coughing, particularly at night.  Viral Infections: Persistent cough after a viral infection (like a cold or flu) can last for weeks.  Since amoxicillin (an antibiotic) did not help, it suggests that your symptoms are unlikely to be caused by a bacterial infection, such as bronchitis or pneumonia. Antibiotics are ineffective against viral infections and non-infectious conditions like asthma.  What You Should Do:  See a Doctor: It’s important to consult a healthcare professional for an accurate diagnosis. They may perform tests like spirometry or a peak flow test to check for asthma or other lung conditions.  Monitor Symptoms: Keep track of when your symptoms occur, how severe they are, and if anything triggers them (e.g., exercise, cold air, allergens).  Avoid Triggers: If you suspect allergies or environmental factors are contributing, try to minimize exposure to potential triggers like dust, smoke, or pet dander.  Asthma is manageable with proper treatment, so if it is the cause of your symptoms, a doctor can help you develop a plan to control it. Don’t delay seeking medical advice, as untreated asthma or other conditions can worsen over time. | 4 | 4 | 4 | 4 | 4 | 4 | 4 |
| **Q2** | Is asthma a common condition? Could I have it? | Yes, asthma is a relatively common condition, affecting millions of people worldwide. It is a chronic respiratory disorder characterized by inflammation and narrowing of the airways, leading to symptoms such as wheezing, shortness of breath, chest tightness, and coughing. These symptoms can vary in severity and may be triggered by factors like allergens, exercise, cold air, or respiratory infections.  Could you have asthma?  While I can't diagnose you, here are some signs that might suggest you should consult a healthcare professional for evaluation:  Recurrent episodes of wheezing (a whistling sound when breathing).  Persistent coughing, especially at night or early in the morning.  Shortness of breath or difficulty breathing during physical activity or at rest.  Chest tightness or discomfort.  Symptoms that worsen in response to specific triggers (e.g., pollen, dust, pet dander, or cold air). If you experience any of these symptoms, it’s important to see a doctor. They can perform tests, such as spirometry or peak flow measurements, to determine if asthma or another condition is causing your symptoms. Early diagnosis and management can help control asthma effectively and improve your quality of life. | 4 | 4 | 4 | 4 | 4 | 4 | 4 |
| **Q3** | I suspect I have asthma. How can I get a diagnosis? | If you suspect you have asthma, it's important to seek a proper diagnosis from a healthcare professional. Here’s a step-by-step guide on how to get a diagnosis:  1. Recognize the Symptoms  Common asthma symptoms include:  Wheezing (a whistling sound when breathing)  Shortness of breath  Chest tightness or pain  Coughing, especially at night or early morning  Symptoms may worsen with exercise, allergies, cold air, or respiratory infections.  2. Visit Your Healthcare Provider  Schedule an appointment with your primary care doctor, or consider seeing an allergist or pulmonologist (lung specialist) if your symptoms are severe or persistent.  Be prepared to discuss:  Your symptoms (frequency, triggers, and severity)  Your medical history, including family history of asthma or allergies  Any environmental or occupational exposures that might contribute to your symptoms.  3. Undergo a Physical Exam  Your doctor will listen to your breathing and check for signs of asthma or other conditions.  4. Diagnostic Tests  Your doctor may order one or more of the following tests to confirm asthma:  Spirometry: Measures how much air you can exhale and how quickly. It helps assess lung function.  Peak Flow Test: Measures how hard you can breathe out. Low peak flow readings can indicate asthma.  Bronchoprovocation Test: Checks how your airways react to triggers (e.g., exercise, cold air, or a substance like methacholine).  FeNO Test: Measures nitric oxide levels in your breath, which can indicate airway inflammation.  Allergy Testing: To determine if allergies are triggering your symptoms.  Chest X-ray or CT Scan: To rule out other conditions like infections or structural issues.  5. Review the Results  Your doctor will analyze the test results and determine if your symptoms are consistent with asthma or another condition (e.g., COPD, bronchitis, or GERD).  6. Develop a Treatment Plan  If diagnosed with asthma, your doctor will create a personalized asthma action plan, which may include:  Medications (e.g., inhalers, corticosteroids)  Lifestyle changes (e.g., avoiding triggers, quitting smoking)  Regular monitoring of your symptoms and lung function.  7. Follow Up  Asthma is a chronic condition that requires ongoing management. Schedule regular check-ups to monitor your condition and adjust your treatment plan as needed.  When to Seek Immediate Help:  If you experience severe symptoms like extreme difficulty breathing, bluish lips or face, or inability to speak, seek emergency medical care immediately.  By working with your healthcare provider, you can get an accurate diagnosis and effective treatment to manage your symptoms and improve your quality of life. | 4 | 4 | 4 | 4 | 4 | 4 | 4 |
| **Q4** | What tests are needed to diagnose asthma? Are they blood tests or something else? | Diagnosing asthma typically involves a combination of medical history, physical examination, and specific tests to assess lung function. Blood tests are not the primary method for diagnosing asthma, but they may be used to rule out other conditions or identify allergies. Here are the main tests used to diagnose asthma:  1. Medical History and Physical Examination  Your doctor will ask about symptoms (e.g., wheezing, shortness of breath, coughing, chest tightness), their frequency, and potential triggers (e.g., allergens, exercise, cold air).  A physical exam may include listening to your lungs with a stethoscope for wheezing or other abnormal sounds.  2. Lung Function Tests  These are the most important tests for diagnosing asthma:  Spirometry: Measures how much air you can exhale and how quickly. It assesses airflow obstruction, which is a key feature of asthma.  Peak Expiratory Flow (PEF) Test: Measures how fast you can blow air out of your lungs using a peak flow meter. Low readings may indicate asthma.  3. Bronchoprovocation Tests  These tests evaluate how your airways react to triggers:  Methacholine Challenge Test: Involves inhaling a substance that narrows the airways in people with asthma. A positive result suggests asthma.  Exercise Challenge Test: Assesses whether exercise triggers asthma symptoms.  4. FeNO Test (Fractional Exhaled Nitric Oxide)  Measures the level of nitric oxide in your breath, which can indicate inflammation in the airways, a common feature of asthma.  5. Allergy Testing  While not specific to asthma, allergy tests (skin prick tests or blood tests for specific IgE antibodies) can identify allergens that may trigger asthma symptoms.  6. Chest X-ray or CT Scan  These imaging tests are not used to diagnose asthma but may be ordered to rule out other conditions (e.g., pneumonia, structural abnormalities) that could cause similar symptoms.  7. Blood Tests  Blood tests are not used to diagnose asthma directly but may be done to check for signs of infection, inflammation, or allergies.  Summary  The diagnosis of asthma relies heavily on lung function tests (spirometry, PEF) and bronchoprovocation tests. Blood tests and imaging are typically used to rule out other conditions. If you suspect you have asthma, consult a healthcare provider for a proper evaluation. | 4 | 4 | 4 | 4 | 4 | 4 | 4 |
| **Q5** | I've been coughing, wheezing, and feeling tightness in my chest recently. Do these symptoms mean I have asthma? | I'm not a doctor, but I can provide some general information. Coughing, wheezing, and chest tightness are common symptoms of asthma, but they can also be caused by other conditions, such as respiratory infections, allergies, chronic obstructive pulmonary disease (COPD), or even acid reflux.  To determine if these symptoms are due to asthma or another condition, it's important to consult a healthcare professional. They may perform tests, such as spirometry or a peak flow test, to assess your lung function and help diagnose the issue. If you're experiencing severe difficulty breathing, seek medical attention immediately.  In the meantime, try to avoid potential triggers like smoke, allergens, or cold air, and monitor your symptoms. Keeping a symptom diary can also help your doctor make an accurate diagnosis. | 4 | 4 | 4 | 4 | 4 | 4 | 4 |
| **Q6** | I have asthma. Why do doctors say it might be related to genetics? | Asthma is a complex condition influenced by both genetic and environmental factors. Here’s why doctors might say your asthma could be related to genetics:  Family History: Asthma often runs in families. If one or both of your parents have asthma, allergies, or other related conditions (like eczema), you are more likely to develop asthma. This suggests a genetic predisposition.  Genetic Mutations: Research has identified specific genes associated with asthma. These genes can affect how your immune system responds to allergens, how your airways react to irritants, or how your body manages inflammation. For example, variations in genes like ADAM33, ORMDL3, and IL33 have been linked to asthma.  Immune System Regulation: Some genetic traits can make your immune system more sensitive to environmental triggers (like pollen, dust, or pollution), leading to airway inflammation and asthma symptoms.  Ethnic and Racial Factors: Certain populations have higher rates of asthma, which may be partly due to genetic differences. For example, asthma is more common in people of African or Puerto Rican descent.  Gene-Environment Interaction: While genetics play a role, environmental factors (like exposure to allergens, pollution, or respiratory infections) often trigger asthma in genetically predisposed individuals. Your genes may make you more susceptible to these triggers.  While genetics can increase your risk, they don’t guarantee you’ll develop asthma. Doctors consider both your genetic background and environmental exposures when diagnosing and managing asthma. If you have a family history of asthma, it’s important to monitor your symptoms and avoid known triggers. | 4 | 4 | 4 | 4 | 4 | 4 | 4 |
| **Q7** | The doctor said I have asthma. Why are my allergy tests negative? | It's not uncommon for people with asthma to have negative allergy tests, and there are a few reasons why this might happen:  Non-Allergic Asthma: Not all asthma is triggered by allergies. Some people have non-allergic asthma, which can be caused by factors like exercise, cold air, stress, smoke, or respiratory infections. If your asthma isn't triggered by allergens, your allergy tests may come back negative.  Allergy Test Limitations: Allergy tests (like skin prick tests or blood tests for specific IgE) are designed to detect reactions to common allergens, such as pollen, dust mites, or pet dander. However, they may not cover all possible allergens, or you might be sensitive to substances that aren't tested.  Localized Allergic Reactions: Some people have allergic reactions that are localized to the airways (like the lungs) but don't show up on systemic allergy tests. This is sometimes seen in allergic bronchopulmonary aspergillosis (ABPA) or other conditions where the immune response is confined to the lungs.  Delayed or Non-IgE Reactions: Allergy tests typically look for IgE-mediated reactions, which are immediate hypersensitivity responses. However, some allergic reactions are delayed or involve other parts of the immune system (e.g., T-cell-mediated responses), which won't be detected by standard allergy tests.  Other Triggers: Asthma can also be triggered by non-allergic irritants, such as air pollution, strong odors, or chemical fumes. These triggers won't show up on allergy tests.  Testing Timing or Accuracy: If you were tested during a time when you weren't exposed to the allergen or if the test wasn't performed correctly, it could lead to a false negative result.  If your allergy tests are negative but you still have asthma symptoms, your doctor might explore other triggers or types of asthma. They may also consider additional tests or treatments, such as lung function tests (spirometry) or a trial of asthma medications, to better manage your condition. Always discuss your concerns with your healthcare provider to ensure you're getting the most accurate diagnosis and treatment. | 4 | 4 | 4 | 4 | 4 | 4 | 4 |
|  | **Clinical Features** |  |  |  |  |  |  |  |  |
| **Q1** | What are the typical symptoms of asthma? How can I tell if I have it? | Asthma is a chronic respiratory condition characterized by inflammation and narrowing of the airways, leading to difficulty in breathing. The symptoms can vary in severity and frequency from person to person. Here are the typical symptoms of asthma:  Wheezing: A whistling or squeaky sound when you breathe, especially during exhalation.  Shortness of Breath: Feeling out of breath or unable to get enough air.  Chest Tightness: A sensation of pressure or constriction in the chest.  Coughing: Often worse at night or early in the morning, and may be persistent or intermittent.  How to Tell if You Have Asthma  If you experience any of the above symptoms, especially if they recur or worsen in response to certain triggers (such as allergens, cold air, exercise, or respiratory infections), it’s important to consult a healthcare provider. They can perform diagnostic tests to confirm asthma, such as:  Spirometry: A lung function test that measures how much air you can exhale and how quickly.  Peak Flow Test: Measures how fast you can blow air out of your lungs.  Bronchoprovocation Test: Assesses how your airways react to specific triggers.  Medical History and Physical Exam: Your doctor will review your symptoms, family history, and overall health.  When to Seek Immediate Medical Attention  Seek emergency care if you experience:  Severe shortness of breath or wheezing.  Symptoms that don’t improve after using a rescue inhaler.  Bluish lips or face (cyanosis), which indicates a lack of oxygen.  If you suspect you have asthma, don’t self-diagnose. A healthcare professional can provide an accurate diagnosis and recommend appropriate treatment. | 4 | 4 | 4 | 4 | 4 | 4 | 4 |
| **Q2** | Besides coughing and wheezing, are there other less common symptoms of asthma? | Yes, besides coughing and wheezing, asthma can present with several less common symptoms. These may include:  Shortness of breath (dyspnea): Difficulty breathing or a feeling of not being able to get enough air, which may occur even during mild physical activity or at rest.  Chest tightness or pain: A sensation of pressure or discomfort in the chest, which can sometimes be mistaken for heart-related issues.  Rapid breathing (tachypnea): Breathing faster than normal, often as a response to the body's attempt to get more oxygen.  Fatigue: Feeling unusually tired, especially after an asthma episode or due to disrupted sleep from nighttime symptoms.  Difficulty sleeping: Nighttime asthma symptoms, such as coughing or shortness of breath, can interfere with sleep.  Anxiety or panic: The struggle to breathe can lead to feelings of anxiety or panic, which may worsen breathing difficulties.  Sighing or frequent yawning: Some people with asthma may sigh or yawn more often as a way to try to get more air.  Difficulty speaking: Severe asthma attacks can make it hard to speak in full sentences due to breathlessness.  Nasal flaring: The nostrils may widen when breathing in, particularly in children, as a sign of respiratory distress.  Cyanosis: In severe cases, a bluish tint to the lips, face, or fingernails may occur due to a lack of oxygen in the blood.  These symptoms can vary in intensity and may not always be immediately recognized as related to asthma, especially in mild or atypical cases. If you or someone else experiences any of these symptoms, especially if they worsen or persist, it’s important to seek medical advice for proper evaluation and management. | 4 | 4 | 4 | 4 | 4 | 4 | 4 |
| **Q3** | Can asthma cause other physical problems, like headaches or loss of appetite? | Asthma primarily affects the respiratory system, causing symptoms such as wheezing, shortness of breath, chest tightness, and coughing. However, it can indirectly lead to other physical issues, including headaches or loss of appetite, due to the stress and strain it places on the body. Here's how:  Headaches:  Asthma can sometimes lead to headaches, particularly if breathing difficulties result in low oxygen levels (hypoxia) or high carbon dioxide levels (hypercapnia) in the blood.  Frequent use of rescue inhalers (e.g., albuterol) or other asthma medications may also contribute to headaches as a side effect.  Poor sleep due to nighttime asthma symptoms can trigger tension headaches or migraines.  Loss of Appetite:  Severe asthma attacks or chronic asthma can cause fatigue and difficulty breathing, which may reduce a person's desire to eat.  Some asthma medications, particularly oral corticosteroids (e.g., prednisone), can cause stomach irritation or nausea, leading to a decreased appetite.  The stress and anxiety associated with managing asthma can also affect appetite.  Other Indirect Effects:  Fatigue: Poor sleep due to nighttime asthma symptoms or the effort required to breathe can lead to chronic fatigue.  Weight Changes: Long-term use of corticosteroids can cause weight gain, while loss of appetite or difficulty eating during flare-ups might lead to weight loss.  Anxiety and Depression: The chronic nature of asthma and its impact on daily life can contribute to mental health issues, which may further affect physical well-being.  If you or someone you know is experiencing significant headaches, loss of appetite, or other physical problems related to asthma, it’s important to consult a healthcare provider. Proper asthma management, including medication adjustments and lifestyle changes, can help alleviate these secondary issues. | 4 | 4 | 4 | 4 | 4 | 4 | 4 |
| **Q4** | What would an asthma attack look like? What should I do during an attack? | An asthma attack occurs when the airways become inflamed and constricted, making it difficult to breathe. Symptoms can vary in severity but often include:  Signs of an Asthma Attack:  Shortness of breath: Difficulty breathing or feeling out of breath.  Wheezing: A whistling or squeaky sound when breathing, especially during exhalation.  Coughing: Persistent coughing, which may worsen at night or during physical activity.  Chest tightness: A feeling of pressure or squeezing in the chest.  Rapid breathing: Breathing faster than normal.  Difficulty speaking: Struggling to complete sentences due to breathlessness.  Retractions: The skin around the ribs or neck may pull in with each breath.  Pale or sweaty face: A sign of distress.  What to Do During an Asthma Attack:  Stay calm: Panic can worsen symptoms. Try to remain as calm as possible.  Use a rescue inhaler: If you have a prescribed quick-relief inhaler (e.g., albuterol), use it as directed. Typically, this involves taking 1-2 puffs and waiting a few minutes to see if symptoms improve.  Sit upright: Sit in a comfortable position to help open your airways. Avoid lying down.  Breathe slowly and deeply: Focus on slow, steady breaths to help control your breathing.  Remove triggers: If possible, move away from any known triggers (e.g., smoke, dust, or allergens).  Monitor symptoms: If symptoms do not improve after using the inhaler, or if they worsen, seek emergency medical help immediately.  Call for help: If the attack is severe, or if you experience bluish lips/fingernails, extreme difficulty breathing, or confusion, call emergency services (e.g., 911) right away.  Prevention Tips:  Follow your asthma action plan if you have one.  Avoid known triggers (e.g., pollen, pet dander, smoke).  Take prescribed controller medications regularly, even if you feel fine.  Keep your rescue inhaler with you at all times.  If you or someone else is experiencing a severe asthma attack, do not delay seeking medical attention. Asthma attacks can be life-threatening if not treated promptly. | 4 | 4 | 4 | 4 | 4 | 4 | 4 |
| **Q5** | Why do I have no issues during the day, but start coughing and feeling tight at night? Is this characteristic of asthma? | Nighttime coughing and chest tightness can indeed be characteristic of asthma, as asthma symptoms often worsen at night due to natural changes in the body. Here are some reasons why this might happen:  Circadian Rhythm: Hormonal changes at night, such as lower levels of cortisol (an anti-inflammatory hormone), can increase airway inflammation and sensitivity.  Increased Mucus Production: Lying down can cause mucus to pool in the airways, triggering coughing.  Cooler Air: Cooler nighttime air can irritate the airways, especially if you have asthma.  Allergens in the Bedroom: Dust mites, pet dander, or mold in your bedding or bedroom can worsen symptoms at night.  Postnasal Drip: Lying down can cause mucus from the sinuses to drip into the throat, irritating the airways.  Gastroesophageal Reflux Disease (GERD): Acid reflux, which can worsen when lying down, may trigger coughing and chest tightness.  If these symptoms persist or worsen, it’s important to consult a healthcare provider. They may perform tests, such as spirometry, to determine if asthma or another condition is the cause. If asthma is diagnosed, treatments like inhalers or lifestyle changes can help manage symptoms. | 4 | 4 | 4 | 4 | 4 | 4 | 4 |
| **Q6** | I often feel tightness in my chest. Could this be a sign of an asthma attack? | Chest tightness can indeed be a symptom of an asthma attack, but it can also be associated with other conditions, such as anxiety, acid reflux, or heart issues. If you have asthma, chest tightness may occur alongside other symptoms like wheezing, shortness of breath, coughing, or difficulty breathing.  However, since chest tightness can also indicate serious conditions like a heart problem, it's important to seek medical attention if:  The tightness is severe or worsening.  You experience pain radiating to your arm, neck, or jaw.  You have difficulty breathing, dizziness, or sweating.  You have a history of heart disease or risk factors (e.g., high blood pressure, high cholesterol).  If you suspect an asthma attack, use your rescue inhaler (if prescribed) and follow your asthma action plan. If symptoms don’t improve or worsen, seek emergency care. Always consult a healthcare professional for a proper evaluation and diagnosis. | 4 | 4 | 4 | 4 | 4 | 4 | 4 |
|  | **Treatment** |  |  |  |  |  |  |  |  |
| **Q1** | I suspect I have asthma. Can I use any medications to relieve the symptoms before seeing a doctor? | I'm not a doctor, but I can provide some general information. If you suspect you have asthma or are experiencing symptoms like wheezing, shortness of breath, chest tightness, or coughing, it's important to consult a healthcare professional for a proper diagnosis and treatment plan. However, here are a few steps you might consider while waiting to see a doctor:  Avoid Triggers: Identify and avoid potential asthma triggers such as smoke, allergens, cold air, or strong odors.  Over-the-Counter (OTC) Options: There are limited OTC options for asthma relief. Primatene Mist (epinephrine) is an OTC inhaler, but it should be used with caution and only as a temporary measure, as it may have side effects and is not suitable for everyone.  Breathing Techniques: Practice slow, controlled breathing to help manage symptoms.  Monitor Symptoms: Keep track of your symptoms and their severity to share with your doctor.  However, if you experience severe symptoms such as extreme difficulty breathing, rapid worsening of symptoms, or bluish lips or face, seek emergency medical care immediately. Asthma can be serious, and proper medical evaluation and treatment are essential. | 4 | 4 | 4 | 4 | 4 | 4 | 4 |
| **Q2** | I’ve been diagnosed with asthma. What medication should I take? | I'm not a doctor, but I can provide general information. Asthma treatment typically involves two main types of medications:  Quick-Relief Medications (Rescue Inhalers): These are used during an asthma attack to quickly open up the airways and relieve symptoms. Common options include:  Short-acting beta-agonists (SABAs): Albuterol (e.g., Ventolin, ProAir) or levalbuterol (Xopenex).  Long-Term Control Medications: These are taken daily to prevent symptoms and reduce inflammation in the airways. Examples include:  Inhaled corticosteroids (ICS): Fluticasone (Flovent), budesonide (Pulmicort), or beclomethasone (Qvar).  Long-acting beta-agonists (LABAs): Often combined with ICS, such as fluticasone/salmeterol (Advair) or budesonide/formoterol (Symbicort).  Leukotriene modifiers: Montelukast (Singulair).  Biologics: For severe asthma, such as omalizumab (Xolair) or mepolizumab (Nucala).  Your doctor will determine the best treatment plan based on the severity of your asthma and your specific needs. Always follow your healthcare provider's instructions and discuss any concerns with them. If you experience severe symptoms like difficulty breathing, seek medical attention immediately. | 4 | 4 | 4 | 4 | 4 | 4 | 4 |
| **Q3** | The doctor prescribed me budesonide. Can you tell me what side effects I might experience? | Budesonide is a corticosteroid used to treat various inflammatory conditions, such as asthma, COPD, allergies, and certain gastrointestinal disorders (e.g., Crohn's disease). While it is generally well-tolerated, like all medications, it can cause side effects. The likelihood and severity of side effects depend on the form of budesonide (inhaled, oral, nasal spray, etc.) and the dose. Here are some possible side effects:  Common Side Effects:  Inhaled Budesonide (for asthma/COPD):  Sore throat or hoarseness  Cough  Dry mouth  Oral thrush (a fungal infection in the mouth; rinsing your mouth after use can help prevent this)  Nasal Spray (for allergies):  Nasal irritation or dryness  Nosebleeds  Sneezing  Headache  Oral Budesonide (for gastrointestinal conditions):  Upset stomach or nausea  Headache  Fatigue  Acne or skin changes  Less Common but Serious Side Effects:  Adrenal suppression (reduced function of the adrenal glands, especially with long-term or high-dose use)  Increased risk of infections (due to immune system suppression)  Vision problems (e.g., cataracts or glaucoma with long-term use)  Bone thinning (osteoporosis) with prolonged use  Mood changes (e.g., anxiety, depression, or irritability)  High blood pressure or fluid retention  Weight gain (more common with systemic corticosteroids but possible with budesonide)  Rare but Severe Side Effects:  Allergic reactions (e.g., rash, itching, swelling, severe dizziness, or trouble breathing)  Severe fatigue or weakness (could indicate adrenal insufficiency)  Unusual bruising or bleeding  What to Do:  If you experience mild side effects, they may resolve as your body adjusts to the medication. However, if they persist or worsen, contact your doctor.  For serious side effects (e.g., signs of infection, vision changes, or severe mood changes), seek medical attention immediately.  Always use budesonide as prescribed and do not stop taking it abruptly without consulting your doctor, as this could lead to withdrawal symptoms or a flare-up of your condition.  If you have concerns about side effects or how to use the medication, discuss them with your doctor or pharmacist. They can provide guidance tailored to your specific situation. | 4 | 4 | 4 | 4 | 4 | 4 | 4 |
| **Q4** | After using budesonide-formoterol, my heart rate increased, and I felt uncomfortable. Is this a normal reaction? | An increased heart rate and feelings of discomfort after using budesonide-formoterol (a combination inhaler containing a corticosteroid and a long-acting beta-agonist) can occur in some individuals, but it is not considered a "normal" or expected reaction for everyone. Here's what you should know:  Possible Side Effects: Formoterol, the long-acting beta-agonist (LABA) in the combination, can sometimes cause side effects such as an increased heart rate (tachycardia), palpitations, tremors, or nervousness. These effects are due to the medication's action on beta receptors in the body, which can stimulate the heart and nervous system.  Individual Sensitivity: Some people are more sensitive to the effects of beta-agonists, and these side effects may be more pronounced in them. If the symptoms are mild and temporary, they may not be a cause for concern. However, if they persist or worsen, you should consult your healthcare provider.  When to Seek Medical Attention: If the increased heart rate is severe, accompanied by chest pain, dizziness, or difficulty breathing, or if you feel extremely unwell, seek medical attention immediately. These could be signs of a more serious reaction or an underlying condition.  Review Your Technique: Ensure you are using the inhaler correctly. Improper use can lead to excessive medication delivery, increasing the likelihood of side effects.  Discuss with Your Doctor: If the side effects are bothersome or persistent, contact your healthcare provider. They may adjust your dosage, switch you to a different medication, or investigate other potential causes of your symptoms.  In summary, while an increased heart rate and discomfort can occur after using budesonide-formoterol, it is important to monitor your symptoms and consult your doctor if they persist or worsen. Do not stop or change your medication without medical advice. | 4 | 4 | 4 | 4 | 4 | 4 | 4 |
| **Q5** | My father also has asthma. After using salmeterol/fluticasone, he had a sore throat and hoarseness. Are these side effects of the medication? | Yes, sore throat and hoarseness are known side effects of salmeterol/fluticasone, which is a combination medication containing a long-acting beta-agonist (salmeterol) and an inhaled corticosteroid (fluticasone). These side effects are often related to the corticosteroid component, which can cause irritation or fungal infections (like oral thrush) in the throat and mouth.  To minimize these side effects, your father can try the following:  Rinse his mouth with water and spit it out after each use of the inhaler. This helps remove any residual medication from the mouth and throat.  Use a spacer device if he is using a metered-dose inhaler (MDI). This can help reduce the amount of medication that settles in the throat.  Practice good inhaler technique to ensure the medication reaches the lungs effectively.  If the symptoms persist or worsen, consult his healthcare provider. They may recommend adjusting the dose, switching medications, or treating any potential fungal infection.  If your father experiences severe throat irritation, difficulty swallowing, or signs of an allergic reaction (e.g., swelling, rash, or breathing difficulties), he should seek medical attention promptly. | 4 | 4 | 4 | 4 | 4 | 4 | 4 |
| **Q6** | Are there any new treatments that can help reduce frequent asthma attacks? | Yes, there have been several advancements in the treatment of asthma that can help reduce frequent asthma attacks. These treatments target different aspects of asthma management, including inflammation, bronchoconstriction, and underlying immune responses. Here are some of the newer treatments:  1. Biologic Therapies (Monoclonal Antibodies)  Biologic therapies are designed to target specific molecules or pathways involved in the inflammatory process of asthma. They are typically used for severe asthma that is not well-controlled with standard treatments. Examples include:  Anti-IL-5 therapies (e.g., mepolizumab, reslizumab, benralizumab): These target interleukin-5, a key player in eosinophilic inflammation.  Anti-IgE therapy (omalizumab): This targets immunoglobulin E (IgE), which is involved in allergic asthma.  Anti-IL-4/IL-13 therapy (dupilumab): This targets interleukins 4 and 13, which are involved in type 2 inflammation.  2. Bronchial Thermoplasty  This is a non-drug procedure for severe asthma. It involves delivering controlled thermal energy to the airway walls to reduce smooth muscle mass, thereby decreasing the ability of the airways to constrict. This can lead to fewer asthma attacks and improved quality of life.  3. Long-Acting Muscarinic Antagonists (LAMAs)  LAMAs, such as tiotropium, are traditionally used for COPD but have been approved as add-on therapy for asthma. They help relax the airway muscles and improve airflow.  4. Inhaled Corticosteroid (ICS) and Long-Acting Beta-Agonist (LABA) Combinations  Newer formulations and delivery devices for ICS/LABA combinations (e.g., fluticasone furoate/vilanterol, budesonide/formoterol) have improved efficacy and convenience, helping to better control asthma symptoms and reduce exacerbations.  5. Smart Inhalers  These are inhalers equipped with Bluetooth technology to track usage and remind patients to take their medication. Improved adherence to maintenance therapy can reduce the frequency of asthma attacks.  6. Tezepelumab  A newer biologic therapy, tezepelumab, targets thymic stromal lymphopoietin (TSLP), an upstream cytokine involved in the inflammatory cascade of asthma. It has shown promise in reducing exacerbations in patients with severe asthma.  7. Personalized Medicine  Advances in genetic and biomarker testing allow for more personalized treatment plans. For example, identifying specific biomarkers (e.g., eosinophils, IgE levels) can help tailor biologic therapies to individual patients.  8. Allergen Immunotherapy  For patients with allergic asthma, allergen immunotherapy (allergy shots or sublingual tablets) can help desensitize the immune system to specific allergens, potentially reducing asthma attacks triggered by allergies.  9. Novel Anti-Inflammatory Agents  Research is ongoing into new anti-inflammatory drugs, such as CRTH2 antagonists and JAK inhibitors, which may offer additional options for asthma control in the future.  10. Lifestyle and Environmental Modifications  While not a "treatment" in the traditional sense, addressing triggers such as air pollution, smoking, obesity, and stress can significantly reduce asthma attacks. New technologies, like air purifiers and wearable devices, can help monitor and mitigate these factors.  Conclusion  If you or someone you know is experiencing frequent asthma attacks, it’s important to consult a healthcare provider. They can assess whether these newer treatments might be appropriate and develop a tailored asthma action plan. Always follow medical advice and continue using prescribed medications as directed. | 4 | 4 | 4 | 4 | 4 | 4 | 4 |
| **Q7** | What’s the difference between inhaled and oral medications for asthma? Which is more effective? | Asthma medications can be administered via two primary routes: inhaled and oral. Each has distinct characteristics, advantages, and limitations. Here's a breakdown of the differences and their effectiveness:  1. Inhaled Medications  Delivery Method: These medications are delivered directly to the lungs via inhalers or nebulizers.  Types:  Quick-Relief Medications (e.g., short-acting beta-agonists like albuterol): Provide rapid relief during an asthma attack by relaxing the muscles around the airways.  Long-Term Control Medications (e.g., inhaled corticosteroids, long-acting beta-agonists): Used daily to reduce inflammation and prevent symptoms.  Advantages:  Direct delivery to the lungs, resulting in faster action.  Lower doses are required compared to oral medications, reducing the risk of systemic side effects.  Highly effective for both acute relief and long-term management.  Disadvantages:  Proper technique is required for effective delivery, which can be challenging for some patients.  May cause localized side effects like oral thrush or hoarseness (can be minimized with spacers or rinsing the mouth after use).  2. Oral Medications  Delivery Method: Taken by mouth in the form of tablets, capsules, or liquids.  Types:  Systemic Corticosteroids (e.g., prednisone): Used for short-term treatment of severe asthma exacerbations.  Leukotriene Modifiers (e.g., montelukast): Used for long-term control, particularly in mild asthma or allergic asthma.  Theophylline: A less commonly used long-term control medication.  Advantages:  Easier to administer, especially for children or those who struggle with inhaler techniques.  Useful for severe asthma or exacerbations when inhaled medications are insufficient.  Disadvantages:  Higher risk of systemic side effects (e.g., weight gain, mood changes, osteoporosis with long-term corticosteroid use).  Slower onset of action compared to inhaled medications.  Which is More Effective?  Inhaled medications are generally considered more effective for routine asthma management because they deliver the medication directly to the lungs, providing targeted relief with fewer systemic side effects. They are the first-line treatment for both acute symptoms and long-term control.  Oral medications are typically reserved for:  Severe asthma exacerbations (e.g., oral corticosteroids).  Patients who cannot use inhaled medications effectively.  Specific cases where leukotriene modifiers are beneficial (e.g., allergic asthma).  Conclusion:  Inhaled medications are preferred for most asthma patients due to their efficacy, targeted action, and lower risk of side effects. Oral medications play a complementary role, particularly in severe cases or when inhaled therapies are not feasible. The choice of medication depends on the severity of asthma, patient preference, and individual response to treatment. Always consult a healthcare provider for personalized recommendations. | 4 | 4 | 4 | 4 | 4 | 4 | 4 |
| **Q8** | I’m using asthma medication, but I forgot to take it today. What should I do to make up for it? | If you forget to take your asthma medication, here’s what you should do:  Check the type of medication:  Rescue inhaler (e.g., albuterol): Use it immediately if you’re experiencing symptoms like wheezing, shortness of breath, or coughing. This is for quick relief and doesn’t require a "make-up" dose.  Maintenance medication (e.g., inhaled corticosteroids or long-acting bronchodilators): Take the missed dose as soon as you remember, unless it’s almost time for your next dose. In that case, skip the missed dose and continue with your regular schedule. Do not double up on doses.  Monitor your symptoms: If you feel any asthma symptoms (e.g., tightness in the chest, difficulty breathing), use your rescue inhaler as needed.  Set a reminder: To avoid missing doses in the future, consider setting an alarm or using a medication reminder app.  Consult your doctor or pharmacist: If you’re unsure about what to do or if you frequently forget your medication, reach out to your healthcare provider for guidance. They may adjust your treatment plan or provide additional strategies.  Always follow your prescribed treatment plan and never make changes without consulting your healthcare provider. | 4 | 4 | 4 | 4 | 4 | 4 | 4 |
| **Q9** | If I don't want to rely on medication forever, can I manage asthma with diet or exercise? | While diet and exercise can play a supportive role in managing asthma, they are not substitutes for medication, especially in moderate to severe cases. Asthma is a chronic condition that often requires medical treatment to control inflammation and prevent symptoms. However, lifestyle changes can complement your treatment plan and potentially reduce the frequency or severity of symptoms. Here's how:  Diet  Anti-inflammatory foods: A diet rich in fruits, vegetables, whole grains, nuts, and seeds can help reduce inflammation in the body, which may benefit asthma management.  Omega-3 fatty acids: Found in fatty fish (like salmon), flaxseeds, and walnuts, omega-3s have anti-inflammatory properties that may help with asthma symptoms.  Vitamin D: Low levels of vitamin D have been linked to worse asthma control. Include fortified foods, fatty fish, or consider supplements if levels are low (consult your doctor first).  Avoid triggers: Some people find that certain foods (e.g., sulfites in processed foods, dairy, or gluten) can worsen their asthma. Keep a food diary to identify potential triggers.  Maintain a healthy weight: Excess weight can worsen asthma symptoms, so a balanced diet that helps you maintain a healthy weight is important.  Exercise  Improves lung function: Regular exercise can strengthen your respiratory muscles and improve lung capacity, which may help reduce asthma symptoms over time.  Cardiovascular health: Activities like swimming, walking, or cycling can improve overall fitness and reduce the risk of asthma exacerbations.  Breathing exercises: Techniques like pursed-lip breathing or yoga-based breathing (pranayama) can help improve breathing control and reduce stress, which may trigger asthma.  Warm-up and cool-down: Always warm up before exercise and cool down afterward to prevent exercise-induced asthma symptoms.  Choose the right environment: Exercise in warm, humid environments (like indoor pools) and avoid cold, dry air or areas with high pollen or pollution levels.  Important Considerations  Work with your doctor: Never stop or reduce your asthma medication without consulting your healthcare provider. They can help you create a personalized plan to manage your asthma effectively.  Monitor symptoms: Keep track of your symptoms and triggers to understand how diet and exercise affect your asthma.  Emergency plan: Always have a rescue inhaler on hand, especially during exercise, in case of an asthma attack.  While diet and exercise can improve your overall health and potentially reduce reliance on medication in mild cases, they are not a cure for asthma. Always follow your doctor's advice and treatment plan to ensure your asthma is well-controlled. | 4 | 4 | 4 | 4 | 4 | 4 | 4 |
| **Q10** | The doctor prescribed inhaled steroids after diagnosing asthma. Will I become dependent on them in the future? | No, you will not become dependent on inhaled steroids. Inhaled steroids are a common and effective treatment for asthma, and they work by reducing inflammation in the airways, which helps prevent asthma symptoms and attacks. They are not addictive, and using them as prescribed does not lead to physical dependence.  However, it's important to use them consistently and as directed by your doctor to manage your asthma effectively. Stopping them abruptly without medical advice could lead to worsening symptoms. If you have concerns about long-term use or side effects, discuss them with your doctor, who can provide guidance tailored to your specific situation. | 4 | 4 | 4 | 4 | 4 | 4 | 4 |
|  | **Assessment and Management** |  |  |  |  |  |  |  |  |
| **Q1** | Will asthma affect my life expectancy? How can I manage my condition to avoid serious consequences? | Asthma, when well-managed, typically does not significantly affect life expectancy. However, poorly controlled asthma can lead to serious complications, such as severe asthma attacks, respiratory failure, or even death in rare cases. The key to avoiding these consequences is effective management of your condition. Here are some strategies to help you manage asthma and maintain a good quality of life:  1. Follow Your Asthma Action Plan  Work with your healthcare provider to create a personalized asthma action plan. This plan should outline:  Daily management strategies.  How to recognize worsening symptoms.  Steps to take during an asthma attack, including when to seek emergency care.  2. Take Medications as Prescribed  Controller Medications: These are taken daily to reduce inflammation and prevent symptoms (e.g., inhaled corticosteroids).  Reliever Medications: These are used as needed to quickly relieve symptoms during an attack (e.g., short-acting beta-agonists like albuterol).  Never stop or adjust your medications without consulting your doctor.  3. Identify and Avoid Triggers  Common asthma triggers include:  Allergens (e.g., pollen, dust mites, pet dander).  Irritants (e.g., smoke, pollution, strong odors).  Respiratory infections.  Cold air or weather changes.  Exercise (use a reliever inhaler before exercise if recommended).  Take steps to minimize exposure to your specific triggers.  4. Monitor Your Symptoms  Use a peak flow meter to track your lung function regularly.  Keep a symptom diary to identify patterns or worsening signs.  5. Get Regular Check-Ups  Visit your healthcare provider regularly to review your asthma control and adjust your treatment plan if needed.  6. Stay Active and Healthy  Exercise regularly, as it can improve lung function and overall health. If exercise triggers your asthma, use your reliever inhaler beforehand and choose activities that are less likely to cause symptoms (e.g., swimming).  Maintain a healthy diet and weight, as obesity can worsen asthma symptoms.  Avoid smoking and secondhand smoke.  7. Be Prepared for Emergencies  Know the signs of a severe asthma attack (e.g., difficulty speaking, blue lips or fingernails, no improvement after using a reliever inhaler).  Seek immediate medical attention if your symptoms worsen rapidly or if you experience a life-threatening attack.  8. Stay Informed and Educated  Learn as much as you can about asthma and its management.  Stay up-to-date on new treatments or strategies.  9. Consider Allergy Testing and Immunotherapy  If allergies are a significant trigger, allergy testing and immunotherapy (e.g., allergy shots) may help reduce your sensitivity over time.  10. Manage Stress  Stress and anxiety can worsen asthma symptoms. Practice relaxation techniques, such as deep breathing, meditation, or yoga.  By taking these steps, you can effectively manage your asthma, reduce the risk of complications, and maintain a normal life expectancy. Always work closely with your healthcare provider to ensure your treatment plan is tailored to your needs. | 4 | 4 | 4 | 4 | 4 | 4 | 4 |
| **Q2** | I have asthma but still smoke. Will this worsen my condition? | Yes, smoking will almost certainly worsen your asthma. Here’s why:  Increased Inflammation: Smoking irritates the airways, causing inflammation and making them more sensitive. This can trigger asthma symptoms like wheezing, coughing, and shortness of breath.  Reduced Lung Function: Smoking damages the lungs over time, reducing their ability to function properly. For someone with asthma, this can make breathing even more difficult.  More Frequent and Severe Attacks: Smoking can increase the frequency and severity of asthma attacks, which can be dangerous and potentially life-threatening.  Reduced Response to Medication: Smoking can make asthma medications less effective, meaning you might need higher doses or additional treatments to manage your symptoms.  Higher Risk of Complications: Smoking with asthma increases the risk of developing other respiratory conditions, such as chronic obstructive pulmonary disease (COPD) or respiratory infections.  If you have asthma, quitting smoking is one of the most important steps you can take to protect your lung health and improve your quality of life. Consider reaching out to a healthcare professional for support and resources to help you quit. | 4 | 4 | 4 | 4 | 4 | 4 | 4 |
| **Q3** | What symptoms should I watch out for during an asthma attack, and when should I go to the hospital for treatment? | During an asthma attack, the airways become inflamed and narrowed, making it difficult to breathe. It's important to recognize the symptoms early and know when to seek emergency medical care. Here are the key symptoms to watch out for:  Symptoms of an Asthma Attack:  Shortness of breath: Difficulty breathing or feeling like you can't catch your breath.  Wheezing: A whistling or squeaky sound when you breathe, especially when exhaling.  Coughing: Persistent coughing, which may worsen at night or early in the morning.  Chest tightness: A feeling of pressure or pain in the chest.  Rapid breathing: Breathing faster than normal.  Difficulty speaking: Trouble completing sentences due to shortness of breath.  Retractions: The skin around the ribs or neck pulls in with each breath.  Blue lips or fingernails: A sign of low oxygen levels in the blood.  Anxiety or panic: Feeling extremely anxious or agitated due to breathing difficulties.  When to Go to the Hospital:  Seek emergency medical care immediately if you or someone else experiences any of the following:  Severe shortness of breath: Inability to speak, eat, or sleep due to breathing difficulties.  No improvement after using a rescue inhaler: Symptoms persist or worsen even after using a quick-relief inhaler (e.g., albuterol).  Peak flow meter readings in the red zone: If you use a peak flow meter and your reading is less than 50% of your personal best.  Blue lips or fingernails: Indicates a lack of oxygen.  Rapid worsening of symptoms: Symptoms escalate quickly, and you feel like you can't breathe.  Confusion or drowsiness: A sign of severe oxygen deprivation.  Inability to walk or talk: Struggling to move or speak due to breathlessness.  What to Do While Waiting for Help:  Stay calm and sit upright to help open the airways.  Use your rescue inhaler as directed (usually every 4 hours during an attack).  Avoid lying down, as this can make breathing more difficult.  If you have a written asthma action plan, follow the steps outlined.  Asthma attacks can be life-threatening, so it's crucial to act quickly and seek medical attention if symptoms are severe or not improving. Always err on the side of caution and consult a healthcare professional if you're unsure. | 4 | 4 | 4 | 4 | 4 | 4 | 4 |
| **Q4** | If I have asthma, how can I assess the severity of my condition? Are there self-assessment methods? | Assessing the severity of asthma is important for managing the condition effectively. While a healthcare professional should provide a formal diagnosis and treatment plan, there are self-assessment methods you can use to monitor your symptoms and gauge the severity of your asthma. Here are some approaches:  1. Asthma Symptom Diary  Keep a daily record of your symptoms, such as coughing, wheezing, shortness of breath, and chest tightness.  Note the frequency and intensity of symptoms, as well as any triggers (e.g., allergens, exercise, cold air).  Record how often you use your rescue inhaler (e.g., albuterol).  2. Peak Flow Monitoring  Use a peak flow meter to measure how well air moves out of your lungs. This device provides a numerical value (peak expiratory flow rate, or PEFR) that can help you track changes in your lung function.  Compare your daily readings to your personal best peak flow (determined when your asthma is well-controlled).  A significant drop in peak flow (e.g., below 80% of your personal best) may indicate worsening asthma.  3. Asthma Control Test (ACT)  The ACT is a validated questionnaire that helps assess how well your asthma is controlled over the past 4 weeks.  It includes questions about symptoms, rescue inhaler use, and the impact of asthma on daily activities.  Scores range from 5 to 25, with higher scores indicating better control. A score of 19 or below may suggest poorly controlled asthma.  4. Symptom Severity Assessment  Mild intermittent asthma: Symptoms occur less than twice a week, and nighttime symptoms occur less than twice a month.  Mild persistent asthma: Symptoms occur more than twice a week but not daily, and nighttime symptoms occur 3–4 times a month.  Moderate persistent asthma: Symptoms occur daily, and nighttime symptoms occur more than once a week.  Severe persistent asthma: Symptoms occur throughout the day, and nighttime symptoms occur frequently.  5. Monitor Rescue Inhaler Use  Frequent use of your rescue inhaler (e.g., more than twice a week) may indicate poor asthma control.  Needing your rescue inhaler more often than usual or feeling like it’s not working as well could signal worsening asthma.  6. Watch for Warning Signs  Severe shortness of breath, difficulty speaking, or bluish lips or fingernails require immediate medical attention.  Symptoms that don’t improve after using a rescue inhaler or that worsen quickly may indicate an asthma attack.  7. Consult Your Healthcare Provider  Share your self-assessment data with your doctor or asthma specialist.  They may perform additional tests, such as spirometry, to evaluate lung function and adjust your treatment plan.  When to Seek Emergency Care:  If you experience severe symptoms, such as extreme difficulty breathing, rapid worsening of symptoms, or if your peak flow drops below 50% of your personal best, seek emergency medical care immediately.  Self-assessment tools are helpful for monitoring your asthma, but they should not replace regular check-ups with your healthcare provider. Always follow your asthma action plan and consult your doctor if you notice any changes in your symptoms or control. | 4 | 4 | 4 | 4 | 4 | 4 | 4 |
| **Q5** | I’m an asthma patient, and I take medication daily. Once my condition stabilizes, can I stop taking it? | It's great that your asthma condition has stabilized, but it's important to approach any changes to your medication regimen with caution. Asthma is a chronic condition, and even if you feel well, the underlying inflammation and airway sensitivity may still be present. Stopping your medication without consulting your healthcare provider could lead to a worsening of your symptoms or an asthma attack.  Here are a few key points to consider:  Maintenance Medications: If you're on a daily maintenance medication (like inhaled corticosteroids), these are designed to control inflammation and prevent symptoms. Stopping them abruptly could lead to a flare-up.  Consult Your Doctor: Before making any changes to your medication, always discuss it with your healthcare provider. They may adjust your dosage or recommend a step-down approach rather than stopping completely.  Monitor Symptoms: Even if you feel stable, keep track of your symptoms and peak flow readings (if you use a peak flow meter). This can help you and your doctor assess whether your asthma is truly under control.  Rescue Inhaler: If you're using a rescue inhaler (like albuterol) frequently, it may indicate that your asthma is not fully controlled, and you should consult your doctor.  Lifestyle and Triggers: Continue to avoid known asthma triggers and maintain a healthy lifestyle to support your lung health.  In summary, don't stop your medication without medical advice. Work closely with your healthcare provider to ensure your asthma remains well-managed. | 4 | 4 | 4 | 4 | 4 | 4 | 4 |
| **Q6** | How can I use a peak flow meter to monitor my asthma symptoms? When is it most effective to use it? | Using a peak flow meter to monitor asthma symptoms is a practical way to track how well your lungs are functioning and to detect early signs of worsening asthma. Here's how to use it effectively:  Steps to Use a Peak Flow Meter:  Stand or Sit Upright: Ensure you are in a comfortable, upright position.  Reset the Meter: Slide the indicator to the bottom of the scale (zero position).  Take a Deep Breath: Inhale as deeply as possible.  Seal Your Lips Around the Mouthpiece: Place the meter in your mouth and close your lips tightly around it. Make sure your tongue does not block the mouthpiece.  Blow Out Hard and Fast: Exhale as forcefully and quickly as you can in one breath.  Record the Reading: Note the number where the indicator stops. This is your peak expiratory flow (PEF) rate.  Repeat Twice More: Perform the test two more times and record the highest of the three readings.  When to Use a Peak Flow Meter:  Daily Monitoring: Use it at the same time each day, typically in the morning and evening, to establish your personal best peak flow.  When Symptoms Worsen: Use it when you notice symptoms like coughing, wheezing, or shortness of breath to check if your lung function is declining.  During an Asthma Action Plan: Follow your asthma action plan, which may include using the meter to determine if you need to adjust your medication.  After Taking Medication: Use it to assess how well your medication is working.  Interpreting Results:  Green Zone (80-100% of your personal best): Your asthma is well-controlled.  Yellow Zone (50-80% of your personal best): Your asthma is worsening, and you may need to adjust your medication as per your action plan.  Red Zone (Below 50% of your personal best): Indicates a medical emergency. Seek immediate medical attention.  Tips for Effective Use:  Establish your personal best peak flow by recording your highest reading over 2-3 weeks when your asthma is well-controlled.  Keep a log of your readings to share with your healthcare provider.  Always follow your asthma action plan and consult your doctor if your readings consistently fall in the yellow or red zones.  Using a peak flow meter regularly can help you manage your asthma more effectively and take timely action to prevent severe attacks. | 4 | 4 | 4 | 4 | 4 | 4 | 4 |
| **Q7** | What factors affect the long-term outlook of asthma, and how can I avoid them? | The long-term outlook of asthma can be influenced by several factors, including:  1. Severity of Asthma  Mild vs. Severe Asthma: Individuals with severe asthma may experience more frequent symptoms and exacerbations, which can impact long-term lung function and quality of life.  Early Diagnosis and Treatment: Early and effective management can improve long-term outcomes.  2. Adherence to Treatment  Medication Compliance: Consistently using prescribed medications, such as inhaled corticosteroids and bronchodilators, can prevent exacerbations and maintain lung function.  Regular Follow-ups: Regular check-ups with a healthcare provider ensure that the treatment plan is effective and adjusted as needed.  3. Environmental Triggers  Allergens: Exposure to allergens like pollen, dust mites, pet dander, and mold can worsen asthma symptoms.  Irritants: Tobacco smoke, air pollution, strong odors, and chemical fumes can trigger asthma attacks.  Avoidance Strategies: Identifying and minimizing exposure to these triggers can help control asthma symptoms.  4. Lifestyle Factors  Smoking: Smoking or exposure to secondhand smoke can exacerbate asthma and lead to a decline in lung function.  Obesity: Excess weight can worsen asthma symptoms and reduce the effectiveness of medications.  Physical Activity: Regular exercise can improve lung function and overall health, but it’s important to manage exercise-induced asthma with proper medication.  5. Comorbid Conditions  Allergic Rhinitis, Sinusitis, and GERD: These conditions can exacerbate asthma symptoms and should be managed appropriately.  Mental Health: Stress, anxiety, and depression can worsen asthma control. Managing mental health is crucial for overall well-being.  6. Genetic Factors  Family History: A family history of asthma or other allergic conditions can influence the severity and persistence of asthma.  7. Infections  Respiratory Infections: Viral infections, such as the common cold or flu, can trigger asthma exacerbations. Vaccinations (e.g., flu and pneumonia vaccines) can help reduce the risk.  How to Avoid Negative Factors:  Medication Adherence: Take prescribed medications as directed and attend regular medical check-ups.  Trigger Avoidance: Identify and minimize exposure to environmental triggers.  Healthy Lifestyle: Maintain a healthy weight, avoid smoking, and engage in regular physical activity.  Manage Comorbidities: Treat conditions like allergies, GERD, and mental health issues.  Vaccinations: Stay up-to-date with vaccinations to prevent respiratory infections.  Education and Action Plan: Work with your healthcare provider to develop an asthma action plan and educate yourself about asthma management.  By addressing these factors, you can improve your long-term asthma outlook and maintain a better quality of life. | 4 | 4 | 4 | 4 | 4 | 4 | 4 |
| **Q8** | I’ve had asthma for two years. I no longer have wheezing, but I still feel short of breath after physical activity. What should I do? | It's great that your wheezing has improved, but feeling short of breath after physical activity suggests that your asthma may not be fully controlled. Here are some steps you can take:  Consult Your Doctor: Schedule an appointment with your healthcare provider to review your symptoms and asthma management plan. They may need to adjust your medication or investigate other potential causes of your shortness of breath.  Review Your Medications:  Ensure you’re using your controller medication (e.g., inhaled corticosteroids) regularly, as prescribed, even if you feel well.  Use your rescue inhaler (e.g., albuterol) before physical activity if recommended by your doctor.  Monitor Your Symptoms: Keep a diary of when you feel short of breath, the intensity of the activity, and any triggers. This can help your doctor tailor your treatment.  Consider Exercise-Induced Asthma: Some people with asthma experience symptoms primarily during or after exercise. Your doctor may recommend specific strategies, such as a warm-up routine or pre-exercise medication.  Improve Your Fitness Gradually: Regular physical activity can improve lung function and reduce asthma symptoms over time. Start with low-intensity exercises and gradually increase intensity as tolerated.  Avoid Triggers: Identify and avoid any environmental triggers (e.g., pollen, cold air, or pollution) that might worsen your symptoms during physical activity.  Breathing Techniques: Practice breathing exercises, such as pursed-lip breathing or diaphragmatic breathing, to help manage shortness of breath.  Check for Other Conditions: If your symptoms persist, your doctor may investigate other possible causes, such as anxiety, anemia, or heart-related issues.  Asthma management is highly individual, so working closely with your healthcare provider is key to finding the right approach for you. | 4 | 4 | 4 | 4 | 4 | 4 | 4 |
| **Q9** | Can asthma be completely cured? Will I be able to live a normal life afterward? | Asthma is a chronic condition that currently has no complete cure, but it can be effectively managed with proper treatment and lifestyle adjustments. Many people with asthma are able to live normal, active lives by following their treatment plan, which often includes:  Medications: Such as inhalers (e.g., bronchodilators and corticosteroids) to control symptoms and prevent flare-ups.  Avoiding Triggers: Identifying and minimizing exposure to allergens, irritants, or other factors that worsen symptoms (e.g., pollen, smoke, or cold air).  Regular Monitoring: Keeping track of symptoms and lung function with tools like peak flow meters.  Healthy Lifestyle: Maintaining a balanced diet, exercising regularly (with precautions), and managing stress.  While asthma may not go away completely, many individuals achieve long-term symptom control and can participate in all normal activities, including sports and exercise. It’s important to work closely with your healthcare provider to develop a personalized asthma action plan. If your symptoms are well-managed, you can expect to live a full and normal life. | 4 | 4 | 4 | 4 | 4 | 4 | 4 |
| **Q10** | I have asthma, and my condition is well-controlled, but why haven’t I seen an overall improvement in my health? | Asthma is a chronic condition that primarily affects the airways, causing inflammation and narrowing, which can lead to symptoms like wheezing, shortness of breath, and coughing. While having your asthma well-controlled is a significant achievement, it doesn't necessarily guarantee an overall improvement in your health for several reasons:  1. Asthma is Just One Aspect of Your Health  Even if your asthma is well-managed, other factors such as diet, exercise, sleep, stress levels, and other underlying health conditions can impact your overall well-being. If these areas are not optimized, you may not feel a significant improvement in your health.  2. Lifestyle Factors  Physical Activity: If your asthma has limited your ability to exercise in the past, you might still be dealing with the effects of a sedentary lifestyle, such as reduced cardiovascular fitness or muscle strength.  Diet: Poor nutrition can affect your energy levels, immune function, and overall health, even if your asthma is under control.  Sleep: Asthma symptoms, especially at night, can disrupt sleep quality, leading to fatigue and other health issues.  3. Comorbid Conditions  Asthma often coexists with other conditions like allergies, sinusitis, gastroesophageal reflux disease (GERD), or anxiety/depression. These conditions can affect your overall health and may require separate management.  4. Medication Side Effects  Some asthma medications, particularly long-term use of oral corticosteroids, can have side effects like weight gain, bone density loss, or increased blood pressure, which might impact your overall health.  5. Chronic Inflammation  Asthma involves chronic inflammation in the airways, and this inflammatory state can sometimes have systemic effects on the body, potentially contributing to fatigue or other symptoms.  6. Psychological Factors  Living with a chronic condition like asthma can lead to stress, anxiety, or depression, which can negatively impact your overall health and quality of life.  7. Environmental Triggers  Even if your asthma is controlled, ongoing exposure to environmental triggers like air pollution, allergens, or smoke can still affect your respiratory health and overall well-being.  What You Can Do:  Holistic Health Approach: Focus on improving other aspects of your health, such as maintaining a balanced diet, engaging in regular physical activity (as tolerated), and managing stress.  Regular Check-ups: Ensure you’re addressing any other health conditions or risk factors with your healthcare provider.  Monitor Symptoms: Keep track of any new or worsening symptoms and discuss them with your doctor to rule out other underlying issues.  Mental Health: Consider seeking support for stress, anxiety, or depression if these are affecting your quality of life.  If you feel your overall health isn’t improving despite well-controlled asthma, it’s a good idea to discuss your concerns with your healthcare provider. They can help identify any other factors that might be contributing and develop a comprehensive plan to improve your overall well-being. | 4 | 4 | 4 | 4 | 4 | 4 | 4 |
| **Q11** | The doctor diagnosed me with asthma. If my symptoms don’t improve after using medication, how can I tell if I need to seek further medical attention? | If your asthma symptoms don’t improve after using your prescribed medication, it’s important to monitor your condition closely and seek further medical attention if you notice any of the following:  Persistent or Worsening Symptoms: If symptoms like wheezing, shortness of breath, chest tightness, or coughing continue or get worse despite using your inhaler or other medications.  Frequent Use of Rescue Inhaler: If you find yourself using your rescue inhaler (e.g., albuterol) more often than recommended (typically more than 2–3 times per week).  Difficulty Performing Daily Activities: If your symptoms interfere with your ability to carry out normal activities, such as walking, talking, or sleeping.  Peak Flow Readings: If you use a peak flow meter and notice a significant drop in your peak expiratory flow (PEF) readings, especially if they fall into the "red zone" (typically below 50% of your personal best).  Severe Symptoms: If you experience severe shortness of breath, rapid breathing, or difficulty speaking in full sentences.  Lips or Fingernails Turning Blue: This is a sign of low oxygen levels and requires immediate medical attention.  No Improvement After Using Medication: If you don’t feel any relief after using your rescue inhaler or other prescribed medications.  Signs of an Asthma Attack: Symptoms such as extreme difficulty breathing, chest retractions (skin pulling in around the ribs or neck), or feeling panicked due to breathing difficulties.  If you experience any of these signs, seek medical attention promptly. In severe cases, go to the nearest emergency room or call for emergency help. Uncontrolled asthma can be life-threatening, so it’s crucial to act quickly if your symptoms aren’t improving. Always follow up with your doctor to adjust your asthma action plan if needed. | 4 | 4 | 4 | 4 | 4 | 4 | 4 |
|  | **Differential Diagnosis** |  |  |  |  |  |  |  |  |
| **Q1** | Besides asthma, what other conditions could cause coughing and wheezing? | Coughing and wheezing can be symptoms of various conditions besides asthma. Here are some other potential causes:  Chronic Obstructive Pulmonary Disease (COPD): This includes chronic bronchitis and emphysema, which can cause airway inflammation and narrowing, leading to coughing and wheezing.  Bronchitis: Both acute and chronic bronchitis involve inflammation of the bronchial tubes, which can produce coughing and wheezing.  Respiratory Infections: Viral or bacterial infections, such as the common cold, flu, pneumonia, or bronchiolitis, can cause inflammation and mucus production in the airways, leading to these symptoms.  Allergies: Allergic reactions to pollen, dust, pet dander, or other allergens can cause airway inflammation and wheezing.  Gastroesophageal Reflux Disease (GERD): Acid reflux can irritate the throat and airways, leading to coughing and sometimes wheezing.  Postnasal Drip: Mucus from the nasal passages dripping down the throat can irritate the airways and cause coughing and wheezing.  Heart Failure: Fluid buildup in the lungs due to heart failure can cause coughing and wheezing, often accompanied by shortness of breath.  Foreign Body Aspiration: Inhaling a foreign object can block the airways and cause wheezing and coughing.  Vocal Cord Dysfunction (VCD): This condition involves abnormal movement of the vocal cords, which can mimic asthma symptoms, including wheezing.  Cystic Fibrosis: A genetic disorder that causes thick, sticky mucus to build up in the lungs, leading to chronic coughing and wheezing.  Anaphylaxis: A severe allergic reaction can cause airway constriction, leading to wheezing and coughing.  Pulmonary Edema: Fluid accumulation in the lungs, often due to heart problems, can cause wheezing and coughing.  Interstitial Lung Disease: A group of disorders causing inflammation and scarring of lung tissue, which can lead to coughing and wheezing.  Smoking or Exposure to Irritants: Inhaling smoke, chemicals, or other irritants can cause airway inflammation and symptoms like coughing and wheezing.  If you or someone else is experiencing persistent coughing and wheezing, it's important to seek medical evaluation to determine the underlying cause and appropriate treatment. | 3 | 3 | 4 | 4 | 4 | 4 | 4 |
| **Q2** | I have chronic coughing and shortness of breath. The doctor suspects asthma. Could it be something else? | While asthma is a common cause of chronic coughing and shortness of breath, there are several other conditions that could mimic these symptoms. It’s important to consider other possibilities, especially if your symptoms don’t fully align with typical asthma or if they don’t respond well to standard asthma treatments. Here are some alternative conditions to consider:  1. Chronic Obstructive Pulmonary Disease (COPD)  COPD, which includes chronic bronchitis and emphysema, often presents with chronic cough, shortness of breath, and wheezing. It’s more common in individuals with a history of smoking or long-term exposure to lung irritants.  2. Gastroesophageal Reflux Disease (GERD)  Acid reflux can cause stomach acid to irritate the throat and airways, leading to chronic coughing and sometimes shortness of breath. Symptoms may worsen at night or after eating.  3. Postnasal Drip  Chronic sinusitis or allergies can cause mucus to drip down the back of the throat, triggering a persistent cough and sometimes a sensation of breathlessness.  4. Vocal Cord Dysfunction (VCD)  VCD can mimic asthma, causing episodes of shortness of breath and wheezing. It occurs when the vocal cords close improperly during breathing.  5. Heart-Related Issues  Conditions like heart failure or coronary artery disease can cause shortness of breath and sometimes a cough, especially if fluid builds up in the lungs (a condition called pulmonary edema).  6. Infections  Chronic respiratory infections, such as tuberculosis or fungal infections, can cause persistent coughing and breathing difficulties.  7. Interstitial Lung Disease (ILD)  ILD refers to a group of disorders that cause scarring of lung tissue, leading to chronic cough, shortness of breath, and reduced lung function.  8. Allergies  Environmental allergies can cause chronic coughing and shortness of breath, especially if they trigger inflammation in the airways.  9. Medication Side Effects  Certain medications, such as ACE inhibitors (used for high blood pressure), can cause a chronic cough as a side effect.  10. Anxiety or Hyperventilation Syndrome  Anxiety can lead to shortness of breath and a sensation of not being able to take a deep breath, which may be mistaken for asthma.  11. Occupational or Environmental Exposures  Exposure to dust, chemicals, or other irritants in the workplace or environment can cause chronic respiratory symptoms.  12. Less Common Conditions  Rare conditions like sarcoidosis, bronchiectasis, or pulmonary fibrosis could also cause similar symptoms.  Next Steps:  To determine the exact cause, your doctor may recommend:  Pulmonary function tests (to assess lung function).  Imaging studies (like a chest X-ray or CT scan).  Blood tests (to check for infections or inflammation).  Allergy testing (if allergies are suspected).  pH monitoring or endoscopy (if GERD is suspected).  Cardiac evaluation (if heart-related issues are a concern).  It’s important to provide your doctor with a detailed history of your symptoms, including when they started, what triggers or relieves them, and any other associated symptoms (e.g., fever, weight loss, heartburn). This will help narrow down the diagnosis and ensure you receive the most appropriate treatment. | 4 | 4 | 4 | 4 | 4 | 4 | 4 |
| **Q3** | How can I distinguish asthma from other conditions, like chronic bronchitis or allergic cough | Distinguishing asthma from other conditions like chronic bronchitis or allergic cough can be challenging because they often share similar symptoms, such as coughing, wheezing, and shortness of breath. However, there are key differences in clinical features, triggers, and diagnostic tests that can help differentiate them:  1. Asthma  Symptoms: Episodic wheezing, shortness of breath, chest tightness, and coughing, often worse at night or early morning.  Triggers: Allergens (e.g., pollen, dust mites), exercise, cold air, viral infections, or irritants (e.g., smoke).  Pattern: Symptoms are variable and often reversible, either spontaneously or with bronchodilators.  Diagnostic Tests:  Spirometry with bronchodilator reversibility: Shows improvement in airflow after using a bronchodilator (e.g., FEV1 increases by ≥12% and 200 mL).  Peak expiratory flow (PEF) variability: Daily variability in PEF measurements.  Methacholine challenge test: Positive in asthma (airway hyperresponsiveness).  FeNO (fractional exhaled nitric oxide): Elevated in eosinophilic airway inflammation.  Response to Treatment: Symptoms improve with inhaled corticosteroids (ICS) and bronchodilators.  2. Chronic Bronchitis  Symptoms: Chronic productive cough (with sputum) for at least 3 months in 2 consecutive years, often with shortness of breath.  Triggers: Smoking or long-term exposure to irritants (e.g., air pollution, dust).  Pattern: Symptoms are persistent and not fully reversible, even with bronchodilators.  Diagnostic Tests:  Spirometry: May show obstructive pattern, but limited reversibility with bronchodilators.  Chest X-ray: May show hyperinflation or signs of chronic lung disease.  Sputum analysis: May reveal chronic inflammation or infection.  Response to Treatment: Limited improvement with bronchodilators; focus is on smoking cessation, pulmonary rehabilitation, and managing exacerbations.  3. Allergic Cough (or Cough-Variant Asthma)  Symptoms: Chronic dry cough (without wheezing or sputum) that persists for weeks or months.  Triggers: Allergens (e.g., pollen, dust, pet dander), cold air, or irritants.  Pattern: Cough is the primary or only symptom, often worse at night or after exposure to triggers.  Diagnostic Tests:  Spirometry: May be normal or show mild obstruction.  Bronchoprovocation test: Positive in cough-variant asthma.  FeNO: May be elevated if allergic inflammation is present.  Response to Treatment: Improves with inhaled corticosteroids or antihistamines if allergy-related.  Key Differences   \| Feature \| Asthma \| Chronic Bronchitis \| Allergic Cough \| \| --- \| --- \| --- \| --- \| \| Cough \| Dry or productive \| Productive (with sputum) \| Dry \| \| Wheezing \| Common \| Less common \| Rare \| \| Symptom Pattern \| Episodic, reversible \| Persistent, progressive \| Persistent, triggered \| \| Triggers \| Allergens, exercise, cold \| Smoking, irritants \| Allergens, irritants \| \| Spirometry \| Reversible obstruction \| Fixed obstruction \| Often normal \| \| FeNO \| Elevated \| Normal or low \| May be elevated \|   When to See a Doctor  If you’re unsure about your symptoms, consult a healthcare provider. They may perform a detailed history, physical exam, and diagnostic tests to confirm the diagnosis and recommend appropriate treatment.  Let me know if you need further clarification! | 4 | 4 | 4 | 4 | 4 | 4 | 4 |
| **Q4** | What’s the difference between allergic asthma and bronchial asthma? | Allergic asthma and bronchial asthma are closely related, but they differ in their triggers and underlying mechanisms:  1. Definition and Triggers:  Allergic Asthma: This is a subtype of asthma triggered by exposure to allergens such as pollen, dust mites, pet dander, mold, or certain foods. It is an immune-mediated response where the body's immune system overreacts to these allergens, leading to asthma symptoms.  Bronchial Asthma: This is a broader term referring to chronic inflammation of the airways, causing symptoms like wheezing, shortness of breath, chest tightness, and coughing. It can be triggered by both allergic and non-allergic factors, such as cold air, exercise, stress, smoke, or respiratory infections.  2. Underlying Mechanism:  Allergic Asthma: Involves an IgE-mediated immune response. When allergens are inhaled, the immune system produces IgE antibodies, which trigger the release of inflammatory chemicals like histamine, leading to airway inflammation and constriction.  Bronchial Asthma: While it can involve allergic mechanisms, it also includes non-allergic pathways. The inflammation in the airways is caused by various factors, not limited to allergens, and can involve other immune cells and mediators.  3. Diagnosis:  Allergic Asthma: Often diagnosed through allergy testing (e.g., skin prick tests or blood tests for specific IgE antibodies) to identify the allergens responsible for triggering symptoms.  Bronchial Asthma: Diagnosed based on clinical symptoms, lung function tests (e.g., spirometry), and response to asthma medications, regardless of the specific triggers.  4. Treatment:  Allergic Asthma: Treatment includes avoiding allergens, using inhaled corticosteroids, and possibly allergen immunotherapy (allergy shots) to desensitize the immune system to specific allergens.  Bronchial Asthma: Treatment focuses on controlling inflammation and symptoms with inhaled corticosteroids, bronchodilators, and other asthma medications. If allergies are a contributing factor, similar treatments for allergic asthma may also be used.  5. Overlap:  Allergic asthma is a subset of bronchial asthma. Many people with bronchial asthma have allergic triggers, but not all cases of bronchial asthma are caused by allergies.  In summary, allergic asthma is a specific type of bronchial asthma triggered by allergens, while bronchial asthma encompasses a wider range of triggers and mechanisms. Both conditions involve chronic airway inflammation and share similar symptoms and treatments, but the management of allergic asthma may include additional focus on allergen avoidance and immunotherapy. | 4 | 4 | 4 | 4 | 3 | 4 | 4 |
| **Q5** | If I often feel short of breath, does that mean I have asthma, or could it be something else? | Feeling short of breath can be caused by a variety of conditions, and while asthma is one possibility, it is not the only one. Here are some potential causes:  Asthma: This chronic condition involves inflammation and narrowing of the airways, leading to episodes of wheezing, shortness of breath, chest tightness, and coughing, often triggered by allergens, exercise, or cold air.  Anxiety or Panic Attacks: Anxiety can cause hyperventilation (rapid breathing), leading to shortness of breath, chest tightness, and a feeling of being unable to get enough air.  Chronic Obstructive Pulmonary Disease (COPD): This includes conditions like chronic bronchitis and emphysema, often caused by smoking, which lead to difficulty breathing, especially during physical activity.  Heart Problems: Conditions such as heart failure, arrhythmias, or coronary artery disease can cause shortness of breath, often accompanied by fatigue, swelling in the legs, or chest pain.  Infections: Respiratory infections like pneumonia, bronchitis, or COVID-19 can cause shortness of breath, along with fever, cough, and fatigue.  Anemia: A low red blood cell count can reduce oxygen delivery to tissues, causing shortness of breath, especially during exertion.  Obesity: Excess weight can put pressure on the lungs and diaphragm, making it harder to breathe.  Allergies or Environmental Irritants: Exposure to allergens, pollutants, or chemicals can cause shortness of breath in sensitive individuals.  Pulmonary Embolism: A blood clot in the lungs can cause sudden shortness of breath, chest pain, and coughing, and is a medical emergency.  Other Lung Conditions: Conditions like pulmonary fibrosis, interstitial lung disease, or a collapsed lung (pneumothorax) can also cause breathing difficulties.If you frequently experience shortness of breath, it’s important to see a healthcare provider for a proper evaluation. They may perform tests such as lung function tests, blood work, imaging (like a chest X-ray), or an electrocardiogram (ECG) to determine the underlying cause. Early diagnosis and treatment are key to managing any potential condition effectively. | 4 | 4 | 4 | 4 | 4 | 4 | 4 |
| **Q6** | I only have a cough without wheezing. The doctor says I have asthma. Is this diagnosis accurate? | Asthma can present with a variety of symptoms, and while wheezing is a common feature, it is not the only one. Some people with asthma may primarily experience coughing, especially at night or early in the morning, or in response to specific triggers like cold air, exercise, or allergens. This is sometimes referred to as cough-variant asthma.  If your doctor has diagnosed you with asthma based on your symptoms, medical history, and possibly additional tests (such as spirometry or peak flow measurements), the diagnosis could be accurate even in the absence of wheezing. Cough-variant asthma is a recognized subtype of asthma, and the cough is often dry and persistent.  However, if you have doubts about the diagnosis, it’s always reasonable to seek a second opinion or ask your doctor to explain the reasoning behind the diagnosis in more detail. Other conditions, such as postnasal drip, gastroesophageal reflux disease (GERD), or chronic bronchitis, can also cause a persistent cough, so it’s important to ensure that these have been ruled out.  If you’ve been prescribed asthma medication (like an inhaler), using it as directed and monitoring whether your symptoms improve can also help confirm the diagnosis. If the treatment works, it may support the idea that your cough is related to asthma. | 4 | 3 | 4 | 4 | 4 | 4 | 4 |
|  | **COVID-19 Related** |  |  |  |  |  |  |  |  |
| **Q1** | As an asthma patient, am I at higher risk of contracting COVID-19? How should I protect myself? | As an asthma patient, you may not necessarily be at a higher risk of contracting COVID-19 compared to the general population, but if you do contract the virus, you could be at a higher risk of experiencing more severe symptoms. This is because COVID-19 primarily affects the respiratory system, and asthma is a chronic respiratory condition that can make it harder for your lungs to handle the virus.  Here are some steps you can take to protect yourself:  Follow General COVID-19 Prevention Guidelines:  Wash Your Hands Frequently: Use soap and water for at least 20 seconds, especially after being in public places, or use hand sanitizer with at least 60% alcohol.  Wear a Mask: In situations where social distancing is difficult, wearing a mask can help reduce the spread of the virus.  Practice Social Distancing: Keep at least 6 feet away from others, especially in crowded places.  Avoid Touching Your Face: This can help prevent the virus from entering your body through your eyes, nose, or mouth.  Manage Your Asthma:  Take Your Medications as Prescribed: Ensure that you are using your inhalers and other asthma medications as directed by your healthcare provider. Well-controlled asthma is less likely to exacerbate if you contract COVID-19.  Have an Asthma Action Plan: Work with your healthcare provider to create or update your asthma action plan, which should include instructions on what to do if your symptoms worsen.  Monitor Your Symptoms: Keep track of your asthma symptoms and peak flow readings if you use a peak flow meter.  Stay Informed and Prepared:  Keep a Supply of Medications: Ensure you have a sufficient supply of your asthma medications and other essentials in case you need to stay home for an extended period.  Stay Updated on Local Guidelines: Follow local health department guidelines and stay informed about the COVID-19 situation in your area.  Seek Medical Advice When Needed:  Contact Your Healthcare Provider: If you experience worsening asthma symptoms or suspect you have been exposed to COVID-19, contact your healthcare provider for advice. They may recommend specific actions or adjustments to your treatment plan.  Emergency Situations: Seek emergency medical care if you experience severe difficulty breathing, persistent chest pain, confusion, or bluish lips or face.  By taking these precautions and managing your asthma effectively, you can reduce your risk of severe illness from COVID-19. Always consult with your healthcare provider for personalized advice and recommendations. | 4 | 4 | 4 | 4 | 4 | 4 | 4 |
| **Q2** | If I contract COVID-19, how should I manage it as someone with asthma? | If you have asthma and contract COVID-19, it's important to take extra precautions to manage your symptoms and reduce the risk of complications. Here are some steps to follow:  1. Monitor Your Symptoms Closely  Keep track of your asthma symptoms (e.g., wheezing, shortness of breath, chest tightness) and COVID-19 symptoms (e.g., fever, cough, fatigue, loss of taste or smell).  Use a peak flow meter to monitor your lung function regularly, as COVID-19 can affect your respiratory system.  2. Continue Your Asthma Medications  Do not stop your asthma controller medications (e.g., inhaled corticosteroids) unless instructed by your healthcare provider. These medications help keep your asthma under control and reduce the risk of exacerbations.  Use your rescue inhaler (e.g., albuterol) as needed for sudden symptoms.  If you use a nebulizer, take precautions to avoid spreading the virus (e.g., use it in a well-ventilated area, clean it thoroughly after each use).  3. Follow COVID-19 Treatment Guidelines  Rest, stay hydrated, and manage symptoms like fever with over-the-counter medications (e.g., acetaminophen or ibuprofen, as recommended by your doctor).  If you are at high risk for severe COVID-19, contact your healthcare provider promptly. They may recommend antiviral treatments like Paxlovid or other therapies.  4. Seek Medical Attention if Necessary  Contact your healthcare provider if your asthma symptoms worsen or if you experience difficulty breathing, chest pain, or a persistent high fever.  Seek emergency care if you have severe shortness of breath, confusion, bluish lips or face, or other signs of a severe asthma attack or COVID-19 complications.  5. Prevent the Spread of COVID-19  Isolate yourself from others to avoid spreading the virus.  Wear a mask if you need to be around others, and practice good hand hygiene.  6. Stay in Touch with Your Healthcare Provider  Inform your doctor that you have COVID-19 and asthma. They can provide personalized advice and monitor your condition.  Follow up regularly, especially if your symptoms change or worsen.  7. Take Steps to Reduce Asthma Triggers  Avoid known asthma triggers (e.g., smoke, dust, strong odors) that could worsen your symptoms while you recover.  Keep your indoor air clean by using an air purifier and avoiding allergens.  8. Stay Calm and Manage Stress  Stress and anxiety can worsen asthma symptoms. Practice relaxation techniques, such as deep breathing or meditation, to stay calm.  By carefully managing both your asthma and COVID-19, you can reduce the risk of complications and support your recovery. Always follow the guidance of your healthcare provider. | 4 | 4 | 4 | 4 | 4 | 4 | 4 |
| **Q3** | Should I get the COVID-19 vaccine? Will it affect my asthma? | Yes, you should get the COVID-19 vaccine, especially if you have asthma. Here’s why:  Increased Risk for Severe COVID-19: People with moderate to severe asthma may be at higher risk of developing severe illness if they contract COVID-19. The vaccine significantly reduces this risk.  Vaccine Safety: The COVID-19 vaccines authorized for use (e.g., Pfizer-BioNTech, Moderna, Novavax) have been shown to be safe and effective for people with asthma. There is no evidence that the vaccines worsen asthma symptoms.  No Live Virus: The mRNA vaccines (Pfizer and Moderna) and protein-based vaccines (Novavax) do not contain live virus, so they cannot cause COVID-19. This makes them safe for people with asthma or other chronic lung conditions.  Potential Side Effects: Some people may experience mild side effects after vaccination, such as fever, fatigue, or muscle aches, which are normal signs that your body is building protection. These side effects are temporary and do not indicate an asthma exacerbation.  Consult Your Doctor: If you have concerns about how the vaccine might interact with your asthma or medications, talk to your healthcare provider. They can provide personalized advice based on your medical history.  In summary, getting the COVID-19 vaccine is strongly recommended for individuals with asthma to protect against severe illness and complications from the virus. | 4 | 4 | 4 | 4 | 4 | 4 | 4 |
| **Q4** | Are COVID-19 symptoms more severe for asthma patients compared to others? What should I be especially mindful of? | COVID-19 can potentially cause more severe symptoms in individuals with asthma, as respiratory viruses like SARS-CoV-2 (the virus that causes COVID-19) may exacerbate asthma symptoms. Asthma is a chronic condition that affects the airways, and when combined with a respiratory infection, it can lead to increased inflammation and breathing difficulties. However, the severity of COVID-19 in asthma patients can vary depending on factors such as age, overall health, and how well their asthma is managed.  Here’s what asthma patients should be especially mindful of:  Asthma Control: Ensure your asthma is well-controlled by following your prescribed treatment plan, including the regular use of maintenance medications (e.g., inhaled corticosteroids). Poorly controlled asthma may increase the risk of complications.  Rescue Inhaler: Keep your rescue inhaler (e.g., albuterol) readily available in case of an asthma flare-up.  COVID-19 Prevention: Take extra precautions to avoid exposure to the virus. This includes practicing good hand hygiene, wearing masks in crowded or high-risk settings, and maintaining physical distance when possible.  Vaccination: Stay up to date with COVID-19 vaccinations and boosters, as they significantly reduce the risk of severe illness, hospitalization, and death. Consult your healthcare provider if you have concerns about the vaccine.  Monitor Symptoms: Be vigilant about any changes in your asthma symptoms or new symptoms such as fever, cough, shortness of breath, or loss of taste/smell. Seek medical advice promptly if you suspect COVID-19 or experience worsening asthma symptoms.  Action Plan: Have an asthma action plan in place, and discuss with your healthcare provider how to adjust your medications or treatment if you contract COVID-19.  Avoid Triggers: Minimize exposure to known asthma triggers (e.g., allergens, smoke, or pollution) to reduce the risk of exacerbations.  Stay Informed: Keep up to date with guidance from reputable health organizations like the CDC or WHO, as recommendations may evolve.  If you have asthma and contract COVID-19, contact your healthcare provider for personalized advice. They may recommend specific treatments or adjustments to your asthma management plan to help you recover safely. | 4 | 4 | 4 | 4 | 4 | 4 | 4 |
|  | **Mental Health** |  |  |  |  |  |  |  |  |
| **Q1** | I have asthma and often feel very tired and weak. Is this normal? | I'm not a doctor, but I can provide some general information that might help you understand your situation better. Asthma is a chronic condition that affects the airways, making it harder to breathe, especially during flare-ups. While asthma primarily affects the respiratory system, it can also lead to feelings of tiredness and weakness for several reasons:  Difficulty Breathing: When asthma symptoms worsen, your body may not get enough oxygen, which can leave you feeling fatigued and weak.  Poor Sleep: Asthma symptoms, such as coughing or shortness of breath, can disrupt your sleep, leading to daytime tiredness.  Medication Side Effects: Some asthma medications, particularly oral corticosteroids, can cause fatigue or weakness as a side effect.  Stress and Anxiety: Managing a chronic condition like asthma can be mentally and emotionally draining, contributing to feelings of exhaustion.  Reduced Physical Activity: If asthma limits your ability to exercise or stay active, it can lead to deconditioning, making you feel weaker over time.  When to Seek Medical Advice  While some fatigue can be expected with asthma, it’s important to consult your healthcare provider if:  Your fatigue is severe or persistent.  Your asthma symptoms are worsening or not well-controlled.  You experience additional symptoms like chest pain, dizziness, or unexplained weight loss.  Your doctor may want to adjust your asthma management plan, check for other underlying conditions (e.g., anemia, sleep apnea, or vitamin deficiencies), or explore whether your medications need to be modified.  In the meantime, make sure you’re following your asthma action plan, avoiding triggers, and taking your medications as prescribed. If you haven’t already, consider discussing your fatigue with your healthcare provider to ensure your asthma is well-managed and to rule out other potential causes. | 4 | 4 | 4 | 4 | 4 | 4 | 4 |
| **Q2** | Asthma is affecting my mood, and I often feel anxious. What can I do to manage this? | I'm sorry to hear that asthma is affecting your mood and causing anxiety. Managing both your physical and emotional health is important, and there are several strategies you can try to help cope with these feelings:  1. Work Closely with Your Healthcare Provider  Ensure your asthma is well-controlled by following your treatment plan, including taking prescribed medications (e.g., inhalers) and attending regular check-ups.  Discuss your anxiety with your doctor, as they may recommend additional support or adjustments to your treatment.  2. Practice Relaxation Techniques  Deep Breathing: Practice slow, controlled breathing exercises to help calm your mind and improve lung function. Techniques like diaphragmatic breathing or pursed-lip breathing can be helpful.  Meditation or Mindfulness: These practices can reduce anxiety and help you stay present. Apps like Calm or Headspace can guide you.  Progressive Muscle Relaxation: This involves tensing and relaxing different muscle groups to release tension.  3. Stay Active  Regular physical activity can improve both asthma control and mood. Choose asthma-friendly exercises like walking, swimming, or yoga, and always warm up properly.  Exercise releases endorphins, which can help reduce anxiety.  4. Identify and Avoid Triggers  Knowing and avoiding your asthma triggers (e.g., allergens, pollution, stress) can reduce flare-ups and the anxiety that comes with them.  Keep your environment clean and use an air purifier if needed.  5. Build a Support System  Talk to friends, family, or a support group about how you're feeling. Sharing your experiences can help you feel less alone.  Consider joining an asthma or anxiety support group, either in person or online.  6. Educate Yourself  Learning more about asthma and how to manage it can help you feel more in control and less anxious about potential flare-ups.  7. Seek Professional Mental Health Support  If anxiety is significantly impacting your life, consider speaking with a therapist or counselor. Cognitive Behavioral Therapy (CBT) is particularly effective for managing anxiety and can help you develop coping strategies.  8. Maintain a Healthy Lifestyle  Eat a balanced diet, get enough sleep, and avoid smoking or excessive caffeine, as these can worsen both asthma and anxiety.  9. Create an Asthma Action Plan  Having a clear plan for managing asthma symptoms can reduce anxiety about potential attacks. Work with your doctor to create a personalized plan.  10. Practice Self-Compassion  Be kind to yourself and acknowledge that managing asthma and anxiety can be challenging. Celebrate small victories and progress.  If your anxiety feels overwhelming or unmanageable, don’t hesitate to reach out to a mental health professional. You’re not alone, and there are resources and treatments available to help you feel better. | 4 | 4 | 4 | 4 | 4 | 4 | 4 |
| **Q3** | Asthma makes me worry about sudden attacks. Is there a way to stay calm? | It's completely understandable to feel anxious about sudden asthma attacks, but there are several strategies you can use to stay calm and manage your symptoms effectively:  1. Follow Your Asthma Action Plan  Work with your healthcare provider to create a personalized asthma action plan. This plan will guide you on how to manage your symptoms, when to take medications, and what to do in case of an attack. Having a clear plan can reduce anxiety.  2. Practice Breathing Techniques  Learn and practice breathing exercises, such as pursed-lip breathing or diaphragmatic breathing. These techniques can help you control your breathing during an attack and reduce panic.  3. Keep Your Medications Handy  Always carry your rescue inhaler (e.g., albuterol) with you. Knowing you have immediate access to medication can provide a sense of security and help you stay calm.  4. Identify and Avoid Triggers  Be aware of your asthma triggers (e.g., allergens, smoke, cold air) and take steps to avoid them. Reducing exposure to triggers can lower the risk of an attack.  5. Stay Informed  Educate yourself about asthma and its management. Understanding your condition can help you feel more in control and less anxious.  6. Practice Mindfulness and Relaxation  Techniques like meditation, yoga, or progressive muscle relaxation can help reduce overall anxiety and make it easier to stay calm during an asthma attack.  7. Stay Connected  Share your concerns with friends, family, or a support group. Talking about your worries can help alleviate anxiety, and having a support system can provide reassurance.  8. Monitor Your Symptoms  Use a peak flow meter to track your lung function regularly. This can help you detect early signs of worsening asthma and take action before an attack occurs.  9. Stay Positive  Focus on the fact that asthma is a manageable condition. With proper care and precautions, you can lead a normal, active life.  10. Seek Professional Help if Needed  If anxiety about asthma attacks is significantly affecting your daily life, consider speaking with a therapist or counselor. Cognitive-behavioral therapy (CBT) can be particularly helpful for managing anxiety related to chronic conditions.  By taking these steps, you can feel more prepared and in control, which can help you stay calm and confident in managing your asthma. | 4 | 4 | 4 | 4 | 4 | 4 | 4 |
|  | **Lifestyle** |  |  |  |  |  |  |  |  |
| **Q1** | How should I adjust my diet to avoid triggering asthma? | Adjusting your diet to help manage asthma involves focusing on anti-inflammatory, nutrient-rich foods while avoiding potential triggers. Here are some dietary tips to consider:  1. Focus on Anti-Inflammatory Foods  Fruits and Vegetables: Rich in antioxidants (e.g., vitamins C and E, beta-carotene), which may reduce airway inflammation. Examples include berries, oranges, spinach, kale, and bell peppers.  Omega-3 Fatty Acids: Found in fatty fish (salmon, mackerel, sardines), flaxseeds, chia seeds, and walnuts, these can help reduce inflammation.  Whole Grains: Opt for whole grains like oats, quinoa, brown rice, and whole wheat, which have anti-inflammatory properties.  Nuts and Seeds: Almonds, sunflower seeds, and pumpkin seeds provide magnesium, which may support lung function.  2. Avoid Potential Asthma Triggers  Sulfites: Found in dried fruits, wine, and processed foods, sulfites can trigger asthma in some people.  Food Additives: Preservatives like MSG, tartrazine (yellow dye), and sodium benzoate may worsen symptoms in sensitive individuals.  High-Sodium Foods: Excess salt can increase inflammation and worsen asthma symptoms.  Processed and Fried Foods: These often contain trans fats and additives that can promote inflammation.  3. Stay Hydrated  Drinking plenty of water helps keep airways moist and may reduce the risk of asthma attacks.  4. Consider Vitamin D  Low levels of vitamin D have been linked to increased asthma severity. Include fortified foods (milk, orange juice) or consider supplements if deficient.  5. Limit Dairy (if it triggers symptoms)  While dairy doesn’t directly cause asthma, some people may experience increased mucus production, which can worsen symptoms.  6. Identify Food Allergies  Common allergens like peanuts, eggs, soy, and shellfish can trigger asthma in some individuals. If you suspect a food allergy, consult an allergist for testing.  7. Maintain a Healthy Weight  Obesity can worsen asthma symptoms. A balanced diet and regular exercise can help manage weight and improve lung function.  8. Herbal Teas and Spices  Ginger and turmeric have anti-inflammatory properties and may help reduce asthma symptoms. Green tea is also a good option due to its antioxidant content.  9. Avoid Overeating  Large meals can put pressure on the diaphragm, making breathing more difficult.  10. Keep a Food Diary  Track your meals and asthma symptoms to identify potential triggers or patterns.  Always consult with your healthcare provider or a registered dietitian before making significant dietary changes, especially if you have other health conditions or are on medication. They can help tailor a plan specific to your needs. | 4 | 4 | 4 | 4 | 4 | 4 | 4 |
| **Q2** | I have pets at home. Could this worsen my asthma symptoms? | Yes, having pets at home can potentially worsen asthma symptoms, especially if you are allergic to them. Pets produce dander (tiny flakes of skin), saliva, and urine, which can all be allergens. These allergens can trigger asthma symptoms such as wheezing, coughing, shortness of breath, and chest tightness in sensitive individuals.  Here are some steps you can take to minimize the impact of pets on your asthma:  Keep Pets Out of the Bedroom: Create a pet-free zone, especially in your bedroom, to reduce exposure to allergens while you sleep.  Regular Cleaning: Vacuum frequently using a vacuum cleaner with a HEPA filter to remove pet dander from carpets and furniture. Wash bedding, curtains, and other fabrics regularly.  Groom Your Pet: Bathe and groom your pet regularly to reduce the amount of dander they shed. If possible, have someone else do this to avoid direct exposure.  Use Air Purifiers: Consider using an air purifier with a HEPA filter to reduce airborne allergens.  Wash Your Hands: After handling your pet, wash your hands to avoid transferring allergens to your face or respiratory system.  Consult Your Doctor: If your asthma symptoms worsen, consult your healthcare provider. They may recommend adjusting your asthma management plan or conducting allergy testing to confirm pet allergies.  If you find that your asthma symptoms are significantly worsened by your pets, it may be necessary to consider rehoming them, though this is often a difficult decision. Always discuss your options with your healthcare provider to find the best solution for your health and well-being. | 4 | 4 | 4 | 4 | 4 | 4 | 4 |
| **Q3** | Can I exercise? What types of exercise are best for asthma patients? | Yes, you can exercise if you have asthma, and it is generally encouraged as part of a healthy lifestyle. However, it's important to manage your asthma effectively and take precautions to avoid triggering symptoms. Here are some guidelines and types of exercise that are often recommended for asthma patients:  General Guidelines:  Consult Your Doctor: Before starting any new exercise program, consult your healthcare provider to ensure it's safe for you.  Use Your Inhaler: If prescribed, use your reliever inhaler (e.g., albuterol) 15-30 minutes before exercising to prevent exercise-induced bronchoconstriction (EIB).  Warm-Up and Cool-Down: Gradually warm up before exercising and cool down afterward to help your lungs adjust.  Monitor Conditions: Avoid exercising in cold, dry air or environments with high pollen counts, air pollution, or other triggers.  Stay Hydrated: Drink plenty of water before, during, and after exercise.  Listen to Your Body: Stop exercising and use your inhaler if you experience symptoms like wheezing, coughing, or shortness of breath.  Best Types of Exercise for Asthma Patients:  Swimming: Often recommended because the warm, moist air around pools is less likely to trigger asthma symptoms. Swimming also helps improve lung capacity and cardiovascular fitness.  Walking: A low-impact activity that can be done at your own pace. Walking indoors (e.g., on a treadmill) may be better during cold or allergy seasons.  Yoga: Combines gentle physical activity with breathing exercises, which can help improve lung function and reduce stress.  Cycling: Steady cycling, especially indoors or in mild weather, can be a good option for cardiovascular fitness.  Strength Training: Building muscle can improve overall fitness and reduce the strain on your lungs during physical activity.  Team Sports with Short Bursts of Activity: Sports like volleyball or baseball, which involve intermittent activity, may be easier to manage than continuous high-intensity sports.  Exercises to Approach with Caution:  High-Intensity or Endurance Sports: Activities like long-distance running or soccer may trigger asthma symptoms in some individuals.  Cold-Weather Sports: Skiing, ice hockey, or running in cold air can be problematic due to the dry, cold air.  Key Takeaway:  Exercise is beneficial for asthma patients, but it’s important to choose activities that suit your condition and take steps to minimize triggers. Always have your reliever inhaler on hand and work with your doctor to create a safe and effective exercise plan. | 4 | 4 | 4 | 4 | 4 | 4 | 4 |
| **Q4** | I’m overweight. Could losing weight help improve my asthma symptoms? | Yes, losing weight can potentially help improve your asthma symptoms, especially if you are overweight or obese. Here’s how weight loss might benefit your asthma:  Reduced Inflammation: Excess weight is associated with chronic low-grade inflammation, which can worsen asthma. Losing weight may help reduce this inflammation, potentially improving airway function.  Improved Lung Function: Carrying extra weight, particularly around the chest and abdomen, can restrict lung expansion and make breathing more difficult. Weight loss can alleviate this pressure, making it easier to breathe.  Better Response to Medications: Some studies suggest that obesity can reduce the effectiveness of asthma medications. Losing weight may improve your body’s response to these treatments.  Decreased Risk of Comorbidities: Obesity is often linked to other conditions like sleep apnea, acid reflux, and cardiovascular issues, which can exacerbate asthma. Weight loss can help manage or reduce these comorbidities, indirectly improving asthma control.  Enhanced Physical Activity: Being overweight can make physical activity more challenging, leading to a sedentary lifestyle. Weight loss can increase your ability to exercise, which can strengthen your respiratory muscles and improve overall lung health.  However, it’s important to approach weight loss in a healthy and sustainable way. Crash diets or extreme exercise regimens can sometimes worsen asthma symptoms. Consult your healthcare provider or a dietitian to create a personalized plan that addresses both your weight and asthma management.  If you have asthma, always follow your prescribed treatment plan and discuss any changes in your symptoms with your doctor. | 4 | 4 | 4 | 4 | 4 | 4 | 4 |
| **Q5** | As an asthma patient, what should I pay attention to in daily life? | As an asthma patient, managing your condition effectively in daily life is crucial to prevent flare-ups and maintain a good quality of life. Here are some key things to pay attention to:  1. Follow Your Asthma Action Plan  Work with your healthcare provider to create a personalized asthma action plan. This plan should include:  Daily management strategies.  How to recognize worsening symptoms.  Steps to take during an asthma attack.  When to seek emergency care.  2. Take Medications as Prescribed  Use controller medications (e.g., inhaled corticosteroids) daily to reduce inflammation and prevent symptoms.  Keep rescue medications (e.g., short-acting beta-agonists) on hand for quick relief during flare-ups.  Ensure you use inhalers correctly. Ask your doctor or pharmacist for a demonstration if needed.  3. Identify and Avoid Triggers  Common asthma triggers include:  Allergens: Pollen, dust mites, pet dander, mold.  Irritants: Smoke, strong odors, air pollution, cold air.  Infections: Colds, flu, or respiratory infections.  Exercise: Use a pre-exercise inhaler if recommended by your doctor.  Weather changes: Cold, dry air or sudden temperature changes.  Take steps to minimize exposure to triggers, such as using air purifiers, washing bedding frequently, and avoiding smoking or secondhand smoke.  4. Monitor Your Symptoms  Keep track of your symptoms and peak flow readings (if recommended by your doctor).  Recognize early warning signs of an asthma attack, such as coughing, wheezing, shortness of breath, or chest tightness.  5. Maintain a Healthy Lifestyle  Exercise regularly: Choose asthma-friendly activities like swimming or walking, and always warm up before exercising.  Eat a balanced diet: Focus on anti-inflammatory foods like fruits, vegetables, and whole grains.  Stay hydrated: Drinking water can help keep your airways moist.  Manage stress: Stress can worsen asthma symptoms, so practice relaxation techniques like deep breathing or meditation.  6. Get Vaccinated  Stay up-to-date on vaccinations, including the flu shot and pneumonia vaccine, to reduce the risk of respiratory infections that can trigger asthma.  7. Keep Your Environment Clean  Regularly clean your home to reduce dust, mold, and pet dander.  Use hypoallergenic bedding and pillow covers.  Avoid using strong cleaning products or perfumes that may irritate your airways.  8. Be Prepared for Emergencies  Always carry your rescue inhaler with you.  Know when to seek emergency care (e.g., if symptoms don't improve after using a rescue inhaler, or if you have severe difficulty breathing).  9. Regular Check-Ups  Visit your healthcare provider regularly to monitor your asthma and adjust your treatment plan as needed.  10. Educate Yourself and Others  Learn as much as you can about asthma and how to manage it.  Inform family, friends, and coworkers about your condition so they can assist you in case of an emergency.  By taking these steps, you can better control your asthma and reduce the risk of severe symptoms or attacks. Always consult your healthcare provider for personalized advice. | 4 | 4 | 4 | 4 | 4 | 4 | 4 |
| **Q6** | My asthma symptoms often worsen during seasonal changes. Besides increasing my medication, what else can I do to alleviate the symptoms? | I'm not a doctor, but I can offer some general suggestions that might help you manage your asthma symptoms during seasonal changes. Always consult your healthcare provider before making any changes to your routine. Here are some tips:  Monitor Air Quality: Check daily air quality forecasts and pollen counts. On days when pollution or pollen levels are high, try to stay indoors as much as possible.  Keep Windows Closed: During high pollen seasons, keep windows and doors closed to prevent allergens from entering your home. Use air conditioning if needed.  Use an Air Purifier: A HEPA air purifier can help reduce indoor allergens like pollen, dust, and pet dander.  Shower and Change Clothes: After being outdoors, shower and change your clothes to remove pollen and other allergens from your skin and hair.  Stay Hydrated: Drinking plenty of water can help keep your airways moist and reduce irritation.  Avoid Triggers: Identify and avoid specific triggers that worsen your asthma, such as smoke, strong odors, or cold air.  Wear a Mask: If you need to go outside during high pollen or pollution days, consider wearing a mask to filter out irritants.  Practice Breathing Exercises: Techniques like pursed-lip breathing or diaphragmatic breathing can help improve lung function and reduce symptoms.  Maintain a Clean Home: Regularly clean your living space to reduce dust mites, mold, and pet dander. Wash bedding in hot water weekly.  Stay on Top of Allergies: If allergies contribute to your asthma, consider talking to your doctor about allergy medications or immunotherapy.  Exercise Indoors: If outdoor exercise triggers your symptoms, try indoor activities like yoga, swimming, or using a treadmill.  Stay Warm in Cold Weather: Cold air can trigger asthma symptoms. Cover your nose and mouth with a scarf when going outside in chilly weather.  Follow Your Asthma Action Plan: Ensure you have an up-to-date asthma action plan from your doctor and follow it closely.  Stay Informed: Keep track of your symptoms and triggers in a journal to share with your doctor during check-ups.  Always consult your healthcare provider for personalized advice and to ensure these strategies are safe and appropriate for your specific condition. | 4 | 4 | 4 | 4 | 4 | 4 | 4 |
