# Supplementary material for: Assessing the Role of Large Language Models Between ChatGPT and DeepSeek in Asthma Education for Bilingual Individuals: Comparative Study
Source: JMIR Med Inform. 2025 Aug 13;13:e65365. doi: 10.2196/65365 (PMC12349887; doi:10.2196/65365)
Supplement: Multimedia Appendix 4 [file medinform-v13-e65365-s004.doc]

**Supplementary Table 4. DeepSeek -3’s Responses to Asthma-Related Inquiries in a Chinese-Language Context**

| **NO.** | **Questions** | **Responses** | **Consistency** | **Completeness** | **Potential Bias** | **Reasoning Ability** | **Comprehension** | **Reliability** | **Safety** |
| --- | --- | --- | --- | --- | --- | --- | --- | --- | --- |
|  | **Basic Definitions** |  |  |  |  |  |  |  |  |
| **Q1** | 我今年25岁，最近咳嗽和胸闷，早上和夜间尤其明显，吃了阿莫西林也没效果，这会不会是哮喘？ | 咳嗽和胸闷在早上和夜间加重，且抗生素（如阿莫西林）无效，确实可能与哮喘有关。哮喘的典型症状包括：  1、反复咳嗽，尤其在夜间或清晨。  2、胸闷或胸部紧迫感。  3、喘息或呼吸时有哨音。  4、呼吸困难，尤其在活动后。  不过，其他疾病如慢性支气管炎、胃食管反流病（GERD）或过敏性鼻炎也可能引发类似症状。建议你尽快就医，医生可能会通过以下方式确诊：  肺功能测试：评估气道阻塞情况。  峰流速监测：记录呼气峰流速的变化。  过敏测试：排查过敏原。  胸部X光：排除其他肺部疾病。  在确诊前，避免接触可能的诱因（如烟雾、冷空气、过敏原等），并记录症状变化，以便医生更好地判断。 | 4 | 4 | 4 | 4 | 4 | 4 | 4 |
| **Q2** | 哮喘这种病常见吗？我是不是得了它？ | 哮喘是一种相对常见的慢性呼吸道疾病，全球范围内有数亿人受其影响。它主要表现为反复发作的喘息、气短、胸闷和咳嗽，尤其在夜间或清晨症状可能加重。哮喘的严重程度因人而异，有些人症状轻微且偶尔发作，而有些人则可能频繁发作且症状较重。  你是否得了哮喘？  要确定你是否患有哮喘，需要结合症状、病史和医学检查来综合判断。以下是一些常见的哮喘症状：  1、喘息：呼吸时出现高音调的哨音，尤其在呼气时明显。  2、气短：感觉呼吸费力或无法深呼吸。  3、胸闷：胸部有压迫感或不适。  4、咳嗽：尤其在夜间或清晨加重，可能是干咳或伴有痰。  如果你有这些症状，尤其是反复发作或与特定诱因（如过敏原、冷空气、运动等）相关，建议尽早就医。医生可能会通过以下方式帮助你确诊：  肺功能测试：评估你的呼吸功能。  过敏测试：确定是否存在诱发哮喘的过敏原。  病史询问：了解症状的频率、严重程度及家族史。  需要注意的事项  哮喘是一种慢性病，但通过规范治疗和生活方式调整，大多数患者可以很好地控制症状。  如果你怀疑自己可能患有哮喘，不要自行用药，应及时就医，避免延误诊断和治疗。  希望这些信息对你有帮助！如果有更多疑问，建议咨询专业医生。 | 4 | 4 | 4 | 4 | 4 | 4 | 4 |
| **Q3** | 我怀疑自己得了哮喘，怎么才能确诊？ | 如果你怀疑自己得了哮喘，建议尽快就医进行专业诊断。以下是常见的诊断步骤：  1、病史询问：医生会详细了解你的症状、发作频率、诱因及家族病史。  2、体格检查：医生会听诊你的呼吸音，检查是否有哮喘的典型表现。  3、肺功能测试：  肺量计测试：测量呼出气量，判断气道是否受阻。  支气管激发试验：评估气道对刺激的敏感性。  呼气峰流速测试：监测日常呼气峰流速的变化。  4、影像学检查：如胸部X光或CT，排除其他肺部疾病。  5、过敏测试：确定过敏原是否引发哮喘。  6、排除其他疾病：医生会排除类似症状的其他疾病，如慢性阻塞性肺病或胃食管反流。  确诊后，医生会制定相应的治疗计划。如果你有呼吸困难等紧急症状，请立即就医。 | 4 | 4 | 4 | 3 | 4 | 4 | 4 |
| **Q4** | 诊断哮喘需要做哪些检查？是通过血液还是其他检查？ | 诊断哮喘通常需要结合多种检查方法，以下是一些常见的检查手段：  1、肺功能测试：  肺量计测试（Spirometry）：评估气道阻塞情况，测量呼气流量和肺容量。  支气管激发试验：检测气道对刺激物的敏感性。  呼气峰流速（PEF）监测：记录呼气峰流速的变化，帮助评估哮喘控制情况。  2、影像学检查：  胸部X光或CT扫描：排除其他肺部疾病，如肺炎或肺气肿。  3、过敏测试：  皮肤点刺试验或血液测试：确定是否存在过敏原，如尘螨、花粉等。  4、血液检查：  全血细胞计数：检查嗜酸性粒细胞是否升高。  免疫球蛋白E（IgE）水平：评估过敏反应。  5、痰液检查：  痰液分析：检测嗜酸性粒细胞或其他炎症标志物。  6、呼出气一氧化氮（FeNO）测试：  测量呼出气中的一氧化氮浓度，评估气道炎症。  7、病史和体格检查：  医生会详细询问症状、家族史，并进行体格检查。  哮喘的诊断通常不依赖单一检查，而是综合多项结果。血液检查主要用于评估过敏反应和炎症标志物，肺功能测试则是诊断哮喘的核心手段。 | 3 | 4 | 4 | 4 | 4 | 4 | 4 |
| **Q5** | 我最近咳嗽、喘气、胸闷，这些症状是不是代表我得了哮喘？ | 咳嗽、喘气和胸闷确实是哮喘的常见症状，但这些症状也可能与其他呼吸系统疾病（如支气管炎、慢性阻塞性肺病、肺炎等）或心脏问题有关。因此，仅凭这些症状无法确定是否患有哮喘。  如果你有这些症状，建议尽快就医，医生可能会通过以下方式帮助你明确诊断：  病史询问：了解症状的频率、持续时间、诱因等。  体格检查：听诊肺部是否有哮鸣音。  肺功能测试：评估气道阻塞情况。  激发试验或舒张试验：进一步确认气道反应性。  影像学检查：如胸部X光或CT，排除其他疾病。  哮喘的诊断需要专业医生的综合评估，不要自行判断或用药。如果症状严重或突然加重，请立即就医。 | 4 | 4 | 4 | 4 | 4 | 4 | 4 |
| **Q6** | 我得了哮喘，为什么医生说它可能与遗传有关？ | 哮喘是一种复杂的疾病，其发病机制涉及遗传和环境因素的共同作用。以下是医生认为哮喘可能与遗传有关的原因：  1、家族聚集性：哮喘在家族中常有聚集现象，若父母或近亲有哮喘，个体患病的风险显著增加。  2、遗传易感性：研究发现，某些基因变异与哮喘风险相关，这些基因多与免疫反应和气道炎症有关。  3、基因-环境交互作用：遗传因素可能使个体对环境触发因素（如过敏原、感染等）更为敏感，从而增加哮喘风险。  4、表观遗传学：环境因素可能通过表观遗传机制影响基因表达，进而影响哮喘的发病。  5、多基因遗传：哮喘并非由单一基因决定，而是多个基因共同作用的结果，增加了其遗传复杂性。  因此，医生认为遗传因素在哮喘发病中起重要作用，但环境因素同样不可忽视。 | 4 | 4 | 4 | 4 | 4 | 4 | 4 |
| **Q7** | 医生说我得了哮喘，为什么我的过敏原是阴性的？ | 哮喘的病因复杂，过敏只是其中之一。即使过敏原测试为阴性，仍可能因以下原因患上哮喘：  1、非过敏性哮喘：部分哮喘由感染、运动、冷空气或空气污染等非过敏因素引发。  2、未检测到的过敏原：测试可能未覆盖所有过敏原，或某些过敏原难以检测。  3、其他炎症机制：哮喘可能涉及非过敏性的炎症反应，如病毒感染或环境污染。  4、遗传与环境因素：家族史和环境暴露也可能导致哮喘，与过敏无关。  5、测试局限性：过敏原测试的准确性受多种因素影响，可能出现假阴性。  建议与医生讨论，进一步检查以明确病因并制定合适的治疗方案。 | 4 | 4 | 4 | 4 | 4 | 4 | 4 |
|  | **Clinical Features** |  |  |  |  |  |  |  |  |
| **Q1** | 哮喘的典型症状是什么？我应该怎么识别自己是不是得了哮喘？ | 哮喘的典型症状包括：  1、喘息：呼吸时伴有高音调的哨声，尤其在呼气时明显。  2、气短：感觉呼吸不畅，尤其在活动或夜间加重。  3、胸闷：胸部有压迫感或不适。  4、咳嗽：尤其在夜间或清晨，可能是干咳或伴有痰。  如何识别是否患有哮喘  1、症状模式：上述症状反复出现，尤其在夜间或清晨加重。  2、诱因：接触过敏原（如花粉、尘螨）、冷空气、运动、感染等可能引发症状。  3、缓解情况：使用支气管扩张剂后症状有所缓解。  4、病史：家族中有哮喘或过敏史可能增加患病风险。  建议：  如果怀疑自己患有哮喘，应及时就医，医生会通过肺功能测试、病史和体检来确诊，并制定合适的治疗方案。 | 4 | 4 | 4 | 4 | 4 | 4 | 4 |
| **Q2** | 除了咳嗽和喘息，哮喘还有其他不典型的症状吗 | 除了咳嗽和喘息，哮喘还可能表现出以下不典型症状：  胸闷：患者常感到胸部压迫或不适。  呼吸困难：尤其在夜间或清晨，呼吸可能变得困难。  夜间咳嗽：咳嗽在夜间或凌晨加重，影响睡眠。  运动后症状：运动可能诱发咳嗽或喘息。  疲劳：呼吸不畅导致能量消耗增加，引发疲劳。  焦虑：呼吸困难可能引发焦虑或恐慌。  睡眠障碍：夜间症状可能导致失眠或频繁醒来。  声音嘶哑：部分患者可能出现声音变化。  清嗓：频繁清嗓或感觉喉咙有异物。  胸痛：少数患者可能感到胸痛，尤其在剧烈咳嗽后。  这些症状可能与其他疾病相似，因此准确诊断需结合病史、体检和肺功能测试。如有疑虑，应及时就医。 | 4 | 4 | 4 | 4 | 4 | 4 | 4 |
| **Q3** | 哮喘是否会引起其他身体上的问题，比如头痛或者食欲不振？ | 哮喘本身主要影响呼吸系统，但可能间接引发其他身体问题，如头痛或食欲不振。以下是具体原因：  头痛：哮喘发作时，呼吸困难可能导致缺氧，进而引发头痛。此外，频繁使用某些哮喘药物（如支气管扩张剂）也可能引起头痛。  食欲不振：严重的哮喘发作可能让人感到疲惫和不适，影响食欲。长期哮喘或频繁发作还可能引发焦虑或抑郁，进一步导致食欲下降。  睡眠问题：夜间哮喘发作会干扰睡眠，导致疲劳和头痛，进而影响白天的食欲。  药物副作用：部分哮喘药物可能引起胃肠道不适，导致食欲不振。  如果出现头痛或食欲不振等症状，建议咨询医生，评估是否与哮喘或其治疗相关，并调整治疗方案。 | 4 | 4 | 4 | 4 | 4 | 4 | 4 |
| **Q4** | 如果我哮喘发作，应该会有什么样的表现？我应该怎么办？ | 哮喘发作的表现  哮喘发作时，常见的症状包括：  呼吸困难：感觉呼吸费力，尤其是呼气时。  喘息：呼吸时伴有高音调的哨声。  咳嗽：持续咳嗽，可能伴有粘液。  胸闷：胸部有压迫感或紧绷感。  呼吸急促：呼吸频率加快。  说话困难：因呼吸困难而难以完整表达。  焦虑或恐慌：因呼吸不畅感到紧张或恐慌。  应对措施  使用急救药物：  立即使用医生处方的速效吸入剂（如沙丁胺醇），通常5-10分钟内可缓解症状。  保持冷静：  尽量放松，深呼吸，避免紧张加重症状。  坐直：  坐直有助于打开气道，缓解呼吸困难。  远离诱因：  如果知道诱因（如过敏原、烟雾等），尽快远离。  监测症状：  使用峰流速计监测呼吸状况，记录数据以便就医时参考。  寻求帮助：  如果症状未缓解或加重，立即拨打急救电话或前往医院。  预防措施  定期用药：  遵医嘱使用控制药物，预防发作。  避免诱因：  识别并避免接触过敏原、冷空气等诱因。  制定行动计划：  与医生制定哮喘行动计划，明确发作时的应对步骤。  定期复查：  定期就医，调整治疗方案。  何时就医  急救药物无效。  症状迅速恶化。  峰流速值持续下降。  出现严重呼吸困难、嘴唇或指甲发紫、意识模糊等紧急情况。  总结  哮喘发作时，应立即使用急救药物并保持冷静。若症状未缓解或加重，需尽快就医。平时应遵医嘱用药，避免诱因，并定期复查，以有效控制病情。 | 4 | 4 | 4 | 4 | 4 | 4 | 4 |
| **Q5** | 为什么我白天没有问题，一到晚上就咳嗽喘憋？是哮喘的特征吗？ | 夜间咳嗽和喘憋可能是哮喘的特征之一，尤其是夜间哮喘。哮喘患者常在夜间或清晨症状加重，原因包括：  1、生理变化：夜间体内激素水平下降，气道更容易收缩。  2、体位影响：平躺时，胃酸可能反流刺激气道，或鼻腔分泌物倒流引发咳嗽。  3、过敏原暴露：卧室中的尘螨、宠物皮屑等过敏原可能引发症状。  4、空气干燥或寒冷：夜间气温下降或空气干燥可能刺激气道。  5、昼夜节律：夜间气道炎症反应可能增强，导致症状加重。  如果夜间症状频繁，建议尽快就医，医生可能通过肺功能测试等检查确认是否为哮喘，并制定相应治疗方案。 | 4 | 4 | 4 | 4 | 4 | 4 | 4 |
| **Q6** | 我经常感到胸 发紧，这是不是哮喘发作的征兆？ | 胸 发紧可能是哮喘发作的一个征兆，但也可能与其他健康问题有关。哮喘的常见症状包括：  胸 发紧或压迫感  呼吸困难  喘息  咳嗽，尤其是在夜间或清晨  然而，胸 发紧也可能由其他原因引起，如焦虑、胃食管反流病（GERD）、肌肉紧张或心脏问题等。  如果你经常感到胸 发紧，尤其是伴随其他哮喘症状，建议尽快就医，进行详细检查以确定原因。医生可能会通过肺功能测试、胸部X光或其他检查来帮助诊断。  如果确诊为哮喘，医生会制定相应的治疗计划，可能包括使用吸入器或其他药物来控制症状。如果症状突然加重或出现严重的呼吸困难，应立即寻求紧急医疗帮助。 | 4 | 4 | 4 | 4 | 4 | 4 | 4 |
|  | **Treatment** |  |  |  |  |  |  |  |  |
| **Q1** | 我怀疑自己得了哮喘，在去医院之前可以自己使用什么药物缓解症状吗？ | 如果您怀疑自己得了哮喘，建议尽快就医，而不是自行用药。哮喘症状可能包括呼吸困难、胸闷、咳嗽和喘息，严重时可能危及生命。  在就医前，可以采取以下措施缓解症状：  1、远离诱因：避免接触可能引发症状的过敏原或刺激物，如烟雾、灰尘、花粉等。  2、保持冷静：焦虑可能加重症状，尽量保持平静，缓慢深呼吸。  3、使用急救药物（如有）：如果您已被诊断为哮喘并备有急救药物（如沙丁胺醇吸入器），可按医嘱使用。  但请注意，自行用药存在风险，尤其是未经医生指导的情况下。尽快就医，接受专业诊断和治疗是最安全的选择。如果症状严重，如呼吸困难、嘴唇发紫等，应立即拨打急救电话。 | 4 | 4 | 4 | 4 | 4 | 4 | 4 |
| **Q2** | 2.我被确诊为哮喘，我想知道我应该用什么药。 | 哮喘是一种慢性炎症性气道疾病，通常需要长期管理。治疗方案应根据病情的严重程度和个体差异来制定。以下是常见的哮喘治疗药物：  1. 控制药物（长期使用）  吸入性糖皮质激素（ICS）：如布地奈德、氟替卡松等，用于减轻气道炎症，预防哮喘发作。  长效β2受体激动剂（LABA）：如沙美特罗、福莫特罗，通常与ICS联合使用，帮助放松气道平滑肌。  白三烯调节剂：如孟鲁司特，用于减轻炎症和缓解症状。  长效抗胆碱能药物（LAMA）：如噻托溴铵，用于放松气道。  生物制剂：如奥马珠单抗，适用于重度哮喘患者。  2. 缓解药物（急性发作时使用）  短效β2受体激动剂（SABA）：如沙丁胺醇、特布他林，用于快速缓解急性症状。  短效抗胆碱能药物（SAMA）：如异丙托溴铵，用于急性发作时的辅助治疗。  3. 其他药物  服糖皮质激素：如泼尼松，用于严重急性发作时的短期治疗。  茶碱类药物：如氨茶碱，用于辅助治疗，但需注意副作用。  4. 个体化治疗  治疗方案应根据哮喘的严重程度、发作频率和个体反应进行调整。建议定期随访，评估病情并调整用药。  重要提示：  遵医嘱用药：不要自行调整药物剂量或停药。  正确使用吸入器：确保掌握吸入器的正确使用方法。  避免诱因：如过敏原、冷空气、烟雾等，减少哮喘发作。  请务必咨询医生或呼吸科专家，制定适合你的个性化治疗方案。 | 4 | 4 | 4 | 4 | 4 | 4 | 4 |
| **Q3** | 医生让我吸入布地奈德，我能知道这个药会有什么副作用吗？ | 布地奈德是一种吸入型糖皮质激素，常用于治疗哮喘和慢性阻塞性肺病（COPD）。虽然它通常安全有效，但仍可能引发一些副作用。以下是一些常见的副作用：  常见副作用：  腔和咽喉刺激：可能导致喉咙痛、声音嘶哑或 腔干燥。  腔真菌感染（鹅 疮）：使用后漱 可降低风险。  咳嗽：部分患者可能感到轻微咳嗽或刺激。  较少见的副作用：  头痛：少数人可能会感到头痛。  恶心：偶尔会出现恶心感。  呼吸道感染：可能增加上呼吸道感染的风险。  罕见但严重的副作用：  过敏反应：如皮疹、瘙痒、呼吸困难等，需立即就医。  视力问题：长期使用可能增加白内障或青光眼的风险。  骨质疏松：长期高剂量使用可能影响骨密度。  注意事项：  使用后漱 ：减少 腔和咽喉的副作用。  定期复查：长期使用需定期检查，监测潜在副作用。  遵医嘱：不要自行调整剂量或停药。  如有不适，及时咨询医生。 | 4 | 4 | 4 | 4 | 4 | 4 | 4 |
| **Q4** | 我使用了布地奈德福莫特罗后，心跳很快，觉得不舒服，这是不是正常的反应？ | 使用布地奈德福莫特罗后心跳加快并感到不适，可能是药物的副作用之一。福莫特罗是一种长效β2受体激动剂，虽然主要用于扩张支气管，但也可能影响心脏，导致心率加快、心悸等反应。  可能的原因：  药物副作用：福莫特罗可能刺激心脏β受体，引发心率加快。  剂量问题：剂量过高可能增加副作用风险。  个体差异：部分人对药物更敏感，容易出现不适。  建议：  咨询医生：如果症状明显或持续，应及时联系医生，可能需要调整剂量或更换药物。  监测症状：记录心跳加快的频率和持续时间，帮助医生评估情况。  避免诱因：减少咖啡因等可能加重心跳加快的物质摄入。  注意：  如果出现胸痛、呼吸困难或严重心悸，应立即就医。  总之，心跳加快可能是药物的正常反应，但也需警惕，建议与医生沟通以确保安全。 | 4 | 4 | 4 | 4 | 4 | 4 | 4 |
| **Q5** | 我爸爸也有哮喘，使用沙美特罗替卡松后嗓子痛、声音嘶哑，这是不是药物的副作用？ | 沙美特罗替卡松是一种常用于治疗哮喘的药物，包含长效β2受体激动剂（沙美特罗）和吸入性糖皮质激素（替卡松）。虽然它通常耐受性良好，但确实存在一些副作用。  嗓子痛和声音嘶哑可能是吸入性糖皮质激素的局部副作用，尤其是如果使用后没有正确漱 。药物残留在喉咙或 腔可能引发刺激。建议您爸爸在使用后彻底漱 ，以减少药物残留。  如果症状持续或加重，建议咨询医生，可能需要调整用药方式或剂量。 | 4 | 4 | 4 | 4 | 4 | 4 | 4 |
| **Q6** | 6.有没有新的治疗方法，能让我的哮喘不再频繁发作？ | 哮喘是一种慢性炎症性疾病，目前尚无根治方法，但通过合理的治疗和管理，可以有效控制症状，减少发作频率。以下是一些新的治疗方法和策略，可能对你有帮助：  1. 生物制剂  抗IgE单抗（如奥马珠单抗）：适用于中重度过敏性哮喘，通过阻断IgE抗体减少过敏反应。  抗IL-5单抗（如美泊利单抗、雷珠单抗）：针对嗜酸性粒细胞增多的哮喘患者，减少气道炎症。  抗IL-4/IL-13单抗（如杜匹鲁单抗）：适用于Th2型炎症的哮喘患者，改善症状和肺功能。  2. 支气管热成形术  这是一种非药物治疗，通过热能减少气道平滑肌，降低气道收缩能力，适用于严重哮喘患者。  3. 新型吸入药物  超细颗粒吸入剂：提高药物在肺部的沉积率，增强疗效。  三联吸入疗法：结合吸入性糖皮质激素、长效β2受体激动剂和长效抗胆碱能药物，适用于中重度哮喘患者。  4. 个性化治疗  通过基因检测和生物标志物分析，制定个性化治疗方案，提高疗效。  5. 智能吸入器  配备传感器的智能吸入器可记录用药情况，提醒患者按时用药，帮助医生调整治疗方案。  6. 生活方式和环境管理  避免过敏原：如尘螨、花粉、宠物皮屑等。  空气净化器：减少室内空气污染物。  健康饮食和锻炼：增强免疫力，改善肺功能。  7. 呼吸训练  Buteyko呼吸法和Pranayama瑜伽呼吸法可帮助控制呼吸，减少症状。  8. 定期随访和监测  定期检查肺功能和哮喘控制情况，及时调整治疗方案。  9. 心理支持  哮喘与心理压力相关，心理咨询或放松训练有助于减少发作。  10. 新型 服药物  DPP-4抑制剂和PDE4抑制剂等新型 服药物正在研究中，可能为哮喘治疗提供新选择。  重要提示：  在尝试新疗法前，务必咨询医生，确保治疗方案适合你的病情。  通过这些新方法和综合管理，哮喘发作频率有望显著减少，生活质量得到改善。 | 4 | 4 | 4 | 4 | 4 | 4 | 4 |
| **Q7** | 治疗哮喘吸入药物和 服药物有什么区别？哪个更有效？ | 治疗哮喘的吸入药物和 服药物在作用方式、起效时间和副作用等方面存在显著区别。  1. 作用方式  吸入药物：直接作用于呼吸道，迅速缓解症状或控制炎症。  服药物：通过全身循环到达呼吸道，作用较慢。  2. 起效时间  吸入药物：起效快，适合急性发作。  服药物：起效较慢，通常用于长期控制。  3. 副作用  吸入药物：局部副作用如 腔感染、声音嘶哑，全身副作用较少。  服药物：全身副作用较多，如骨质疏松、血糖升高等。  4. 使用方便性  吸入药物：需要正确使用吸入器，操作要求较高。  服药物：使用简单，但需注意剂量和频率。  5. 有效性  吸入药物：通常更有效，因为药物直接作用于呼吸道，副作用较少。  服药物：在吸入药物效果不佳时作为补充，或用于严重哮喘。  总结  吸入药物通常是首选，因其起效快、副作用少且直接作用于呼吸道。 服药物则多用于长期控制或严重哮喘。具体选择应根据患者情况和医生建议。 | 4 | 4 | 4 | 4 | 4 | 4 | 4 |
| **Q8** | 我正在使用治疗哮喘的药物，但我今天忘记用药了，应该怎么补救？ | 如果你今天忘记使用治疗哮喘的药物，可以采取以下步骤：  立即用药：如果距离下次用药时间还早，尽快补上漏服的剂量。  跳过剂量：如果已经接近下次用药时间，跳过漏服的剂量，按原计划继续用药，避免双倍剂量。  咨询医生或药剂师：如果不确定如何处理，联系医生或药剂师获取建议。  设置提醒：使用手机提醒或药盒帮助记住按时服药。  监测症状：留意哮喘症状，如有异常（如呼吸困难），立即就医。  避免诱因：尽量避免接触可能引发哮喘的因素，如过敏原或冷空气。  及时补药并遵循医生建议是关键，如有疑问或症状加重，尽快联系医疗专业人员。 | 4 | 4 | 4 | 4 | 4 | 4 | 4 |
| **Q9** | 如果我不想一直依赖药物，能通过饮食或运动来控制哮喘吗？ | 虽然药物在哮喘管理中至关重要，但饮食和运动也可以作为辅助手段，帮助你更好地控制症状。以下是一些建议：  饮食  1.  抗炎食物：多摄入富含抗氧化剂和抗炎成分的食物，如水果、蔬菜、坚果和鱼类。  Omega-3脂肪酸：鲑鱼、亚麻籽等食物有助于减轻炎症。  维生素D：适量晒太阳或食用富含维生素D的食物，如鱼类和强化乳制品。  避免过敏原：如果你对某些食物过敏，避免它们可以减少哮喘发作的风险。  运动  有氧运动：如游泳、步行和骑自行车，有助于增强心肺功能。  呼吸练习：如瑜伽和深呼吸练习，有助于改善呼吸控制。  适度运动：避免过度运动，尤其是在寒冷或空气污染严重的环境中。  其他建议  控制体重：保持健康体重有助于减轻哮喘症状。  避免诱因：如烟雾、花粉和宠物皮屑等。  定期监测：使用峰值流量计监测肺功能，及时调整管理计划。  重要提示  咨询医生：在调整饮食或运动计划前，务必咨询医生。  药物依赖：不要擅自停药，药物在哮喘管理中仍然非常重要。  通过这些方法，你可以在一定程度上减少对药物的依赖，但仍需在医生指导下进行。 | 4 | 4 | 4 | 4 | 4 | 4 | 4 |
| **Q10** | 诊断哮喘后医生让我吸入激素，我以后会不会有激素依赖？ | 吸入激素是哮喘治疗的主要手段，能有效控制气道炎症，减少发作。以下是关键点：  局部作用：吸入激素主要在肺部起作用，全身吸收少，副作用较小。  不会成瘾：吸入激素不会导致依赖，长期使用是为了控制炎症，而非身体对其产生依赖。  遵医嘱使用：医生会根据病情调整剂量，患者不应自行停药或更改剂量。  副作用：常见副作用包括 腔念珠菌感染和声音嘶哑，使用后漱 可降低风险。  定期复查：定期随访有助于评估疗效和调整治疗方案。总之，吸入激素是安全有效的哮喘控制方法，不会导致依赖，但需遵医嘱使用并定期复查。 | 4 | 4 | 4 | 4 | 4 | 4 | 4 |
|  | **Assessment and Management** |  |  |  |  |  |  |  |  |
| **Q1** | 哮喘是否会影响我的寿命？我应该如何管理病情以避免严重后果？ | 哮喘是一种慢性炎症性气道疾病，虽然无法根治，但通过科学管理，大多数患者可以控制症状，过上正常生活。以下是关键点：  哮喘对寿命的影响  控制良好：若哮喘得到有效控制，通常不会显著影响寿命。  控制不佳：严重或未控制的哮喘可能增加急性发作风险，进而影响寿命。  哮喘管理策略  遵循医嘱用药：  控制药物：如吸入性糖皮质激素，需长期使用。  急救药物：如短效β2受体激动剂，用于急性发作。  定期复诊：  定期检查肺功能，评估病情，及时调整治疗方案。  避免诱因：  识别并避免过敏原（如尘螨、花粉）和刺激物（如烟雾、冷空气）。  监测症状：  使用峰流速仪监测肺功能，记录哮喘日记，发现异常及时就医。  健康生活方式：  保持均衡饮食、适量运动、充足睡眠，增强免疫力。  应急计划：  与医生制定急性发作时的应对方案，包括药物使用和紧急联系方式。  何时就医  症状加重或频繁发作。  急救药物使用增加或效果减弱。  出现呼吸困难、胸痛等严重症状。  总结  通过规范治疗和科学管理，哮喘患者可以有效控制病情，减少急性发作，维持正常生活。定期复诊、避免诱因、健康生活方式和应急准备是管理哮喘的关键。  如有疑问或症状变化，及时咨询医生。 | 4 | 4 | 4 | 4 | 4 | 4 | 4 |
| **Q2** | 我有哮喘，但还是抽烟了，这会加重病情吗？ | 是的，抽烟会显著加重哮喘病情。以下是具体原因：  刺激呼吸道：烟草烟雾中的化学物质会直接刺激呼吸道，引发炎症和肿胀，导致哮喘症状恶化。  增加黏液分泌：吸烟会促使呼吸道产生更多黏液，进一步阻塞气道，加重呼吸困难。  降低肺功能：长期吸烟会损害肺功能，使哮喘患者更难控制症状。  减弱药物效果：吸烟可能降低哮喘药物的疗效，增加急性发作的风险。  增加发作频率和严重性：吸烟者更容易频繁和严重地发作哮喘，甚至可能引发危及生命的急性发作。  长期损害：吸烟还会增加患慢性阻塞性肺病（COPD）等严重呼吸系统疾病的风险。  建议：为了健康，强烈建议你戒烟，并咨询医生制定合适的治疗和管理计划。戒烟不仅能改善哮喘控制，还能提升整体健康。 | 4 | 4 | 4 | 4 | 4 | 4 | 4 |
| **Q3** | 哮喘发作时有哪些症状，我应该什么时候去医院治疗？ | 哮喘发作时的症状包括：  呼吸困难：感觉呼吸费力或气短。  喘息：呼吸时伴有高音调的哨音。  咳嗽：尤其在夜间或清晨加重，可能伴有痰或无痰。  胸闷：胸部有压迫感或不适。  呼吸急促：呼吸频率加快。  何时就医：  症状加重：如喘息、呼吸困难或胸闷加剧。  药物无效：使用急救吸入器后症状未缓解或迅速复发。  严重呼吸困难：说话困难或嘴唇、指甲发紫。  心跳加速：心率明显加快。  意识模糊：出现嗜睡或意识不清。  紧急情况：  无法缓解的严重呼吸困难。  急救药物无效。  意识丧失。  此时应立即拨打急救电话或前往急诊室。 | 4 | 4 | 4 | 4 | 4 | 4 | 4 |
| **Q4** | 如果我得了哮喘，怎么评估自己病情的严重程度？有自测方法吗？ | 评估哮喘病情的严重程度需要结合症状频率、肺功能测试和日常活动的影响。以下是一些自测方法：  1. 症状频率  日间症状：每周出现几次喘息、咳嗽、胸闷或气短？  夜间症状：是否因哮喘症状夜间醒来？  症状持续时间：症状持续多久？是否频繁发作？  2. 使用峰流速仪  峰流速值（PEF）：通过峰流速仪测量呼气峰流速，记录早晚的数值，观察波动情况。若早晚差异大或数值低于个人最佳值，可能表明哮喘控制不佳。  3. 药物使用情况  急救药物：每周使用短效β2受体激动剂（如沙丁胺醇）的次数是多少？频繁使用可能意味着哮喘控制不良。  4. 活动受限  日常影响：哮喘是否影响日常活动、运动或工作？活动受限程度越高，病情可能越严重。  5. 哮喘控制问卷  ACT（哮喘控制测试）：通过回答5个问题，评估哮喘控制情况。得分越低，控制越差。  6. 急性发作频率  发作次数：过去一年中有多少次急性发作？发作越频繁，病情可能越严重。  7. 肺功能测试  FEV1：通过肺功能测试评估第一秒用力呼气量（FEV1），FEV1越低，病情可能越严重。  8. 就医频率  急诊或住院：是否因哮喘频繁就医或住院？频率越高，病情可能越严重。  自测注意事项：  记录症状：建议记录症状、峰流速值和药物使用情况，帮助医生评估病情。  定期复诊：即使症状轻微，也应定期复诊，医生会根据情况调整治疗方案。  何时就医：  若症状加重、峰流速值持续下降或急救药物效果不佳，应立即就医。  哮喘管理需要与医生密切合作，制定个性化治疗计划。 | 4 | 4 | 4 | 4 | 4 | 4 | 4 |
| **Q5** | 我是一个哮喘患者，每天我都在用药，病情稳定后，我可以停药吗？ | 哮喘是一种慢性炎症性疾病，通常需要长期管理。即使病情稳定，也不建议自行停药，因为突然停药可能导致症状复发或加重。以下是一些关键点：  遵医嘱用药：是否停药应由医生根据你的病情决定。医生可能会逐步调整药物剂量，而不是立即停药。  定期复查：即使感觉良好，也应定期复诊，医生会根据你的情况评估是否需要调整治疗方案。  避免诱因：继续避免已知的哮喘诱因，如过敏原、烟雾等，以减少发作风险。  应急药物：即使病情稳定，也应随身携带应急药物（如短效支气管扩张剂），以防突发症状。  生活方式：保持健康的生活方式，如适量运动、均衡饮食和充足睡眠，有助于控制哮喘。  总之，哮喘管理需要长期坚持，停药前务必咨询医生，确保安全。 | 4 | 4 | 4 | 4 | 4 | 4 | 4 |
| **Q6** | 我怎样使用峰流速计来监测我的哮喘症状？什么时候使用它最有效？ | 使用峰流速计监测哮喘症状的步骤如下：  准备：确保峰流速计清洁，指针归零。  站立或坐直：保持身体直立，确保呼吸顺畅。  深吸气：用嘴深吸一 气，尽量充满肺部。  含住吹嘴：用嘴唇紧含吹嘴，避免漏气。  快速呼气：尽可能快速、用力地呼气。  记录读数：记下指针指示的数值。  重复：通常需重复三次，取最高值作为最终结果。  使用时机  每日监测：建议早晚各测一次，了解日常变化。  症状加重时：如感到呼吸不畅或哮喘症状加剧，及时测量。  用药前后：用药前后测量，评估药物效果。  医生建议：根据医生要求进行定期监测。  注意事项  记录数据：将每次测量结果记录下来，方便医生参考。  清洁设备：定期清洗吹嘴，保持卫生。  遵循医嘱：根据医生建议调整监测频率和方式。  通过这些步骤和时机，峰流速计能帮助你有效监测哮喘症状，及时调整治疗方案。 | 4 | 4 | 4 | 4 | 4 | 4 | 4 |
| **Q7** | 哪些因素会影响哮喘的预后，我该如何避免？ | 哮喘的预后受多种因素影响，主要包括：  遗传因素：家族中有哮喘或过敏史的人，预后可能较差。  环境因素：空气污染、吸烟、过敏原（如尘螨、花粉、宠物皮屑）等会加重症状。  治疗依从性：不规律用药或未按医嘱治疗会影响病情控制。  合并症：如肥胖、鼻炎、胃食管反流等可能使哮喘更难控制。  心理因素：压力和焦虑可能诱发或加重哮喘发作。  感染：呼吸道感染（如感冒、流感）可能引发急性发作。  如何改善预后：  规范治疗：遵医嘱使用控制药物（如吸入激素）和急救药物（如短效β2受体激动剂）。  避免诱因：减少接触过敏原和刺激物，保持室内空气清洁，避免吸烟。  定期随访：定期复查，调整治疗方案。  健康生活方式：保持健康体重，适度锻炼，避免剧烈运动。  心理调节：通过放松训练等方式缓解压力。  预防感染：接种流感疫苗和肺炎疫苗，注意个人卫生。  通过规范治疗和生活方式调整，可以有效控制哮喘，改善预后。 | 4 | 4 | 4 | 4 | 4 | 4 | 4 |
| **Q8** | 我患有哮喘2年了，我现在已经没有喘息的症状了，但活动后还有些气短，应该怎么做？ | 首先，恭喜你哮喘症状有所改善，但活动后仍感到气短，说明病情尚未完全控制。以下是一些建议：  1. 继续用药  控制药物：如吸入性糖皮质激素（ICS）或长效β2受体激动剂（LABA），需长期使用，即使无症状也不应擅自停药。  急救药物：如短效β2受体激动剂（SABA），随身携带以备不时之需。  2. 定期复诊  定期与医生沟通，评估病情并调整治疗方案。  3. 监测肺功能  使用峰流速仪监测呼气峰流速（PEF），帮助了解病情变化。  4. 适度运动  选择低强度运动，如散步、游泳或瑜伽，逐步增加强度，避免诱发气短。  5. 避免诱因  远离过敏原、冷空气、烟雾等可能引发哮喘的因素。  6. 呼吸训练  学习腹式呼吸或深呼吸，增强肺功能。  7. 保持健康生活方式  均衡饮食、充足睡眠、减压，避免过度疲劳。  8. 心理调节  哮喘可能带来焦虑，必要时寻求心理支持。  9. 紧急处理  如气短加重或急救药物无效，立即就医。  10. 个性化管理  与医生合作，制定适合你的管理计划。  总之，哮喘需要长期管理，坚持用药、定期复诊、适度运动并避免诱因是关键。如有不适，及时就医。 | 4 | 4 | 4 | 4 | 4 | 4 | 4 |
| **Q9** | 我有哮喘，能彻底治愈吗？以后还能正常生活吗？ | 哮喘是一种慢性炎症性疾病，目前尚无法彻底治愈，但通过适当的治疗和管理，大多数患者可以过上正常的生活。以下是一些关键点：  药物治疗  控制药物：如吸入性糖皮质激素，用于长期控制炎症，减少发作。  缓解药物：如短效β2受体激动剂，用于急性发作时快速缓解症状。  生物制剂：针对特定类型哮喘的新型治疗药物。  生活方式管理  避免诱因：如过敏原、烟雾、冷空气等。  健康饮食：均衡饮食有助于增强免疫力。  适量运动：在医生指导下进行，如游泳、散步等。  定期监测  肺功能测试：定期检查以评估病情。  症状日记：记录症状和用药情况，帮助医生调整治疗方案。  心理支持  心理咨询：帮助应对疾病带来的心理压力。  支持团体：与其他患者交流经验，获得支持。  紧急应对  急救药物：随身携带，以备不时之需。  紧急计划：与医生制定急性发作时的应对方案。  长期预后  儿童哮喘：部分患儿在青春期后症状可能减轻或消失。  成人哮喘：需长期管理，但通过有效治疗可以控制症状。  虽然哮喘无法根治，但通过规范治疗和良好的自我管理，大多数患者可以正常生活。建议与医生保持沟通，制定个性化的治疗计划。 | 4 | 4 | 4 | 4 | 4 | 4 | 4 |
| **Q10** | 我是一个哮喘患者，我的病情控制得不错，为什么感觉身体状况并没有好转？ | 虽然你的哮喘病情得到了控制，但身体状况未明显改善可能与以下因素有关：  其他健康问题：可能存在未被发现的其他健康问题，如过敏、鼻窦炎、胃食管反流或睡眠呼吸暂停等，影响整体健康。  药物副作用：长期使用哮喘药物，尤其是 服类固醇，可能引发疲劳、体重增加等副作用，影响身体状态。  缺乏运动：哮喘患者可能因担心诱发症状而减少运动，导致体能下降、体重增加或肌肉无力。  心理因素：焦虑、抑郁等心理问题可能影响身体感受，即使哮喘控制良好，仍可能感到不适。  生活方式：不健康的饮食、睡眠不足或吸烟等习惯也会影响整体健康。  环境因素：空气污染、过敏原等环境因素可能持续刺激呼吸道，引发不适。  哮喘控制不完全：即使症状减轻，气道炎症可能仍然存在，导致身体不适。  建议：  与医生讨论，评估是否存在其他健康问题或药物副作用。  逐步增加运动，提升体能。  关注心理健康，必要时寻求专业帮助。  改善生活方式，如健康饮食、充足睡眠和戒烟。  定期复诊，确保哮喘得到最佳控制。  如果症状持续或加重，务必及时就医。 | 4 | 4 | 4 | 4 | 4 | 4 | 4 |
| **Q11** | 医生说我是哮喘，如果我的症状在使用药物后没有缓解，我该如何判断是否需要再就医？ | 如果您的哮喘症状在使用药物后没有缓解，建议您考虑以下几点来判断是否需要再次就医：  症状持续或加重：如果症状如喘息、咳嗽、胸闷或呼吸困难没有改善，甚至加重，应及时就医。  药物效果不佳：若按医嘱使用药物后效果不明显，或需要频繁使用急救药物（如短效β2受体激动剂），应尽快联系医生。  夜间症状：若夜间因哮喘症状频繁醒来，表明病情控制不佳，需就医调整治疗方案。  活动受限：如果日常活动因哮喘症状受到明显限制，也应及时就医。  峰值流速下降：使用峰值流速计监测时，若数值持续低于个人最佳值的80%，需尽快就医。  紧急情况：出现严重呼吸困难、说话困难、嘴唇或指甲发紫等症状时，应立即前往急诊。  建议：  记录症状：详细记录症状变化和药物使用情况，就诊时提供给医生。  定期复诊：即使症状稳定，也应定期复诊，确保病情得到良好控制。  总之，若药物效果不佳或症状加重，应及时就医，避免延误治疗。 | 4 | 4 | 4 | 4 | 4 | 4 | 4 |
|  | **Differential Diagnosis** |  |  |  |  |  |  |  |  |
| **Q1** | 除了哮喘，还有哪些其他病症会让我出现咳嗽和喘息？何时就医？ | 除了哮喘，以下病症也可能引发咳嗽和喘息：  慢性阻塞性肺疾病（COPD）  症状：持续咳嗽、咳痰、喘息、呼吸困难，尤其在活动时加重。  常见于：长期吸烟者或长期暴露于有害气体的人群。  支气管炎  急性支气管炎：通常由感染引起，伴有咳嗽、喘息、痰多，可能伴有发热。  慢性支气管炎：长期咳嗽、咳痰，喘息在急性加重期更为明显。  肺炎  症状：咳嗽、喘息、发热、胸痛、呼吸困难，痰可能带血或呈黄绿色。  常见于：细菌、病毒或真菌感染。  心力衰竭  症状：夜间或平卧时咳嗽、喘息加重，伴有呼吸困难、下肢水肿、疲劳。  常见于：心脏功能不全的患者。  过敏性鼻炎或鼻窦炎  症状：咳嗽、喘息，常伴有鼻塞、流涕、打喷嚏，可能引发哮喘样症状。  常见于：过敏体质者。  胃食管反流病（GERD）  症状：咳嗽、喘息，尤其在夜间或饭后加重，伴有烧心、反酸。  常见于：胃酸反流刺激气道。  肺栓塞  症状：突发呼吸困难、咳嗽、喘息，可能伴有胸痛、咯血。  常见于：长期卧床、手术后或有血栓风险者。  肺癌  症状：持续咳嗽、喘息、咳血、胸痛、体重下降。  常见于：长期吸烟者或有肺癌家族史者。  支气管扩张  症状：慢性咳嗽、大量咳痰、喘息，反复感染。  常见于：先天性疾病或反复呼吸道感染患者。  过敏性肺炎  症状：咳嗽、喘息、发热、乏力，接触过敏原后加重。  常见于：长期暴露于有机粉尘或霉菌者。  如果咳嗽和喘息持续或加重，或伴有胸痛、咳血、呼吸困难等症状，应及时就医。医生会根据病史、体检和检查（如肺功能测试、胸部X光等）进行诊断和治疗。 | 3 | 3 | 4 | 4 | 4 | 4 | 4 |
| **Q2** | 我有慢性咳嗽和气短，医生怀疑是哮喘，是否可能是其他疾病？ | 慢性咳嗽和气短确实是哮喘的常见症状，但也可能与其他疾病有关。以下是一些可能的其他原因：  慢性阻塞性肺疾病（COPD）：常见于长期吸烟者，症状与哮喘相似，包括咳嗽、气短和喘息。  胃食管反流病（GERD）：胃酸反流可能刺激呼吸道，引发慢性咳嗽和气短。  上气道咳嗽综合征（UACS）：通常由鼻后滴漏引起，表现为慢性咳嗽，可能伴有气短。  心力衰竭：心脏功能不全可能导致肺充血，引发气短和咳嗽，尤其在夜间或平躺时加重。  间质性肺病：这类疾病会导致肺组织纤维化，引起慢性咳嗽和进行性气短。  支气管扩张：气道结构异常导致慢性咳嗽、咳痰和气短。  肺癌：虽然不常见，但慢性咳嗽和气短也可能是肺癌的早期症状，尤其是有吸烟史的患者。  过敏性肺炎：长期暴露于过敏原（如霉菌、鸟类蛋白）可能导致慢性咳嗽和气短。  肺栓塞：血块阻塞肺动脉可能导致突发气短和咳嗽，通常伴有胸痛。  焦虑或恐慌障碍：心理因素也可能引发气短和咳嗽，尤其在紧张或焦虑时。  为了明确诊断，医生可能会建议进行肺功能测试、胸部影像学检查、血液检查等。请务必与医生详细沟通症状，以便制定合适的诊断和治疗计划。 | 4 | 4 | 4 | 4 | 4 | 4 | 4 |
| **Q3** | 怎么区分哮喘和其他疾病，比如慢性支气管炎或过敏性咳嗽？ | 区分哮喘、慢性支气管炎和过敏性咳嗽需要结合症状、病史和检查结果。以下是主要区别：  1. 哮喘  症状：反复发作的喘息、气短、胸闷和咳嗽，夜间或清晨加重，常由过敏原、冷空气、运动等诱发。  病史：常有过敏史或家族哮喘史。  检查：肺功能测试显示可变气流受限，支气管激发试验或舒张试验阳性。  2. 慢性支气管炎  症状：长期咳嗽、咳痰，持续至少3个月，连续2年以上，冬季加重。  病史：多见于吸烟者或长期接触粉尘者。  检查：肺功能测试显示持续气流受限，无显著可逆性。  3. 过敏性咳嗽  症状：干咳为主，无喘息或气短，接触过敏原后加重。  病史：有过敏史或家族过敏史。  检查：肺功能通常正常，支气管激发试验可能阳性，但无气流受限。  4. 其他辅助检查  过敏原测试：哮喘和过敏性咳嗽患者可能对特定过敏原敏感。  影像学检查：如胸部X光或CT，用于排除其他肺部疾病。  5. 治疗反应  哮喘：对支气管扩张剂和激素反应良好。  慢性支气管炎：治疗以控制症状为主，戒烟是关键。  过敏性咳嗽：抗组胺药和避免过敏原可缓解症状。  总结  哮喘：反复喘息，气流受限可逆。  慢性支气管炎：长期咳嗽咳痰，气流受限不可逆。  过敏性咳嗽：干咳为主，肺功能正常。  如有疑虑，建议尽早就医。 | 4 | 4 | 4 | 4 | 4 | 4 | 4 |
| **Q4** | 过敏性哮喘和支气管哮喘有什么不同？ | 咳嗽变异性哮喘（CVA）和过敏性咳嗽是两种常见的慢性咳嗽类型，尽管症状相似，但在病因、诊断和治疗上有所不同。  1. 咳嗽变异性哮喘（CVA）  定义：CVA是哮喘的一种特殊类型，主要表现为慢性干咳，无典型哮喘的喘息或呼吸困难。  病因：与气道高反应性和慢性炎症有关，诱因包括冷空气、运动、过敏原等。  症状：  持续干咳，夜间或清晨加重。  无喘息或呼吸困难。  对支气管扩张剂反应良好。  诊断：  肺功能测试显示气道高反应性。  支气管激发试验阳性。  排除其他慢性咳嗽原因。  治疗：  吸入糖皮质激素（ICS）。  长效β2受体激动剂（LABA）。  白三烯受体拮抗剂（如孟鲁司特）。  2. 过敏性咳嗽  定义：过敏性咳嗽是由过敏原引发的慢性咳嗽，无哮喘特征。  病因：常见过敏原包括尘螨、花粉、宠物皮屑等。  症状：  干咳为主，可能伴有鼻塞、流涕等过敏症状。  无喘息或呼吸困难。  咳嗽与特定环境或季节相关。  诊断：  过敏原检测（如皮肤点刺试验、血清IgE检测）。  排除其他慢性咳嗽原因。  治疗：  避免接触过敏原。  抗组胺药（如氯雷他定、西替利嗪）。  鼻用糖皮质激素（如布地奈德鼻喷雾剂）。  白三烯受体拮抗剂（如孟鲁司特）。  3. 区别  病因：CVA与气道高反应性和炎症相关，过敏性咳嗽则由过敏原引发。  症状：CVA主要为干咳，过敏性咳嗽可能伴有其他过敏症状。  诊断：CVA通过肺功能测试和支气管激发试验诊断，过敏性咳嗽依赖过敏原检测。  治疗：CVA主要使用吸入糖皮质激素和支气管扩张剂，过敏性咳嗽则侧重于抗组胺药和避免过敏原。  4. 注意事项  若慢性咳嗽持续，应及时就医，明确诊断后对症治疗。  避免自行用药，以免延误病情。  总结来说，CVA和过敏性咳嗽在病因、诊断和治疗上各有特点，需通过专业检查明确诊断并制定相应治疗方案。 | 4 | 4 | 4 | 4 | 4 | 4 | 4 |
| **Q5** | 如果我经常感觉喘不过气来，是否就是得了哮喘？还是有其他可能？ | 经常感觉喘不过气来并不一定意味着你得了哮喘，虽然哮喘确实可能导致这种症状，但还有许多其他可能性需要考虑。以下是一些可能的原因：  焦虑或恐慌发作：焦虑和恐慌可能导致呼吸急促、胸闷或喘不过气的感觉，通常伴有其他症状如心跳加速、出汗等。  慢性阻塞性肺病（COPD）：COPD包括慢性支气管炎和肺气肿，常见于长期吸烟者，症状包括呼吸困难、咳嗽和咳痰。  心脏问题：心力衰竭、心律失常等心脏疾病也可能导致呼吸困难，尤其是在活动或躺下时。  贫血：贫血会导致血液携氧能力下降，引发呼吸急促和疲劳。  肺部感染：如肺炎或支气管炎，可能导致呼吸困难，通常伴有发热、咳嗽等症状。  过敏反应：严重的过敏反应（如过敏性哮喘或过敏性休克）可能导致呼吸困难。  肥胖或缺乏锻炼：体重过重或体能较差也可能在日常活动中感到呼吸急促。  其他肺部疾病：如肺纤维化、肺动脉高压等，也可能导致呼吸困难。  胃食管反流病（GERD）：胃酸反流可能刺激呼吸道，引发类似哮喘的症状。  环境因素：暴露于空气污染、化学物质或过敏原也可能引发呼吸困难。  如果你经常感到喘不过气，建议尽早咨询医生，进行详细检查以确定具体原因。医生可能会通过肺功能测试、血液检查、心电图或影像学检查来帮助你找到病因，并制定合适的治疗方案。 | 4 | 4 | 4 | 4 | 4 | 4 | 4 |
| **Q6** | 我只有咳嗽不喘，医生说我得了哮喘，这样判断准确吗？ | 咳嗽是哮喘的常见症状之一，但并非唯一表现。哮喘通常还伴有喘息、胸闷和呼吸困难。然而，部分患者可能仅表现为咳嗽，称为“咳嗽变异性哮喘”。  医生的诊断通常基于以下依据：  病史：了解咳嗽的频率、持续时间及诱因。  体格检查：听诊肺部是否有异常呼吸音。  肺功能测试：评估气道阻塞及可逆性。  其他检查：如呼出气一氧化氮检测或支气管激发试验。  如果你对诊断有疑问，建议与医生进一步沟通，了解诊断依据，或考虑寻求第二意见。 | 4 | 4 | 4 | 4 | 4 | 4 | 4 |
|  | **COVID-19 Related** |  |  |  |  |  |  |  |  |
| **Q1** | 我是哮喘患者，感染新冠的风险是否比普通人高？我该怎么保护自己？ | 根据现有研究和临床观察，哮喘患者感染新冠病毒的风险与普通人相似，但若感染，哮喘症状可能会加重，导致病情复杂化。因此，哮喘患者需格外注意防护。  以下是一些保护建议：  继续用药：按医嘱使用哮喘控制药物，如吸入性 corticosteroids，确保病情稳定。  避免触发因素：远离已知的哮喘诱因，如过敏原、烟雾等。  保持社交距离：减少不必要的外出，避免人群聚集，降低感染风险。  佩戴 罩：在公共场所佩戴合适 罩，防止病毒传播。  勤洗手：使用肥皂和水洗手至少20秒，或使用含酒精的免洗洗手液。  接种疫苗：及时接种新冠疫苗和流感疫苗，减少感染和并发症风险。  健康生活方式：保持均衡饮食、适量运动、充足睡眠，增强免疫力。  监测症状：若出现哮喘加重或新冠症状（如发热、咳嗽、呼吸困难等），及时就医。  远程医疗：利用远程医疗咨询医生，减少医院暴露风险。  心理调适：保持积极心态，必要时寻求心理支持。  请根据个人情况和医生建议调整防护措施。 | 4 | 4 | 4 | 4 | 4 | 4 | 4 |
| **Q2** | 如果我得了新冠病毒，作为哮喘患者应该怎么处理？ | 如果你得了新冠病毒并且患有哮喘，以下是一些应对措施：  1. 及时就医  联系医生：立即告知你的医生，尤其是呼吸科或哮喘专科医生，获取专业建议。  紧急情况：如果出现呼吸困难、胸痛、嘴唇发紫等症状，立即拨打急救电话。  2. 监测症状  哮喘症状：注意哮喘是否加重，如喘息、胸闷、咳嗽等。  新冠症状：留意发热、咳嗽、乏力、味觉或嗅觉丧失等新冠症状。  3. 药物管理  继续用药：按医嘱使用哮喘药物，如吸入器和控制药物，不要自行停药。  备用药物：确保有足够的哮喘药物储备，尤其是急救药物。  4. 隔离与防护  居家隔离：避免与他人接触，防止病毒传播。  佩戴 罩：与家人同住时，尽量佩戴 罩，保持距离。  5. 保持良好生活习惯  充足休息：保证睡眠，帮助身体恢复。  健康饮食：多摄入富含维生素和矿物质的食物，增强免疫力。  适度活动：在身体允许的情况下，进行轻度活动，如散步。  6. 心理支持  保持联系：通过电话或网络与亲友保持沟通，缓解焦虑。  寻求帮助：如感到情绪困扰，及时寻求心理咨询。  7. 预防措施  接种疫苗：如未接种新冠疫苗，咨询医生后尽快接种。  避免感染：勤洗手、保持社交距离、避免人群聚集。  8. 紧急情况处理  呼吸困难：立即使用急救药物，如无效，立即就医。  持续高烧：如高烧不退或症状加重，及时就医。  总结  新冠病毒对哮喘患者可能带来更大风险，因此及时就医、严格管理哮喘、保持良好的生活习惯和心理状态至关重要。 | 4 | 4 | 4 | 4 | 4 | 4 | 4 |
| **Q3** | 我应该接种新冠疫苗吗？对哮喘有什么影响吗？ | 接种新冠疫苗对大多数人来说是安全且有益的，尤其是对于哮喘患者。以下是相关建议和影响：  1. 是否应该接种新冠疫苗？  推荐接种：新冠疫苗能有效降低感染、重症和死亡风险，尤其对哮喘患者更为重要，因为他们感染后可能出现更严重的症状。  适用性：大多数哮喘患者可以接种，但若对疫苗成分有严重过敏史，应咨询医生。  2. 对哮喘的影响  安全性：研究表明，新冠疫苗对哮喘患者是安全的，不会显著加重病情。  接种后反应：部分人可能出现轻微不适，如发热或疲劳，但这些反应通常短暂且可控。哮喘患者应继续按医嘱使用药物，接种后观察症状。  3. 注意事项  咨询医生：接种前最好与医生沟通，尤其是病情不稳定或近期有发作的患者。  过敏史：若有严重过敏史，医生会评估是否适合接种。  4. 总结  哮喘患者通常应接种新冠疫苗，以降低感染风险。  疫苗对哮喘患者安全，接种后需留意症状变化。  建议根据个人健康状况和医生建议做出决定。 | 4 | 4 | 4 | 4 | 4 | 4 | 4 |
| **Q4** | 哮喘患者感染新冠后的症状会比正常人更严重吗？需要特别注意什么？ | 哮喘患者感染新冠病毒后，症状可能比正常人更严重，因为哮喘本身是一种慢性呼吸道疾病，感染新冠可能加重呼吸道症状。以下是一些需要特别注意的事项：  1. 症状可能更严重  呼吸困难：哮喘患者感染新冠后，可能出现更严重的呼吸困难或哮喘发作。  咳嗽和胸闷：这些症状可能比普通人更明显。  缺氧风险：哮喘患者更容易出现血氧饱和度下降，需密切监测。  2. 特别注意的事项  坚持用药：继续使用哮喘控制药物，如吸入性糖皮质激素和支气管扩张剂。  监测症状：密切关注呼吸状况，若出现呼吸困难、胸痛等症状，及时就医。  避免诱发因素：远离烟雾、过敏原等可能加重哮喘的因素。  保持联系：与医生保持沟通，确保治疗方案适当。  接种疫苗：确保接种新冠疫苗和加强针，降低感染风险。  3. 紧急情况处理  若出现严重呼吸困难、嘴唇或面部发紫、意识模糊等症状，应立即就医或拨打急救电话。  4. 心理支持  感染新冠可能增加焦虑，哮喘患者应寻求心理支持，保持情绪稳定。  总之，哮喘患者感染新冠后需更加谨慎，密切监测症状并与医生保持联系，确保及时获得适当治疗。 | 4 | 4 | 4 | 4 | 4 | 4 | 4 |
|  | **Mental Health** |  |  |  |  |  |  |  |  |
| **Q1** | 我有哮喘，生活中总觉得很累，感到很无力，这正常吗？ | 我能理解你现在的感受。长期与哮喘共处确实会让人感到疲惫和无力，这是很正常的反应。你的身体在与疾病抗争，需要更多的能量和休息。不过，我们也可以采取一些措施来改善这种情况：  首先，确保你的哮喘得到了良好的控制。与医生密切配合，调整用药方案，可能会有所帮助。其次，适度的运动可以增强肺功能，但要循序渐进，选择适合自己的活动。充足的睡眠和均衡的饮食也很重要，它们能帮助你恢复体力。  你平时是如何管理哮喘的呢？有没有尝试过一些放松或呼吸练习来缓解疲劳感？和我分享一下你的经验吧，也许我们可以一起找到更多改善的方法。 | 4 | 4 | 4 | 4 | 4 | 4 | 4 |
| **Q2** | 哮喘影响了我的心情，我常常焦虑，应该怎么办？ | 我能理解哮喘给你带来的困扰和焦虑，这种感受确实让人沮丧和无助。首先，我想告诉你，你并不孤单。很多人都面临着类似的挑战，你的感受是完全正常和可以理解的。  面对这种情况，我建议你可以尝试以下几点：  与医生保持密切沟通，制定一个完善的哮喘管理计划。当你感觉病情得到良好控制时，焦虑也会随之减轻。  学习一些放松技巧，如深呼吸、冥想或瑜伽。这些方法可以帮助你在感到焦虑时平静下来。  与亲朋好友分享你的感受。倾诉不仅能缓解压力，还能获得他们的支持和理解。  保持适度的运动，如散步或游泳。运动不仅能改善肺功能，还能提升心情。  如果焦虑感持续影响你的生活，不妨考虑寻求心理咨询师的帮助。他们能提供专业的建议和支持。  记住，哮喘是可以管理的。通过积极治疗和调整生活方式，你完全可以过上充实快乐的生活。相信自己，你比想象中更坚强！如果需要倾诉或支持，随时告诉我，我会一直在这里。 | 4 | 4 | 4 | 4 | 4 | 4 | 4 |
| **Q3** | 哮喘让我总是担心会突然发作，是否有方法可以让我保持冷静？ | 我能理解你对哮喘发作的担忧，这种不安的感觉确实让人倍感压力。首先，我想告诉你，你并不孤单。很多人都面临着类似的挑战，而你已经很勇敢地在寻找应对方法了，这真的很棒。  让我们一起来探讨一些可以帮助你保持冷静的方法：  深呼吸练习：尝试缓慢而深长的呼吸，这能帮助缓解焦虑。你可以闭上眼睛，想象空气缓缓流入和流出身体。  正念冥想：每天花几分钟专注于当下，观察自己的呼吸和身体感受，这能帮助你建立内心的平静。  制定应急计划：随身携带急救药物，并熟悉哮喘发作时的应对步骤。有了准备，你会感觉更有掌控感。  与亲友沟通：让他们了解你的情况，这样在需要时他们可以给予支持和帮助。  保持健康生活方式：规律作息、适度运动和均衡饮食都有助于增强体质，减少发作风险。  寻求专业帮助：如果焦虑感持续，可以考虑咨询心理医生，他们能提供更多专业建议。  记住，哮喘是可以管理的。你已经迈出了重要的一步，那就是主动寻找解决方法。相信通过适当的预防和管理，你完全可以过上充实而快乐的生活。如果还有什么想聊的，我随时在这里倾听你的想法。 | 4 | 4 | 4 | 4 | 4 | 4 | 4 |
|  | **Lifestyle** |  |  |  |  |  |  |  |  |
| **Q1** | 我该怎么调整饮食，避免引发哮喘？ | 调整饮食可以帮助减少哮喘发作的风险，以下是一些建议：  1. 增加抗炎食物  富含Omega-3脂肪酸的食物：如三文鱼、沙丁鱼、亚麻籽、核桃等，有助于减轻炎症。  水果和蔬菜：尤其是富含维生素C和E的食物，如橙子、草莓、菠菜、西兰花等，具有抗氧化作用。  全谷物：如燕麦、糙米、全麦面包，有助于维持健康的免疫系统。  2. 减少促炎食物  加工食品：如薯片、快餐、甜点等，可能含有反式脂肪和高糖分，容易引发炎症。  高脂肪肉类：如红肉和加工肉类（香肠、培根等），可能加重炎症。  含添加剂的食物：如亚硫酸盐（常见于干果、酒类、腌制食品），可能诱发哮喘。  3. 避免过敏原食物  如果你对某些食物过敏（如牛奶、鸡蛋、坚果、海鲜等），尽量避免食用，以免引发哮喘。  4. 保持水分  多喝水有助于稀释呼吸道黏液，减少哮喘发作的可能性。  5. 控制体重  超重或肥胖可能加重哮喘症状，通过健康饮食和适量运动维持体重。  6. 注意饮食规律  避免暴饮暴食，尤其是睡前吃太多，可能引发胃酸反流，进而诱发哮喘。  7. 尝试地中海饮食  这种饮食模式以蔬菜、水果、鱼类、橄榄油和全谷物为主，具有抗炎作用，可能对哮喘患者有益。  8. 咨询医生或营养师  每个人的体质和哮喘诱因不同，建议根据个人情况制定饮食计划。  通过调整饮食，结合药物治疗和健康的生活方式，可以有效减少哮喘发作的频率和严重程度。 | 4 | 4 | 4 | 4 | 4 | 4 | 4 |
| **Q2** | 我的家里有宠物，会加重我的哮喘症状吗？ | 是的，家里有宠物可能会加重你的哮喘症状。宠物皮屑、唾液、尿液和毛发中的蛋白质是常见的过敏原，容易引发哮喘发作。尤其是猫和狗的皮屑较为细小，容易在空气中传播并被吸入，导致呼吸道敏感。  以下是一些建议，帮助你减少宠物对哮喘的影响：  保持清洁：定期清洁家居环境，使用高效空气净化器（HEPA过滤器）减少空气中的过敏原。  限制宠物活动区域：避免宠物进入卧室，尤其是床上，以减少过敏原的接触。  定期给宠物洗澡：每周给宠物洗澡可以减少皮屑和过敏原的积累。  勤洗手：接触宠物后及时洗手，避免将过敏原带到面部或呼吸道。  咨询医生：如果症状持续或加重，建议咨询医生，可能需要调整哮喘药物或进行过敏测试。  通过这些措施，你可以在享受宠物陪伴的同时，尽量减轻哮喘症状。 | 4 | 4 | 4 | 4 | 4 | 4 | 4 |
| **Q3** | 我可以做运动吗？哪些运动对哮喘患者更好？ | 可以运动，但需要选择合适的类型和强度。以下是对哮喘患者较为友好的运动：  推荐运动  游泳  温水泳池的湿润环境有助于减少呼吸道刺激，且游泳能增强心肺功能。  步行  低强度步行适合大多数哮喘患者，建议在空气清新的环境中进行。  瑜伽  瑜伽的呼吸练习有助于改善呼吸控制，减轻哮喘症状。  骑自行车  中等强度的骑行对心肺功能有益，注意避免空气污染严重的环境。  太极  动作缓慢，结合深呼吸，适合哮喘患者。  注意事项  热身和冷身：运动前后进行充分的热身和冷身，防止症状发作。  避免极端环境：寒冷、干燥或空气污染严重的环境可能诱发哮喘，尽量避免。  携带药物：运动时随身携带急救药物，如吸入器。  循序渐进：从低强度开始，逐步增加运动量。  不推荐的运动  高强度运动：如短跑、足球等，可能引发哮喘。  寒冷环境运动：如滑雪、滑冰等，冷空气可能刺激呼吸道。  总结  哮喘患者可以选择游泳、步行、瑜伽等低至中等强度的运动，注意环境和个人反应，运动时携带药物，确保安全。 | 4 | 4 | 4 | 4 | 4 | 4 | 4 |
| **Q4** | 我体重比较重，能通过减肥来改善哮喘症状吗？ | 减肥可能有助于改善哮喘症状，尤其是对于超重或肥胖的患者。研究表明，体重减轻可以带来以下好处：  减轻肺部压力：多余的体重会增加肺部负担，减肥后呼吸会更轻松。  减少炎症：肥胖常伴随慢性炎症，减肥有助于降低炎症水平，从而缓解哮喘。  提高药物效果：减肥可能增强哮喘药物的疗效。  改善整体健康：减肥还能降低其他健康风险，如高血压和糖尿病，间接帮助控制哮喘。  注意事项：  咨询医生：在开始减肥前，建议先咨询医生，确保计划安全且适合你的健康状况。  合理饮食与运动：选择健康的饮食和适度的运动，避免剧烈运动引发哮喘发作。  持续监测：减肥过程中应密切监测哮喘症状，及时调整治疗方案。  总之，减肥可能对改善哮喘有帮助，但需在医生指导下进行。 | 4 | 4 | 4 | 4 | 4 | 4 | 4 |
| **Q5** | 作为哮喘患者，我平时生活中应该注意哪些事项？ | 作为哮喘患者，日常生活中需注意以下事项，以有效控制病情并减少发作：  1. 避免诱发因素  远离过敏原：如尘螨、花粉、宠物皮屑、霉菌等。  避免空气污染：减少接触烟雾、汽车尾气等。  防止呼吸道感染：勤洗手，避免接触感冒或流感患者。  注意气候变化：寒冷或干燥空气可能引发哮喘，外出时佩戴口罩。  2. 规律用药  遵医嘱用药：按时使用控制药物（如吸入性糖皮质激素）和急救药物（如短效β2受体激动剂）。  定期复查：与医生保持沟通，调整治疗方案。  3. 监测病情  使用峰流速仪：定期监测呼气峰流速，了解病情变化。  记录症状：记录发作时间、频率和诱因，帮助医生评估病情。  4. 保持健康生活方式  适度运动：选择游泳、散步等温和运动，避免剧烈活动。  均衡饮食：多摄入水果、蔬菜，避免易过敏食物。  控制体重：肥胖可能加重哮喘症状。  戒烟：吸烟会加剧哮喘，避免二手烟。  5. 心理调节  减轻压力：压力可能诱发哮喘，可通过冥想、深呼吸等方式放松。  保持积极心态：哮喘可控，积极面对有助于病情管理。  6. 应急准备  随身携带急救药物：如沙丁胺醇吸入器，以备不时之需。  制定应急计划：与医生制定发作时的应对措施，严重时立即就医。  7. 改善居住环境  保持室内清洁：定期清洁，减少尘螨和霉菌。  使用空气净化器：改善室内空气质量。  避免刺激性气味：如香水、清洁剂等。  8. 接种疫苗  接种流感疫苗：预防呼吸道感染。  接种肺炎疫苗：降低肺炎风险。  通过这些措施，可以有效控制哮喘，减少发作，提升生活质量。如有疑问，及时咨询医生。 | 4 | 4 | 4 | 4 | 4 | 4 | 4 |
| **Q6** | 我在换季时常常会出现哮喘症状加重，除了增加药物治疗外，我还能做些什么来减轻  症状？ | 换季时哮喘症状加重是常见现象，除了增加药物治疗，你还可以采取以下措施来减轻症状：  避免过敏原：换季时花粉、尘螨等过敏原增多，尽量减少接触。外出时戴口罩，回家后及时更换衣物并洗脸。  保持室内空气清洁：使用空气净化器，定期清洁空调滤网，避免灰尘和霉菌积聚。  控制湿度：保持室内湿度在30%-50%之间，避免过湿或过干，防止霉菌和尘螨滋生。  适度锻炼：选择适合的锻炼方式，如游泳或瑜伽，增强肺功能。避免在寒冷或空气污染严重时户外运动。  饮食调理：多吃富含维生素C和抗氧化剂的食物，如水果、蔬菜，减少易引发过敏的食物。  保暖防寒：天气变化时注意保暖，尤其是呼吸道，避免冷空气直接刺激。  定期复诊：与医生保持沟通，定期检查，及时调整治疗方案。  学习呼吸技巧：掌握腹式呼吸等技巧，帮助缓解急性发作时的呼吸困难。  减少压力：压力可能诱发哮喘，尝试冥想、深呼吸等方式放松。  戒烟并避免二手烟：吸烟会加重哮喘，戒烟并远离二手烟环境。  如果症状持续或加重，务必及时就医。 | 4 | 4 | 4 | 4 | 4 | 4 | 4 |
|  |  |  |  |  |  |  |  |  |  |
