# Supplementary material for: Assessing the Role of Large Language Models Between ChatGPT and DeepSeek in Asthma Education for Bilingual Individuals: Comparative Study
Source: JMIR Med Inform. 2025 Aug 13;13:e65365. doi: 10.2196/65365 (PMC12349887; doi:10.2196/65365)
Supplement: Multimedia Appendix 5 [file medinform-v13-e65365-s005.doc]

|  | ChatGPT EN | ChatGPT EN | ChatGPT CN | ChatGPT CN | DeepSeek EN | DeepSeek EN | DeepSeek CN | DeepSeek CN |
| --- | --- | --- | --- | --- | --- | --- | --- | --- |
| Consistency | 3.924528302 | 0.266678761 | 3.981132075 | 0.137360564 | 3.98113208 | 0.13736056 | 3.96226415 | 0.19238025 |
| Completeness | 3.849056604 | 0.411200233 | 3.886792453 | 0.319878424 | 3.96226415 | 0.19238025 | 3.98113208 | 0.13736056 |
| Potential Bias | 3.962264151 | 0.192380248 | 3.981132075 | 0.137360564 | 4 | 0 | 4 | 0 |
| Reasoning Ability | 3.981132075 | 0.137360564 | 4 | 0 | 4 | 0 | 3.98113208 | 0.13736056 |
| Comprehension | 3.962264151 | 0.192380248 | 4 | 0 | 3.98113208 | 0.13736056 | 4 | 0 |
| Reliability | 3.962264151 | 0.192380248 | 3.981132075 | 0.137360564 | 4 | 0 | 4 | 0 |
| Safety | 3.962264151 | 0.192380248 | 4 | 0 | 4 | 0 | 4 | 0 |

Supplementary File 5: Comparative Scores of Two AI Models Across Multiple Evaluation Dimensions in a Bilingual Context
